# Modular Toolkit of Multifunctional Block Copoly(2‐oxazoline)s for the Synthesis of Nanoparticles

**DOI:** 10.1002/chem.202101327

**Published:** 2021-05-05

**Authors:** Philipp Keckeis, Enriko Zeller, Carina Jung, Patricia Besirske, Felizitas Kirner, Cristina Ruiz‐Agudo, Helmut Schlaad, Helmut Cölfen

**Affiliations:** ^1^ Physical Chemistry University of Konstanz Universitätstrasse 10, Box 714 78457 Konstanz Germany; ^2^ Institute of Chemistry University of Potsdam Karl-Liebknecht-Strasse 24–25 14476 Potsdam Germany

**Keywords:** block copolymers, click chemistry, nanoparticles, ring-opening polymerization, surfactants

## Abstract

Post‐polymerization modification provides an elegant way to introduce chemical functionalities onto macromolecules to produce tailor‐made materials with superior properties. This concept was adapted to well‐defined block copolymers of the poly(2‐oxazoline) family and demonstrated the large potential of these macromolecules as universal toolkit for numerous applications. Triblock copolymers with separated water‐soluble, alkyne‐ and alkene‐containing segments were synthesized and orthogonally modified with various low‐molecular weight functional molecules by copper(I)‐catalyzed azide‐alkyne cycloaddition (CuAAC) and thiol‐ene (TE) click reactions, respectively. Representative toolkit polymers were used for the synthesis of gold, iron oxide and silica nanoparticles.

## Graphical abstract

**Polyoxazoline block copolymers** offer a wide range of possibilities for application depending on their molecular structure and properties. This study introduces the huge potential of this polyoxazoline toolkit and demonstrates how tailor‐made toolkit polymers can be applied in representative systems.

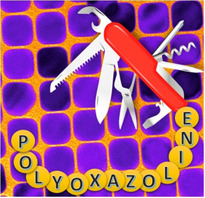

Multifunctional block copolymers can synergistically improve the properties of materials, which is the reason why they are used in all scientific disciplines from physicochemical material science up to biological applications in the medicinal course. Particularly, the poly(2‐oxazoline) (POx) polymer family is of great interest due to its architectural versatility, tunable polarity and property, biocompatibility and wide range of chemical functionalization.^[1]^ The sequential living cationic ring opening polymerization (CROP) of 2‐oxazolines enables the synthesis of well‐defined block copolymers with predictable molar masses (up to about 300 incorporated monomer units) and narrow molar mass distributions of each segment.^[2]^ Hence, a large selection of POxs with specific properties and functionalities can be obtained by the incorporation of 2‐alkyl‐2‐oxazoline monomers with different chemical side groups.^[3]^ However, not every chemical functionality is tolerated in cationic polymerizations, though this limitation can be overcome by the use of protecting group chemistry or by suitable post‐polymerization modification approaches. For the latter, the modification of POxs carrying either alkyne or alkene side chains has been successfully achieved using “click” chemistry,^[4]^ namely by copper(I)‐catalyzed azide‐alkyne cycloaddition (CuAAC)^[5]^ or by thiol‐ene (TE)^[6]^ reactions, respectively, profiting from high yields and mild reaction conditions. In this regard, Schubert and coworkers introduced a multifunctional copoly(2‐oxazoline) scaffold containing anthracene and azide termini and two alkene side chains for triple orthogonal click post‐polymerization modifications by Diels‐Alder cycloaddition, CuAAC and TE click reactions.^[7]^ This sophisticated orthogonal coPOx system is complemented by our versatile toolkit enabling high degrees of modification by equipping whole segments with the desired functionalities. Functional POx have been applied to modify the surface of inorganic nanoparticles,^[8]^ however, the literature for the grafting‐onto approach is limited. Nevertheless, some inspiring examples are available for iron oxide using phosphate linkers,^[9]^ silica using trimethoxysilane linkers,^[10]^ and gold nanoparticles using thiol linkers.^[11]^

Herein, we combine well‐established CuAAC and TE click reactions into one universal synthetic protocol for the orthogonal post‐polymerization modification of triblock coPOxs aiming to generate a modular toolkit of multifunctional polymers (Figure 1). Specific chemical functionalities can be introduced to the separated alkyne‐ and alkene‐containing block segments in order to equip the polymer segments with charges (COOH or NH_2_), hydroxyl groups (OH) or fluorescence labels (Rhodamine B), respectively, but also with chemical linkers. The latter include the introduction of desired binding groups onto the polymer such as thioethers (SAc), siloxanes (Si(OMe)_3_), catechols (Ph(OH)_2_) or carboxylates (COOH) as chemical ligands for the interaction with different inorganic systems such as gold, metal(‐loid) hydroxides, iron oxide and other appropriate positively‐charged surfaces. Moreover, we provide straightforward recipes for the synthesis of polymer‐coated gold, iron oxide and silica nanoparticles with help of the newly tailor‐made coPOxs.

**Figure 1:**
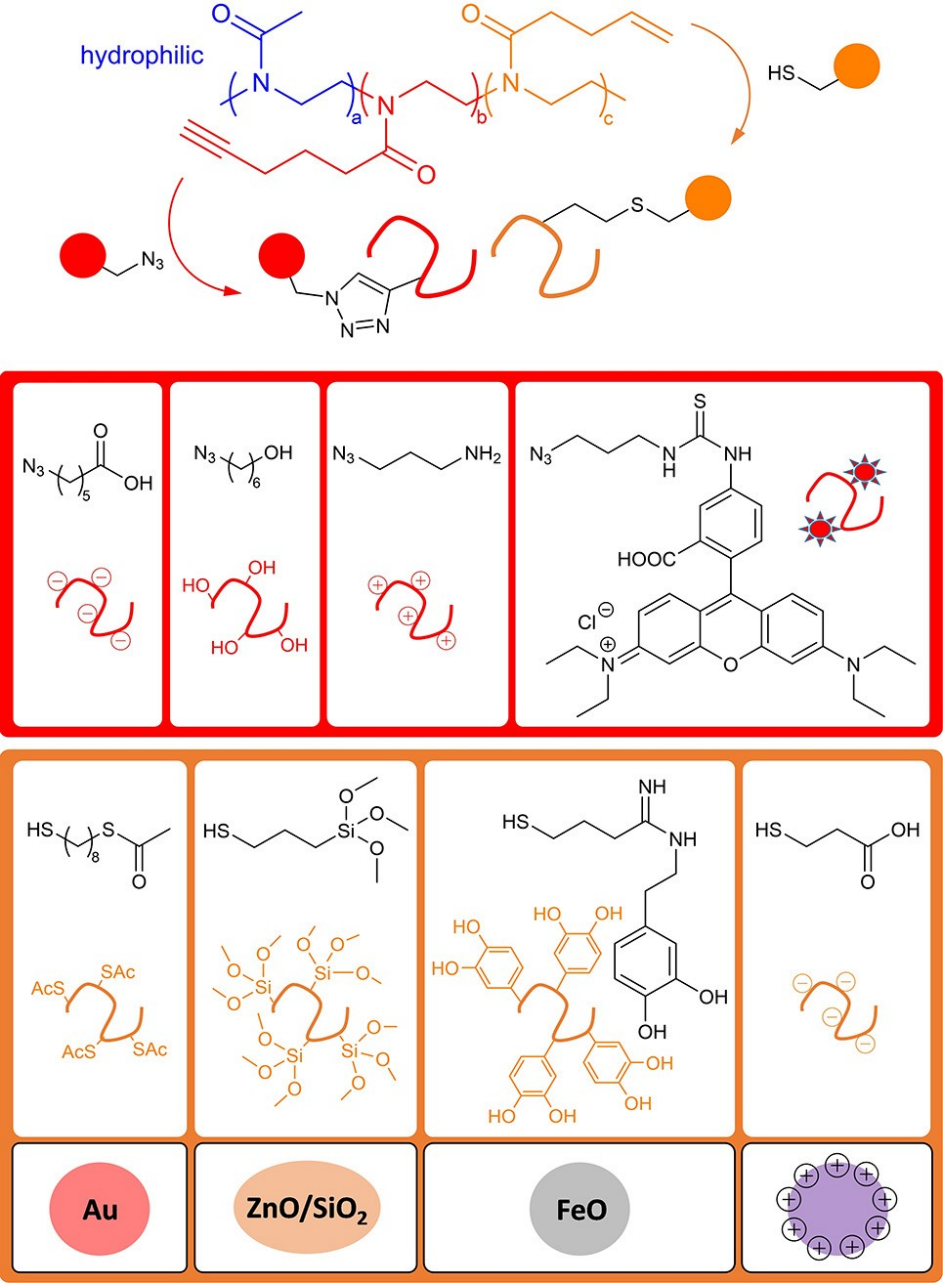
Schematic representation of the modular toolkit representing the orthogonal post‐polymerization modification of a poly(2‐oxazoline)‐based triblock copolymer PMeOx‐PPentynOx‐PButenOx via azide‐alkyne cycloaddition (CuAAC) and thiol‐ene (TE) click reaction as well as potential systems for nanotechnological application.

The synthetic pathway towards the modular polymer toolkit starts with the synthesis of the functionalizable monomers 2‐(4‐pentynyl)‐2‐oxazoline (PentynOx) and 2‐(3‐butenyl)‐2‐oxazoline (ButenOx) (see Supporting Information, section 2).[^5^, ^6^, ^12^] The sequential microwave‐assisted CROP^[13]^ of 2‐methyl‐2‐oxazoline (MeOx), PentynOx, and ButenOx in acetonitrile solution at 140 °C (initiator was methyl triflate and the reaction time for every monomer was 30 min) produced a series of triblock copoly(2‐oxazoline)s consisting of a hydrophilic block (PMeOx) and two consecutive blocks with alkyne (PPentynOx) and alkene (PButenOx) moieties (see Supporting Information, section 3). The molecular compositions were determined by ^1^H NMR spectroscopy and the dispersity by size exclusion chromatography. Notably, the block copolymer samples exhibited somewhat higher dispersities, that is in the range of 1.2–1.4, which can be attributed to the highly concentrated and thus viscous reaction solutions. Exemplary two precursor samples PMeOx_43_‐PPentynOx_11_‐PButenOx_11_ (**P1**) and PMeOx_52_‐PPentynOx_32_‐PButenOx_13_ (**P3**) (were used for subsequent post‐polymerization modifications with low‐molecular weight azido and thiol molecules by orthogonal CuAAC and TE click reactions (see Table 1 and Supporting Information, sections 4 and 5).

**Table 1:** List of multifunctional triblock copoly(2‐oxazoline)s obtained by post‐polymerization modification of PMeOx‐PPentynOx‐PButenOx with low molecular weight azides and thiols via consecutive azide‐alkyne cycloaddition (CuAAC) and thiol‐ene (TE) reactions; numbers in parenthesis correspond to the degrees of modification for each modification step.

Precursor	CuAAC	TE	Product
P1	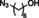	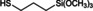	P1‐OH−Si(OMe)_3_
	(100 %)	(55 %)	
P3	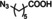	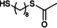	P3‐COOH‐SAc
	(80 %)	(100 %)	
P3	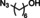	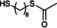	P3‐OH‐SAc
	(100 %)	(100 %)	
P3	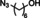	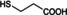	P3‐OH‐COOH
	(100 %)	(100 %)	
P3	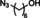	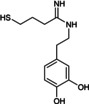	P3‐OH−Ph(OH)_2_
	(100 %)	(15 %)

The orthogonal modification of **P3** to give **P3‐COOH‐SAc** (which was later used as surfactant for gold nanocubes, see below) is described as representative example of the polymer toolkit.

## First post‐polymerization modification: CuAAC

To guarantee orthogonality, CuAAC must be carried out first before the TE click reaction, since thiols can react with both alkynes (under formation of 1,2‐dithio compounds)^[14]^ and alkenes during UV exposure following a radical pathway.

**Figure 2:**
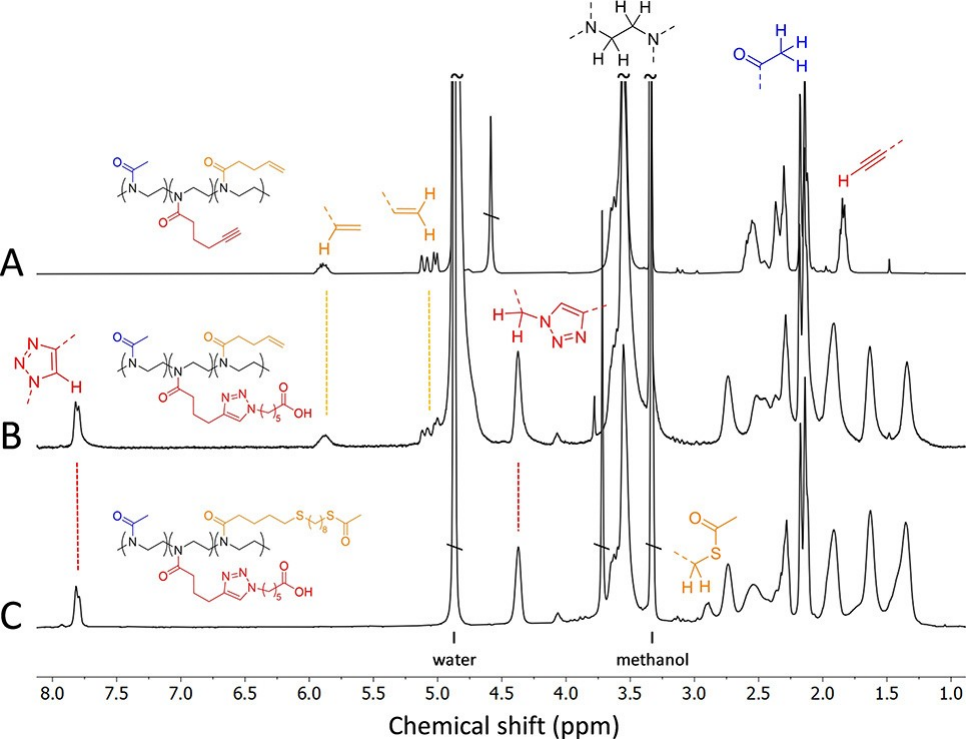
^1^H NMR spectra (400 MHz, CD_3_OD) of A) PMeOx_52_‐PPentynOx_32_‐PButenOx_13_ (**P3**), B) PMeOx_52_‐PPentynOx_32_(COOH)_26_‐PButenOx_13_ after attaching 6‐azido‐hexanoic acid via CuAAC and C) PMeOx_52_‐PPentynOx_32_(COOH)_26_‐PButenOx_13_(SAc)_13_ (**P3‐COOH‐SAc**) after attaching *S*‐(8‐mercaptooctyl)ethanethioate via TE click reaction.

The CuAAC reaction between the triblock copolymer **P3** and 6‐azido‐hexanoic acid was promoted by copper(II)sulfate and ascorbic acid in a water/*tert*‐butanol mixture (see Supporting Information). The copper ions were afterwards removed by dialysis against EDTA‐solution, which turned out as indispensable procedure to avoid a green coloration of the polymers, that disturbs subsequent analyses. The modified copolymer was analyzed by ^1^H NMR spectroscopy (Figure 2). The appearance of the triazole proton signal at 7.8 ppm and the signal of the neighboring methylene group at 4.4 ppm showed the successful attachment of carboxylates. The degree of modification of **P3** alkyne units was found to be about 80 %, by considering the peak integrals of the vinyl and triazole protons, corresponding to 26 carboxylate units on the PPentynOx segment.

## Second post‐polymerization modification: TE

To attach *S*‐(8‐mercaptooctyl)ethanethioate to the PButenOx segment, the carboxylate‐modified copolymer was dissolved in THF/methanol solution and exposed to UV irradiation (*λ*=356 nm) for two days in the presence of the photoinitiator 2,2‐dimethoxy‐2‐phenylacetophenone (see Supporting Information). The ^1^H NMR analysis of the dialyzed polymer product suggested a quantitative modification of the 13 alkene units as the signals of the vinyl protons at 5.05/5.86 ppm were completely absent (Figure 2). Quantification of the peak integrals at 2.9 ppm (−CH_2_‐SAc) and 4.4 ppm (−CH_2_‐triazole) confirmed the number of 26 attached carboxylate units, which however excludes that the thiol reacted with residual alkyne units. Accordingly, the final polymer **P3‐COOH‐SAc** exhibits the chemical structure PMeOx_52_‐PPentynOx_32_(COOH)_26_‐PButenOx_13_(SAc)_13_.

Notably, as documented in Table 1, some of the CuAAC or TE modification reactions were not quantitative, hence were not truly “click” reactions, for yet unknown reasons. Particularly the TE reaction with *N*‐(3,4‐dihydroxyphenethyl)‐4‐mercaptobutaneimidamide) gave a yield of just 15 % corresponding to two catechol groups attached to the PButenOx segment. Nevertheless, the attachment of catechol derivatives by a radical TE reaction is remarkable because catechols are otherwise known and used as radical scavengers or inhibitors.

## Exemplary application of the block copoly(2‐oxazoline) toolkit

Ultimately, selected polymer architectures of the POx toolkit were applied in colloidal model systems in order to chemisorb on gold and iron oxide nanoparticle surfaces or in the Stöber synthesis of silica nanoparticles. Surface interaction represents a key role for numerous nanoparticle applications focusing for instance on particle stabilization, crystallization events, polarity tuning, visualization, etc.

**Figure 3:**
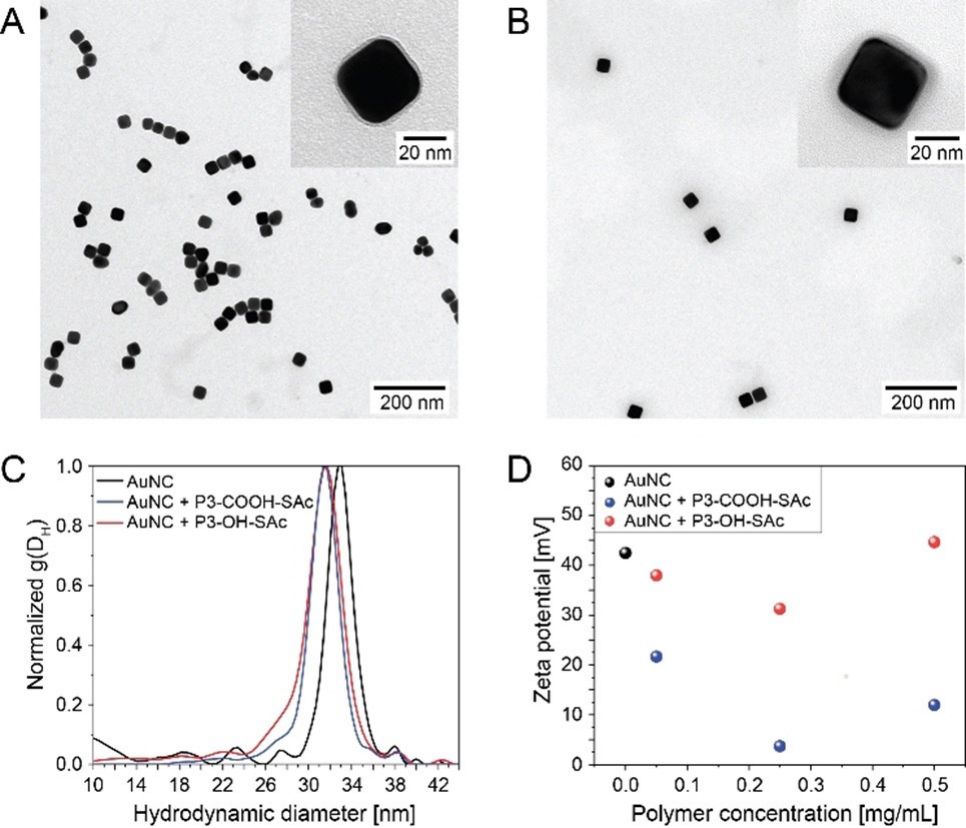
Surface‐functionalization of CPC‐stabilized AuNC using **P3‐COOH‐SAc** (PMeOx_52_‐PPentynOx_32_(COOH)_26_‐PButenOx_13_(SAc)_13_) and **P3‐OH‐SAc** (PMeOx_52_‐PPentynOx_32_(OH)_32_‐PButenOx_13_(SAc)_13_). TEM analyses of the polymer‐gold nanocube composites **P3‐COOH‐SAc** (A) and **P3‐OH‐SAc** (B). AUC sedimentation experiments (C) of CPC‐stabilized AuNC (black) and AuNC‐polymer composites (**P3‐COOH‐SAc**, blue and **P3‐OH‐SAc**, red). Zeta‐potential measurement (D) of CPC‐stabilized AuNC (black) and AuNC‐polymer composites (**P3‐COOH‐SAc**, blue and **P3‐OH‐SAc**, red) with increasing total polymer concentrations.

Addressing gold nanoparticles, an aqueous dispersion of freshly synthesized, cetylpyridinium chloride (CPC) ‐ stabilized gold nanocubes (AuNC)^[15]^ with an average edge length of 34 nm was mixed with an aqueous solution of **P3‐COOH‐SAc**. The thioester (SAc) group was selected due to the known ability to chemisorb on gold surfaces.^[16]^ After removing non‐bound polymer by centrifugation, the AuNC‐polymer composite was analyzed with complementary structural techniques (see Figure 3 and Supporting Information, section 6.1).

UV/Vis spectroscopy indicated minute shifts of the absorption maxima from 529 to 530 nm suggesting the colloidal stability of the AuNC‐polymer composites, free of aggregation (Figure S52).^[17]^ Analytical ultracentrifugation (AUC) sedimentation‐velocity experiments revealed a significant shift of the AuNC sedimentation coefficient in the presence of **P3‐COOH‐SAc** to lower values (Figure 3C, blue line). As the total particle density decreases after the adsorption of polymer and the friction of the polymer shell increases with respect to the initial CPC stabilizer, the shift of the sedimentation coefficient with otherwise unchanged distribution suggests the presence of stable, organic polymer‐coated AuNCs without aggregation.^[18]^ In addition, the resemblance of the sedimentation profiles points towards the morphological conservation of the nanoparticles. Zeta‐potential measurements reveal a significant reduction of the positive value of CPC‐stabilized AuNCs in the presence of the polymer emphasizing the macromolecular surface modification and ion complex formation between the CPC ammonium and the polymer carboxylate (Figure 3D). This experiment was repeated with the multifunctional block copolymer **P3‐OH‐SAc** (PMeOx_52_‐PPentynOx_32_(OH)_32_‐PButenOx_13_(SAc)_13_) having a polyhydroxyl middle segment instead of polycarboxylate and the zeta potential stayed in the region of 40 mV as for the initial CPC‐stabilized AuNCs (Figure 3D). This shows that the block copolymer is adsorbed additionally to the CPC stabilizer without a full ligand exchange. The AUC experiments reveal identical sedimentation profiles of the two different gold polymer conjugates (Figure 3C). This gives rise to the conclusion that the SAc is the key to surface linkage independent of the presence of COOH or OH groups. Moreover, transmission electron microscopy (TEM) analysis depicts the existence of a ring with lower contrast surrounding the AuNC in the case of both polymers (Figure 3A and 3B) indicating the presence of a surface coating with polymer.

Addressing iron oxide nanoparticles (IONP), the two polymers **P3‐OH‐COOH** and **P3‐OH‐Ph(OH)_2_** were used to modify the surfaces of the as‐synthesized IONP with a size of 9 nm, as described by Kang *et al*.^[19]^ Carboxylate and catechol functionalities were selected due to their well‐known ability to chemisorb on Fe_3_O_4_,^[20]^ having an isoelectric point of 6.8.^[21]^ In general, carboxylic^[21]^ and catechol^[22]^ groups were found to adsorb stronger on iron oxide surfaces at alkaline milieu via complexation of Fe surface atoms.^[23]^ TEM, AUC and TGA analysis (Supporting Information, section 6.2) suggested the presence of polymer‐coated nanoparticles with organic contents of 4.5 % (**P3‐OH‐COOH**) and 15.5 % (**P3‐OH‐Ph(OH)_2_**), respectively. The high polymer content observed with **P3‐OH‐Ph(OH)_2_** supports the very good binding ability of catechol groups to IONPs,^[24]^ much better than that of carboxylates, and demonstrates the successful application of the toolkit polymers to colloidal iron oxide.

Finally we were addressing silica nanoparticles, which are widely used as carrier systems in biomedical applications,^[25]^ in sensor techniques^[26]^ or as rheological additives.^[27]^ **P1‐OH‐Si(OMe)_3_** was used as a macromolecular silica precursor agent in the Stöber‐synthesis of silica nanoparticles. The hydrolysis and condensation reaction yielded to 15 nm‐sized SiO_2_ nanoparticles (Supporting Information, section 6.3). Dynamic light scattering experiments showed the absence of aggregation. In a more general consideration, this example presents the opportunity to produce silica particles with selected surface functionalities (e. g. carboxy, hydroxy, amine or fluorescent molecules) that have been attached to the block copolymer via CuAAC.

In conclusion, an orthogonal system for the post‐polymerization modification of poly(2‐oxazoline)‐based triblock copolymers has been developed. The two distinct segments of alkyne and alkene blocks were subsequently modified by copper(I)‐catalyzed azide‐alkyne cycloaddition (CuAAC) and thiol‐ene (TE) click reaction. Various low‐molecular‐weight molecules were attached to the chemical handles pursuing specific interactions with inorganic surfaces. Apart from CuAAC and TE, the alkyne and alkene blocks can alternatively be modified by using Pd^0^‐catalyzed C−C cross coupling using the Sonogashira (triple bonds) and Heck reactions (double bonds); preliminary results are shown in the Supporting Information (section 5.3). To this end, the triblock copolymer was equipped with functional groups, such as carboxylates, amines, hydroxyl groups, fluorescent Rhodamine B, thioacetates, catechols and silanes as shown in the overview Table S1 containing 14 examples of multifunctional polymers. The combination of these specific functionalities defines the modular toolkit for tailored specific applications, which exemplarily was demonstrated for gold, iron oxide and silica nanosystems. Therefore, the non‐toxic post‐functionalized polyoxazolines bear the potential to enter various nanotechnological applications, for instance, the field of drug delivery in terms of modifying, stabilizing and visualizing nanoconjugates or alternatively guiding the drug loaded micelle by a magnetic field due to attached magnetite nanoparticles. Another application would be nanomedicine where one block is modified with a target molecule to attach to tumors, while the second block has binders for gold or magnetite nanoparticles, which would destroy the tumor by light‐ or magnetic field‐generated heat. Also, a combination of a cholesterol‐functionalized block and an acidic block could be used to dissolve atherosclerotic plaques, as we have demonstrated for poly‐peptide‐based triblock copolymers.^[28]^

These examples just demonstrate a small part of the possibilities with the here developed modular toolkit, because any combination from the following applications should be feasible: self‐assembly, templating, nanoparticle stabilization, surface modification, scale inhibition, solubilization, staining, drug‐delivery (or more general molecular delivery) or nanomedical applications. Ultimately, the binary orthogonal scaffold introduced in this work could even be extended to a multi‐dimensional system applying multiple orthogonal click post‐polymerization reactions.

## Experimental Section

Detailed descriptions of the synthesis of the modular polymer toolkit and procedures for the functionalization of inorganic nanoparticles are provided in the Supporting Information.

## Conflict of interest

The authors declare no conflict of interest.

## Supporting information

As a service to our authors and readers, this journal provides supporting information supplied by the authors. Such materials are peer reviewed and may be re‐organized for online delivery, but are not copy‐edited or typeset. Technical support issues arising from supporting information (other than missing files) should be addressed to the authors.

Supplementary
