# Supplementary material for: Modular Toolkit of Multifunctional Block Copoly(2‐oxazoline)s for the Synthesis of Nanoparticles
Source: Chemistry. 2021 May 5;27(32):8283–7. doi: 10.1002/chem.202101327 (PMC8252465; doi:10.1002/chem.202101327)
Supplement: Supplementary file 1 — Supplementary [file CHEM-27-8283-s001.pdf]

Chemistry–A European Journal

Supporting Information

Modular Toolkit of Multifunctional Block Copoly(2-oxazoline)s for the Synthesis of Nanoparticles

Philipp Keckeis, Enriko Zeller, Carina Jung, Patricia Besirske, Felizitas Kirner, Cristina Ruiz-Agudo, Helmut Schlaad, and Helmut Cölfen*

Table of content

1 Materials and Methods 4

2 Synthesis of the 2-oxazoline monomers 6

2.1 Synthesis of 2-(4-pentynyl)-2-oxazoline (PentynOx) 7

2.2 Synthesis of 2-(but-3-enyl)-2-oxazoline (ButenOx) 9

3 Synthesis of block copoly(2-oxazoline)s with alkyene and alkene segments 10

4 Synthesis of azide and thiol molecules with specific functional groups 15

4.1 Synthesis of azide molecules 15

4.1.1 Synthesis of 6-azido-hexanoic acid (COOH-N_3) [4] 15

4.1.2 Synthesis of 6-azido-hexanol (OH-N_3) [4] 16

4.1.3 Synthesis of 3-azido-propyl amine (Amine-N_3) [4] 17

4.1.4 Synthesis of 1-(3-azidopropyl)-3-RhodamineB thiourea (RhoB-N_3) [5] 18

4.2 Synthesis of thiol molecules 19

4.2.1 Synthesis of (*N*-(3,4-dihydroxyphenethyl)-4-mercaptobutanimidamide) (Cat-SH) 19

4.2.2 Synthesis of *S*-(8-mercaptooctyl)ethanethioate (SAc-SH) [7] 21

5 Post-polymerization modification (PPM) 23

5.1 1. PPM: Copper-catalyzed alkyne-azide cycloaddition 24

5.1.1 COOH-modification 24

5.1.2 OH-modification 26

5.1.3 NH_2-modification 27

5.1.4 RhoB-modification 29

5.2 2. PPM: Thiol-ene click reaction 30

5.2.1 COOH-modification 30

5.2.2 Catechol-modification 31

5.2.3 Thioester-modification 32

5.2.4 Siloxane-modification 34

5.3 3. PPM: Palladium(0) catalyzed C-C cross coupling 36

5.3.1 Sonogashira coupling reaction 36

5.3.2 Heck coupling reaction 37

6 Synthesis of polymer-coated nanoparticles 38

6.1 Gold 38

6.1.1 Synthesis of gold nanocubes 38

6.1.2 Surface-modification of gold nanocubes 38

6.2 Iron oxide **40**

6.2.1 Synthesis of iron oxide nanoparticles 40

6.2.2 Surface-modification of iron oxide nanoparticles 40

6.3 Silica Nanoparticles **42**

6.3.1 Dialysis-triggered silica formation 42

6.3.2 Silica formation by a Stöber process 43

7 References ***45***

1 Materials and Methods

Materials. 2-methyl-2-oxazoline (MeOx) (>98%), 1,8-octanedithiol (>98%), 3-mercaptopropionic acid (>99%), 2-iminothiolane hydrochloride (>98%), cysteamine (>95%), sodium azide (>99.5%), Rhodamine B thioisocyanate, 2-chloroethylammonium chloride (99%), methyl triflate (MeOTf) (>98%), piperidine (≥99.5%), *n*-butyl lithium (2.5 M hexane), sodium ascorbate (98%), sodium azide (99.5%), ethylenediaminetetraacetic acid (EDTA) (>98%), 4-iodobenzotrifluoride (>97%), methyl-4-iodobenzoate (>97%) and 2,2-dimethoxy-2-phenylacetophenone (DMPA) (99%) were purchased from Sigma Aldrich. 5-hexynoic acid (96%), allyl bromide (99%), diisopropylamine (99%), 3-bromopropylamine hydrobromide (98%), N-hydroxysuccinimide (98%), 6-bromo-1-hexanol (97%), 6-bromohexanoic acid (98%), (3-mercaptopropyl)trimethoxysilane (98%), dopamine hydrochloride (>99%) were obtained from Alfa Aesar. 1-(3-Dimethylpropyl)-3-ethylcarbodiimide hydrochloride (EDAC) (99.4%) was purchased from Iris-Biotech. Diethylether was purchased from VWR chemicals. Acetic acid anhydride (99%) was purchased from Acros Organics. Silica gel (0.4-0.063 mm) and *tert*-butanol were obtained from Carl Roth. Copper(II)sulfate pentahydrate and triethylamine (>99%) were purchased from Merck. Chloroform-d (99.8%) and Methanol-d4 (CD3OD) were purchased from Deutero GmbH. Dialysis membranes (Spectra/Por® 6, pre-wetted RC tubing, MWCO: 1 kD) were purchased from Spectrumlabs.

Unless stated otherwise, all reactions were carried out under inert gas atmosphere using standard Schlenk or glovebox techniques. Milli-Q water was prepared by Milli-Q Millipore Direct 8 system (resistivity < 18.2 µS/cm). Chemicals and solvents were purchased from different suppliers and used as received, if not noted otherwise. Dimethyl formamide (DMF), Dichloromethane (DCM) and diisopropylamine were distilled from CaH_2, methanol was distilled from magnesium, diethylether was distilled from sodium, tetrahydrofuran was distilled from blue sodium/benzophenone ketyl and acetonitrile was distilled from CaH_2 and stored under nitrogen over molecular sieve (3A) prior to use. MeOTf and MeOx were freshly distilled from CaH_2.

Methods. Polymerization mixtures were prepared in a glovebox under N_2 atmosphere with <1 ppm water and O_2 contents. Microwave-assisted polymerizations were performed with an Anton Paar Monowave 300 at 140 °C (30 min for each block).

Nuclear magnetic resonance (NMR) spectra were recorded on a Varian Inova 400 or a Bruker Avance 400 spectrometer. ^{1}H and ^{13}C chemical shifts are given in ppm and are referenced to the solvent signals. The ^{1}H NMR spectra of polymer conjugates were measured with a relaxation delay of 6 s and an acquisition time of 4 s. Data were processed and analyzed using MestReNova software.

Elemental analyses (CHN) were performed on an Elementar Vario EL instrument up to 950 °C.

Attenuated total reflection infrared spectroscopy (ATR-IR) was performed on a Perkin Elmer spectrometer equipped with a universal ATR sampling accessory.

Gas chromatography (GC) was carried out on a PerkinElmer Clarus 500 instrument with an autosampler and FID detection on a PerkinElmer Elite-5 (5% Diphenyl- 95% Dimethylpolysiloxane) Series Capillary Column (Length: 30 m, Inner Diameter: 0.25 mm, Film Thickness: 0.25 mm), using helium as the carrier gas at a flow rate of 1.5 mL/min.

Electrospray ionization – mass spectrometry (ESI-MS) spectra were recorded on a Bruker microTOF focus II mass spectrometer coupled to a Dionex 3000 UHPLC (RP-C18, water/acetonitrile, 0,1% formic acid).

Gel permeation chromatography (GPC) with simultaneous UV and RI detection was performed with *N*-methyl-2-pyrrolidone (NMP + 0.5 wt% LiBr) as the eluent at a flow rate of 0.5 mL/min at room

temperature. The stationary phase was a 300×8 mm^2 PSS-GRAM linear column (7 µm particle size, molar mass range 10^2-10^6 Da). Solutions containing ~0.15 wt% polymer were filtered through 0.45 µm PTFE filters. Calibration was done with polystyrene standards.

Analytical ultracentrifugation (AUC) sedimentation velocity experiments were performed on a Beckman-Coulter XL-I using the Rayleigh interference optics at 25 °C.

Dynamic light scattering (DLS). Particle sizes of the polymer associates were investigated with a Zetasizer Nano ZSP (Malvern Instrument, Malvern, U.K.) using a He/Ne laser (λ = 633 nm) and a scattering angle of 173°.

Zeta potentials of as-prepared polymer dispersions in Milli-Q water (0.1 wt%) were measured on a Malvern Instruments Zetasizer Nano-ZS Zen3600.

Transmission electron microscopy (TEM) was performed on a Zeiss Libra 120 EF-TEM instrument operating at 120 kV. Samples were prepared by application of a drop of aqueous particle dispersion (0.05 wt%) to a carbon-coated grid and evaporation of water.

2 Synthesis of the 2-oxazoline monomers

The olefin and alkyne 2-oxazoline monomers were synthesized according to protocols described by the groups of Schlaad [1] and Jordan [2], respectively (**Scheme S1**).

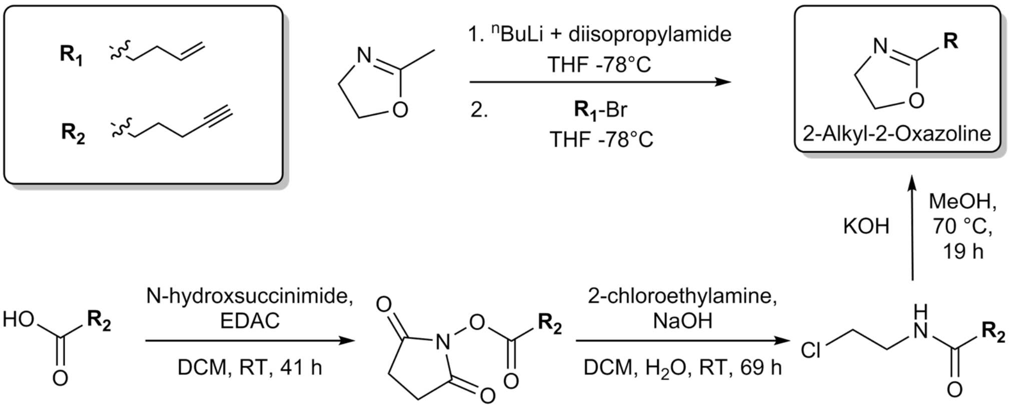

Scheme S1. Synthetic pathway for 2-(3-butenyl)-2-oxazoline (ButenOx, **R1**) and 2-(4-pentynyl)-2-oxazoline (PentynOx, **R2**).

2-(3-butenyl)-2-oxazoline (ButenOx) monomer was synthesized in a one-pot synthesis via α-deprotonation of 2-methyl-2-oxazoline before adding allyl bromide at -78 °C, as reported by Dargaville *et al.* [3]

2-(4-pentynyl)-2-oxazoline (PentynOx) was synthesized as described by Schlaad and coworkers with a slightly optimized protocol. [1] The experimental details and characterization data are provided below (section 2.1 and 2.2).

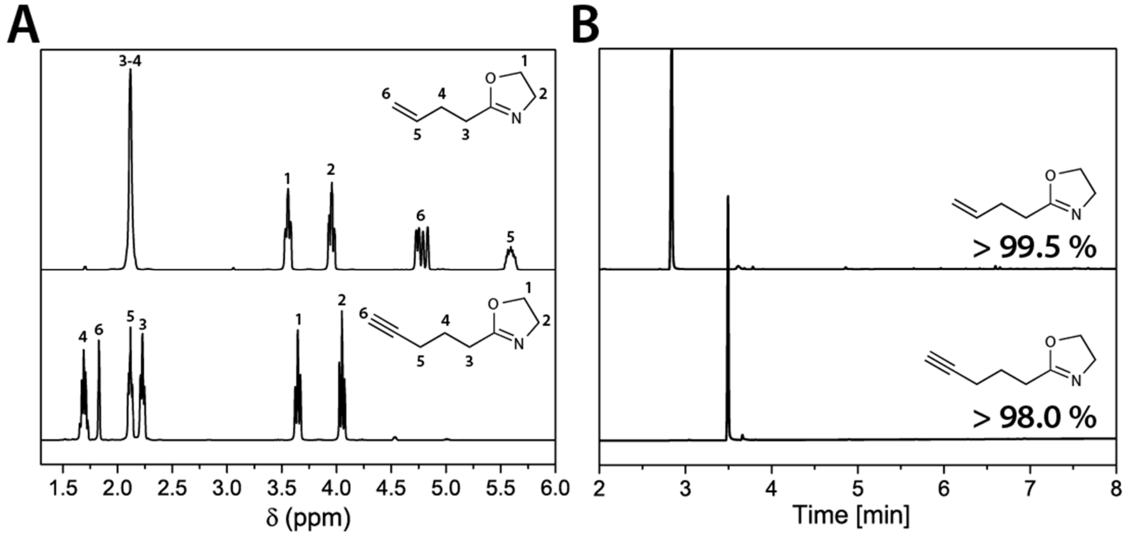

Figure S1 A) ^{1}H NMR spectra (400 MHz, $CDCl_3$) of 2-(3-butenyl)-2-oxazoline (ButenOx, top) and 2-(4-pentynyl)-2-oxazoline (PentynOx, bottom). **B**) Gas chromatograms of ButenOx (top) and PentynOx (bottom).

2.1 Synthesis of 2-(4-pentynyl)-2-oxazoline (PentynOx)

The synthesis of 2-(4-pentynyl)-2-oxazoline (overall yield 60%) included three steps starting from succinimidyl-activation (1) of 4-pentynoic acid followed by the formation of N-(2-chloroethyl)-pentynoyl amide (2) prior to the cyclization of 2-(4-pentynoyl)-2-oxazoline (3), based on a similar procedure described elsewhere. [1]

(1) *N*-Succinimidyl-5-hexynate.

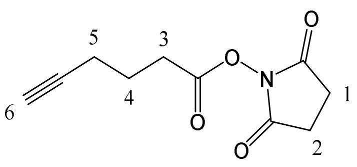

In a flame dried flask, N-hydroxysuccinimide (41.06 g, 356.8 mmol) and EDAC (51.23 g, 267.5 mmol) were dissolved in 1100 mL of dry DCM. 4-Hexynoic acid (25.00 g, 223.0 mmol) was added to the solution and the reaction was stirred at room temperature for 41 h. After evaporation of the solvent, the residue was dissolved in 500 mL of Et_2O, washed 3 times with 200 mL of distilled water and dried over Na_2SO_4 prior to removing the solvent. A colorless solid was obtained (yield: 41.67 g, 198 mmol, 89%). **^{1}H NMR** (400 MHz, $CDCl_3$): δ (ppm) = 2.79 (s, 4H, H^{1+2}), 2.73 (m, 2H, H^3), 2.31 (m, 2H, H^5), 2.00 (t, ^3J = 2.64 Hz, 1H, H^6), 1.92 (pd, ^3J_p = 7.12 Hz, ^3J_d = 1.16 Hz, 2H, H^4).

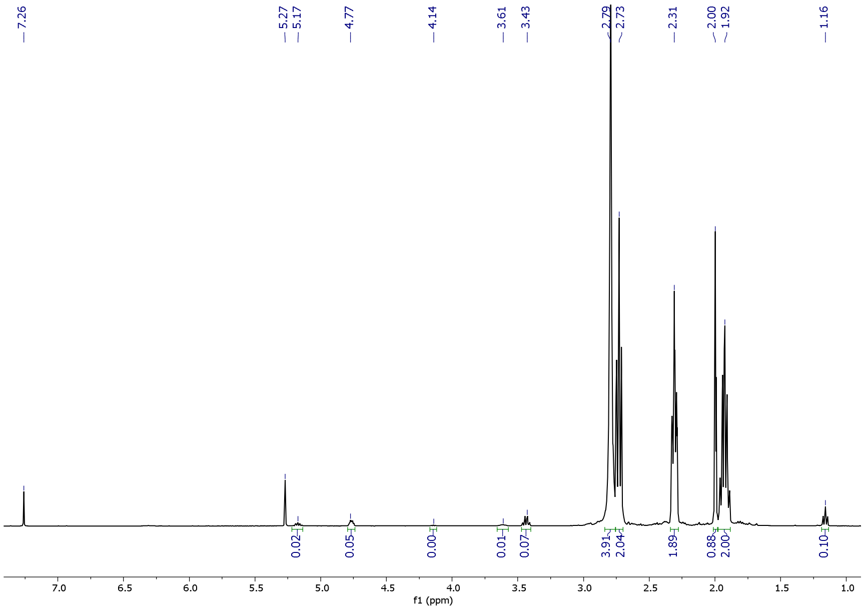

Figure S2: ^{1}H NMR (400 MHz, $CDCl_3$) spectrum of *N*-succinimidyl-5-hexynate.

(2) N-(2-Chloroethyl)-5-hexynamide.

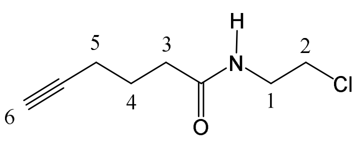

To a solution of NaOH (16.00 g, 399 mmol) and 2-chloroethyl-ammonium chloride (46.31 g, 399 mmol) in 500 mL of distilled water, N-succinimidyl-pentynoylate (41.76 g, 199.6 mmol) dissolved in 1050 mL of DCM was added dropwise under vigorous stirring. After the reaction was stirred for 3 d, the organic layer was separated, washed three times with 200 mL of distilled water and dried over Na_2SO_4. After removing the solvent, a pale-yellow solid was obtained (yield: 30.05 g, 173.7 mmol, 87%). **^{1}H NMR** (400 MHz, $CDCl_3$): δ (ppm) = 6.21 (br, 1H, H^7), 3.57 (m, 4H, H^{1+2}), 2.23 (t, ^3J = 7.52 Hz, 2H, H^3), 2.22 (m, 2H, H^5), 1.96 (m, 1H, H^6), 1.82 (pd, ^3J_p = 7.12 Hz, ^3J_d = 2.08 Hz, 2H, H^4).

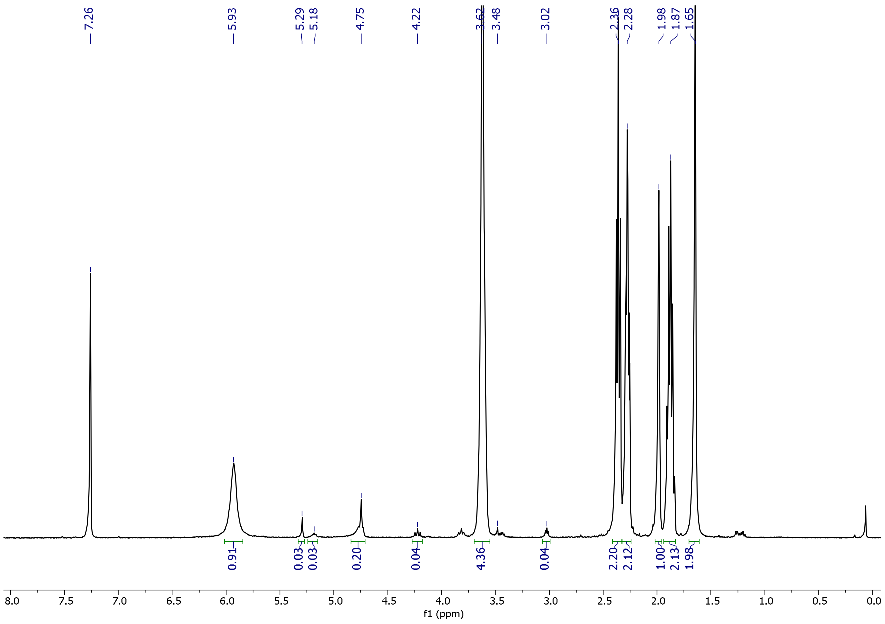

Figure S3: ^{1}H NMR (400 MHz, $CDCl_3$) spectrum of *N*-(2-chloroethyl)-5-hexynamide.

(3) 2-(Pent-4-ynyl)-2-oxazoline.

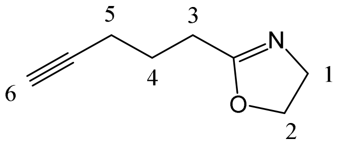

N-(2-Chloroethyl)-pentynoyl amide (30.00 g, 173 mmol) was dissolved in 30 mL of dry methanol. 20 mL of a freshly prepared solution of KOH (7.00 g, 173 mmol) in dry methanol were added dropwise. After stirring the reaction at 70 °C for 18 h, the precipitate was removed by filtration and the solution was diluted in 500 mL of diethyl ether. The organic phase was washed with deionized water (4 x 400 mL). The solvent was removed *in vacuo* and the yellow residue was distilled under reduced pressure to yield a colorless liquid (bp^{1mbar}: 50–54 °C; 16.5 g, 121.1 mmol, 70%). **^{1}H NMR** (400 MHz, $CDCl_3$): δ (ppm) = 4.05 (t, ^3J = 9.56 Hz, 2H, H^2), 3.65 (t, ^3J = 9.40 Hz, 2H, H^1), 2.23 (td, ^3J_t = 7.40 Hz, ^3J_d = 0.84 Hz, 2H, H^3), 2.12 (m, 2H, H^5), 1.83 (m, 1H, H^6), 1.69 (pt, ^3J_p = 7.32 Hz, 2H, H^4). **GC**: retention time: 3.49 min, (>98%). **ATR-IR**: 3301 (≡C─H str) (m), 2954 (C─H str) (w), 1666 (C═O str) (s), 1414 (CH_2─CO) (m), 638 (≡C─H wag) (s). **ESI-MS:** m/z calc.: 137.08, found: 137.08 + H^+.

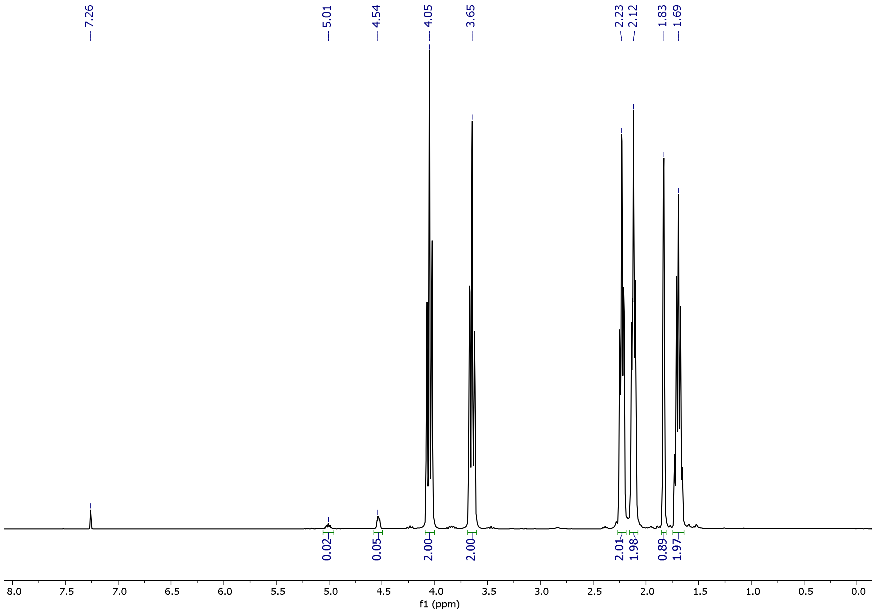

Figure S4: ^{1}H NMR (400 MHz, $CDCl_3$) spectrum of 2-(pent-4-ynyl)-2-oxazoline.

2.2 Synthesis of 2-(but-3-enyl)-2-oxazoline (ButenOx)

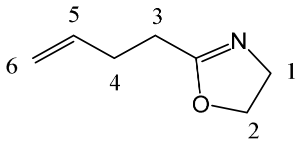

In a flame-dried flask, a mixture of THF (110 mL) and *n*-butyllithium in hexane (39.2 mL, 2.5 M, 98 mmol) was cooled to -78 °C in a nitrogen atmosphere. Diisopropylamide (14.5 mL, 103.17 mmol) was added dropwise. During this addition, the temperature was kept below -65 °C. 2-Methyl-2-oxazoline (8.74 mL, 103.17 mmol) was added carefully to the freshly prepared LDA (lithium diisopropylamide) solution. The temperature of the mixture was kept below -65 °C during the addition and was stirred for 1 hour at -78 °C. Allyl bromide (8.91 mL, 103.2 mmol) was added dropwise and stirred at -78 °C for further 60 min prior to stirring overnight at room temperature. Afterwards the reaction was quenched with 40 mL methanol. The solvent was removed under reduced pressure and the residue was dissolved in dichloromethane and water. The organic layer was washed three times with 100 mL water and 100 mL brine. The combined organic layers were dried over $MgSO_4$ and the solvent was removed under reduced pressure. The crude product was further purified by distillation. A colorless liquid was obtained (bp^{1mbar}: 59 °C; 10.75 g, 83%). **^{1}H NMR** (400 MHz, $CDCl_3$): δ (ppm) = 5.59 (m, 1H, H^5), 4.76 (m, 2H, H^6), 3.96 (t, ^3J = 9.48 Hz, 2H, H^2), 3.56 (t, ^3J = 9.16 Hz, 2H, H^1), 2.12 (s, 4H, H^{3+4}). **GC**: retention time: 2.81 min (>99.5%). **ATR-IR**: 3079 (C=CH_2 str) (m), 2972 (C–H str) (w), 1666 (C=O str) (s), 1640 (C=C str) (m). **ESI-MS:** m/z calc: 125.08, found: 125.08 + H^+.

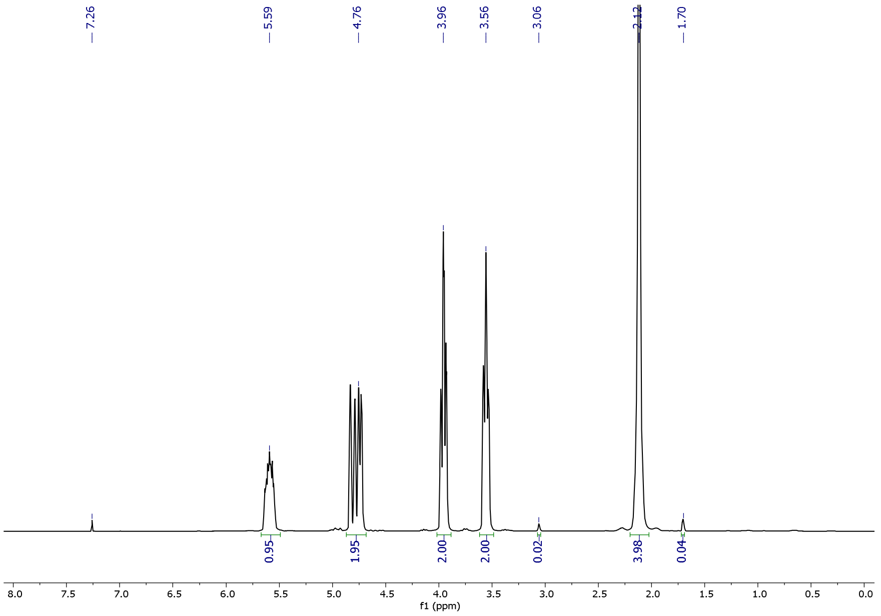

Figure S5: ^{1}H NMR (400 MHz, $CDCl_3$) spectrum of 2-(but-3-enyl)-2-oxazoline.

3 Synthesis of block copoly(2-oxazoline)s with alkyene and alkene segments

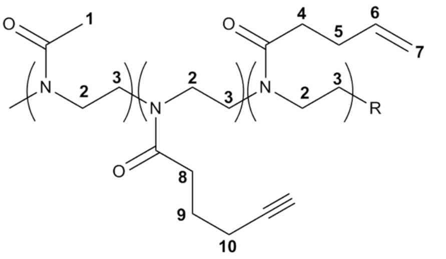

Microwave-assisted polymerization. All compounds were added to the microwave reactor (10 mL) in a glovebox. For the first block, 2-methyl-2-oxazoline (MeOx) and anhydrous acetonitrile (monomer concentration ~ 2-3 M) were added into a flame-dried microwave-reactor. The MeOTf initiator was added and the reactor was transferred to microwave. The microwave-reaction was heated up to 140 °C within 5 min and then stirred at 140 °C for 30 min. The mixture was cooled down to room temperature prior to the addition of the second monomer 2-(4-pentynyl)-2-oxazoline (PentynOx) in the glovebox. The second block was polymerized by irradiating the reaction mixture with the same microwave conditions. For the third block, the mixture was cooled down to room temperature prior to the addition the third monomer 2-(3-butenyl)-2-oxazoline (ButenOx) in the glovebox. The polymerization was conducted under the same microwave conditions. The polymerization was terminated with distilled water or piperidine. The obtained mixtures were purified by dialysis against methanol first and then against dist. water (MWCO 1000 g/mol). After freeze-drying, the polymers were obtained as colorless powders.

P1: $PMeOx_{43}$-$PentynOx_{11}$-$PButenOx_{11}$: MeOTf (34.5 µL, 0.3 mmol), MeOx (1.29 g, 15.0 mmol), PentynOx (0.42 g, 3 mmol), ButenOx (0.38 g, 3 mmol), *N*-Boc-piperazine (0.167 g, 0.9 mmol). Yield: 1.17 g. **^{1}H NMR** (400 MHz, $CDCl_3$): δ (ppm) = 5.83 (m, 10.8H, H^6), 5.03 (m, 21.3H, H^7), 3.49 (m, 258H, H^{2-3}), 3.05-2.94 (m, 3H, $H^{Initiator}$), 2.46 (m, 30.4H, H^8), 2.37 (m, 87H, H^{4-5}), 2.28 (m, 35H, H^{10}), 2.15 (m, 129H, H^1), 1.83 (m, 22H, H^9). **GPC** (NMP, PS cal.): M_n = 8700 g/mol, $Đ$ = 1.40.

P2: $PMeOx_{50}$-$PentynOx_9$-$PButenOx_9$: MeOTf (34.5 µL, 0.3 mmol), MeOx (1.29 g, 15.2 mmol), PentynOx (0.38 g, 2.7 mmol), ButenOx (0.34 g, 2.7 mmol). Yield: 0.67 g. **^{1}H NMR** (400 MHz, $CDCl_3$): δ (ppm) = 5.81 (m, 9.1H, H^6), 5.03 (m, 17.9H, H^7), 3.46 (m, 270H, H^{2-3}), 3.05-2.94 (m, 3H, $H^{Initiator}$), 2.48 (m, 21H, H^8), 2.37 (m, 36H, H^{4-5}), 2.28 (m, 20H, H^{10}), 2.15 (m, 150H, H^1), 1.83 (m, 18H, H^9). **GPC** (NMP, PS cal.): M_n = 9270 g/mol, $Đ$ = 1.16.

P3: $PMeOx_{52}$-$PentynOx_{32}$-$PButenOx_{13}$: MeOTf (34.5 µL, 0.3 mmol), MeOx (1.29 g, 15.0 mmol), PentynOx (1.24 g, 9.0 mmol), ButenOx (0.38 g, 3.0 mmol). Yield: 1.64 g. **^{1}H NMR** (400 MHz, CD_3OD): δ (ppm) = 5.87 (m, 12.2H, H^6), 5.03 (m, 26.9H, H^7), 3.56 (m, 386.8H, H^{2-3}), 3.15-2.97 (m, 3H, $H^{Initiator}$), 2.63-2.25 (m, 216H, $H^{8+4-5+10}$), 2.14 (m, 158H, H^1), 1.85 (m, 64H, H^9), 1.48 (m, 2.5H, H^{BOC}). **GPC** (NMP, PS cal.): M_n = 11790 g/mol, $Đ$ = 1.43.

P4: $PMeOx_{57}$-$PButenOx_8$: MeOTf (13.6 µL, 0.12 mmol), MeOx (0.51 g, 6.0 µmol), ButenOx (0.14 g, 1.1 mol). Yield: 0.58 g. **^{1}H NMR** (400 MHz, $CDCl_3$): δ (ppm) = 5.81 (m, 7.9H, H^6), 5.01 (m, 16.8H, H^7), 3.44 (m, 263H, H^{2-3}), 3.05-2.94 (m, 3H, $H^{Initiator}$), 2.39 (m, 51H, H^{4-5}), 2.09 (m, 172H, H^1). **GPC** (NMP, PS cal.): M_n = 6480 g/mol, $Đ$ = 1.40.

P1: PMeOx$_{43}$-PentynOx$_{11}$-PButenOx$_{11}$

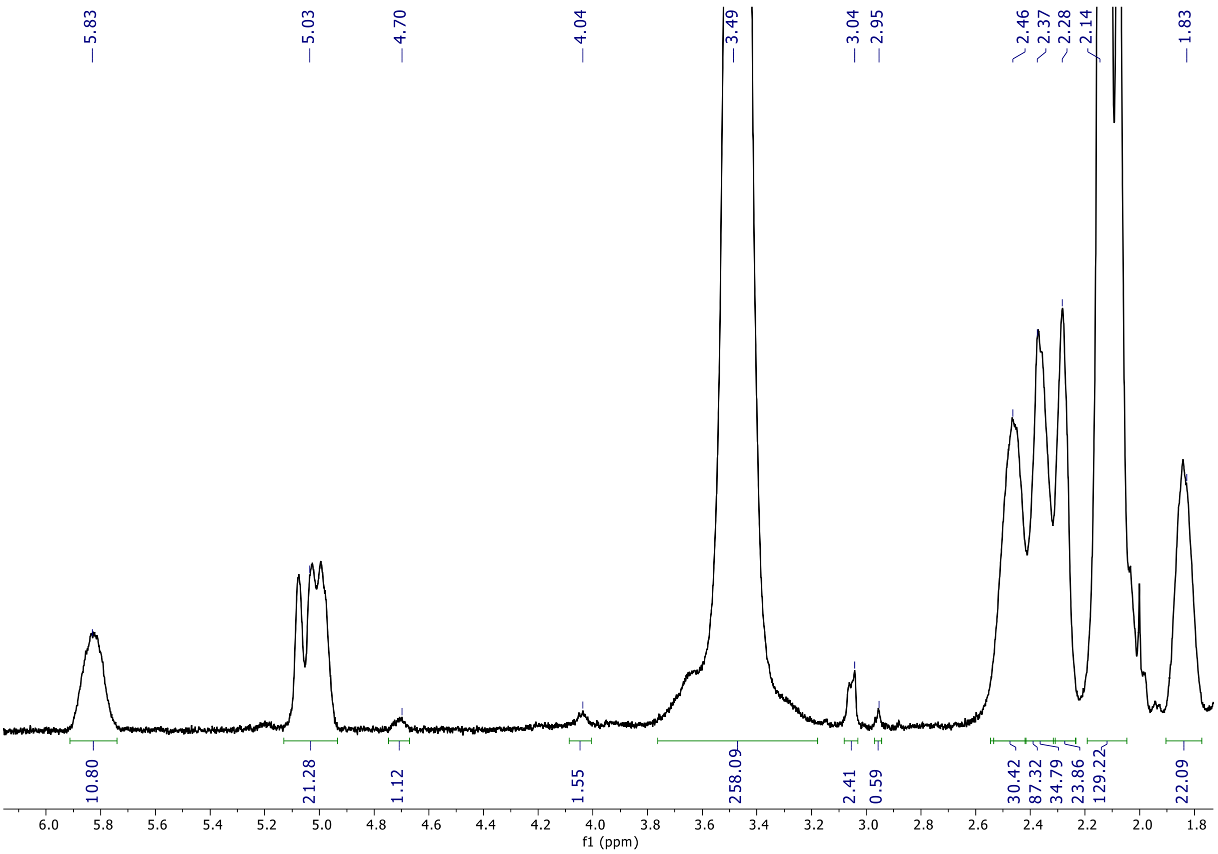

Figure S6. ^{1}H NMR (400 MHz, $CDCl_3$) spectrum of PMeOx$_{43}$-PentynOx$_{11}$-PButenOx$_{11}$.

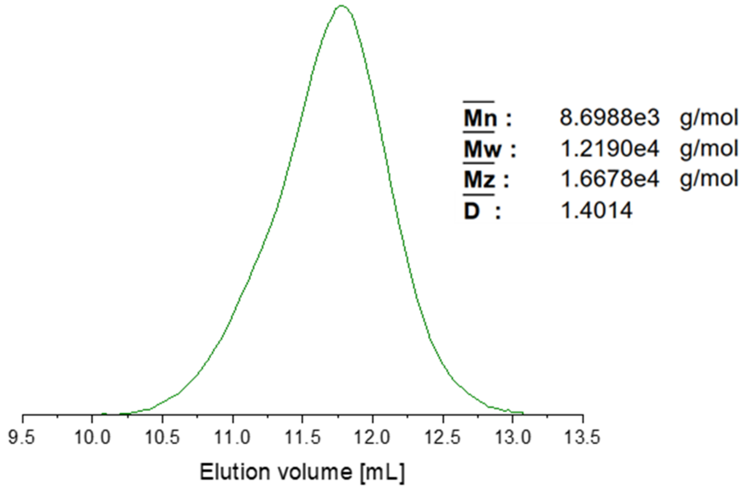

Figure S7: GPC RI trace (eluent: NMP) of PMeOx$_{43}$-PentynOx$_{11}$-PButenOx$_{11}$.

P2: PMeOx$_{50}$-PPentynOx$_9$-PButenOx$_9$

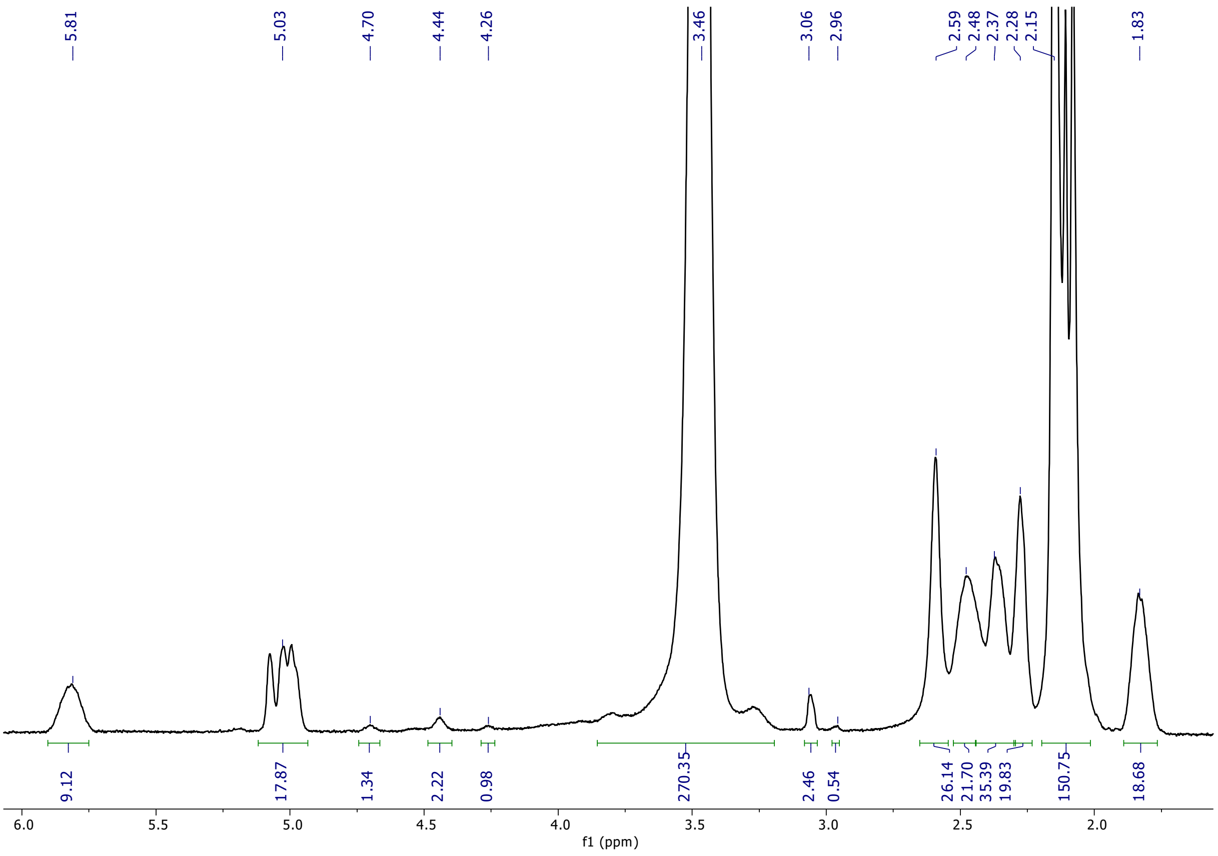

Figure S8: ^{1}H NMR (400 MHz, $CDCl_3$) spectrum of PMeOx$_{50}$-PPentynOx$_9$-PButenOx$_9$.

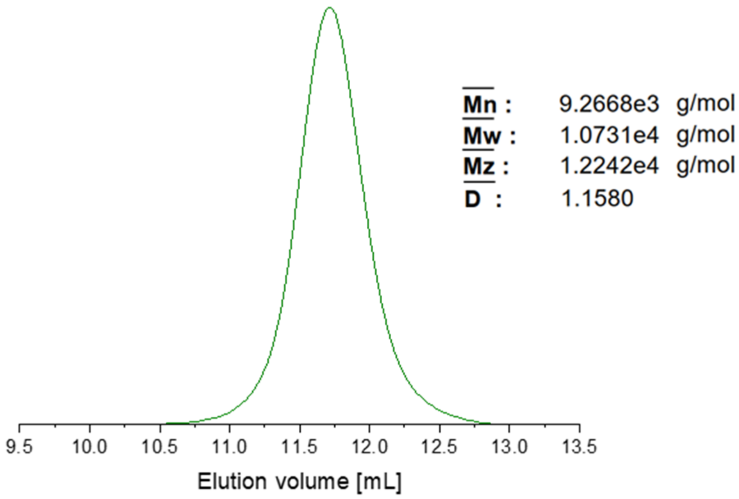

Figure S9: GPC RI trace (eluent: NMP) of PMeOx$_{50}$-PPentynOx$_9$-PButenOx$_9$.

P3: PMeOx$_{52}$-PPentynOx$_{32}$-PButenOx$_{13}$

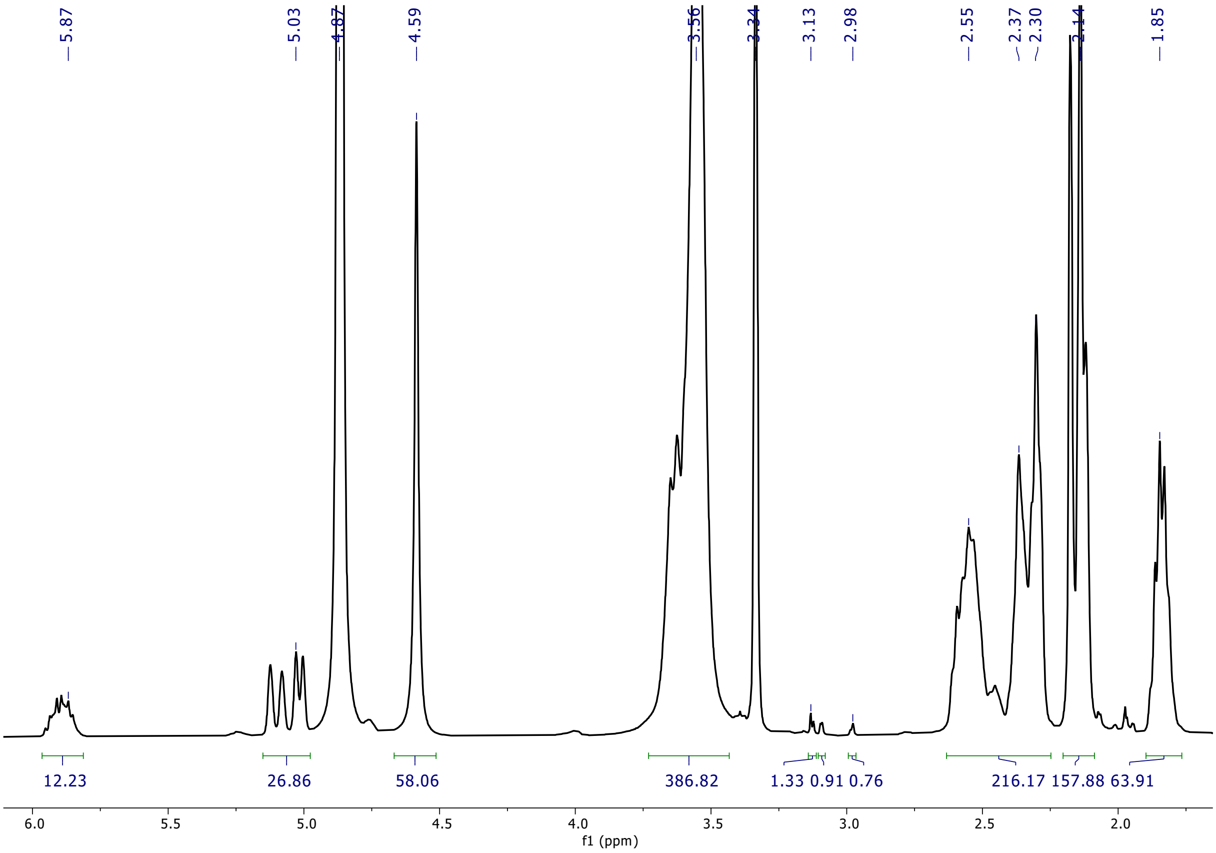

Figure S10: ^{1}H NMR (400 MHz, MeOD-4) spectrum of PMeOx$_{52}$-PPentynOx$_{32}$-PButenOx$_{13}$.

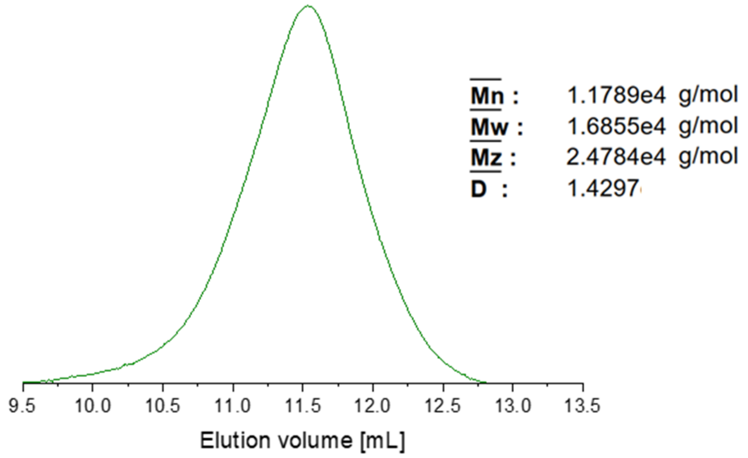

Figure S11: GPC RI trace (eluent: NMP) of PMeOx$_{52}$-PPentynOx$_{32}$-PButenOx$_{13}$.

P4: PMeOx$_{57}$-PButenOx$_8$

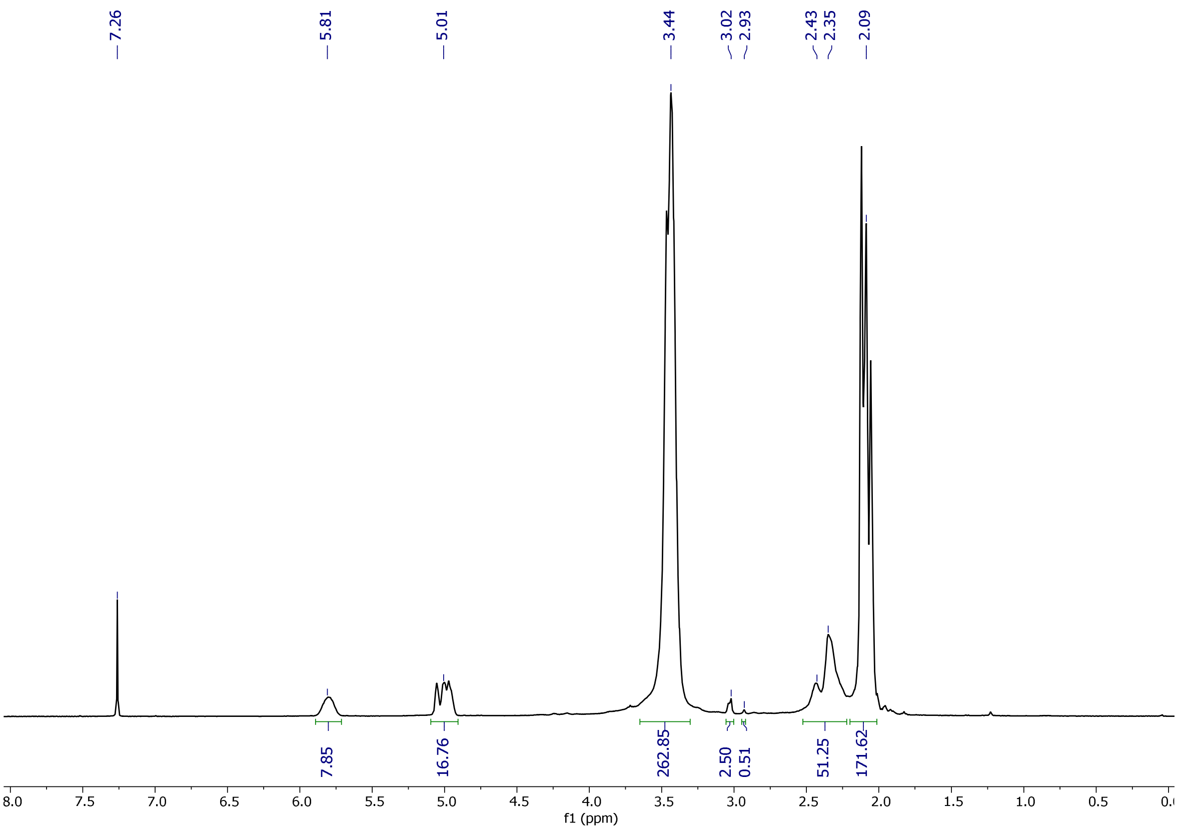

Figure S12: ^{1}H NMR (400 MHz, $CDCl_3$) spectrum of PMeOx$_{57}$-PButenOx$_8$.

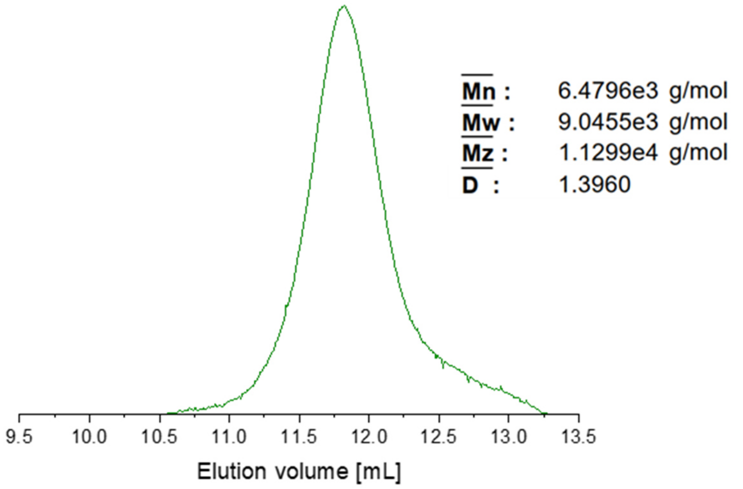

Figure S13: GPC RI trace (eluent: NMP) of PMeOx$_{57}$-PButenOx$_8$.

4 Synthesis of azide and thiol molecules with specific functional groups

4.1 Synthesis of azide molecules

The following azide molecules such as 6-azido-hexanoic acid, 6-azido-hexanol, 3-azido-propyl amine and 3-azido-propyl thiourea Rhodamine B were synthesized from the starting materials 6-bromo-hexanoic acid, 6-bromo-hexanol, 3-bromo-propyl amine and Rhodamine B thioisocyanate, according to a synthetic protocol as described by Grandjean *et al.*[4]

4.1.1 Synthesis of 6-azido-hexanoic acid (COOH-N_3) [4]

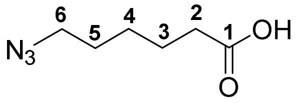

6-Bromohexanoic acid (1 eq., 12.5 g, 64.2 mmol) and sodium azide (2 eq., 8.33 g, 128.4 mmol) were dissolved in 60 mL of dry DMF under nitrogen atmosphere. The reaction mixture was stirred for 4.5 h at 85 °C and then for 15 h at room temperature. Then, the reaction mixture was diluted and washed four times (ca. 150 mL) with diethyl ether and dried over sodium sulfate. The solvent was removed *in vacuo* and a colorless viscous liquid was obtained without further purification. (9.26 g, 59.0 mmol, 92%). **^{1}H NMR** (400 MHz, $CDCl_3$): δ (ppm) = 10.58 (m, 0.92H, COO*H*), 3.25 (m, 2H, H^6), 2.32 (m, 2H, H^2), 1.67-1.57 (m, 4H, H^{3+5}), 1.39 (m, 2H, H^4). **^{13}C NMR** (100 MHz, $CDCl_3$): δ (ppm) = 179.2 (C^1), 51.2 (C^6), 33.9 (C^2), 28.5 (C^5), 26.2 (C^4), 24.1 (C^3). **ESI-MS:** m/z calc: 157.09, found: 157.09 + H^+.

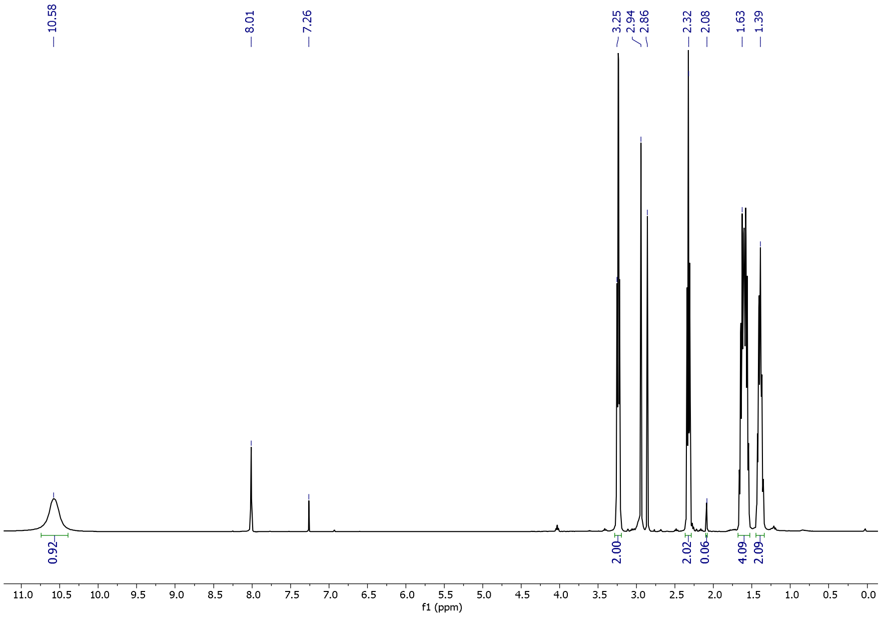

Figure S14: ^{1}H NMR (400 MHz, $CDCl_3$) spectrum of 6-azido-hexanoic acid.

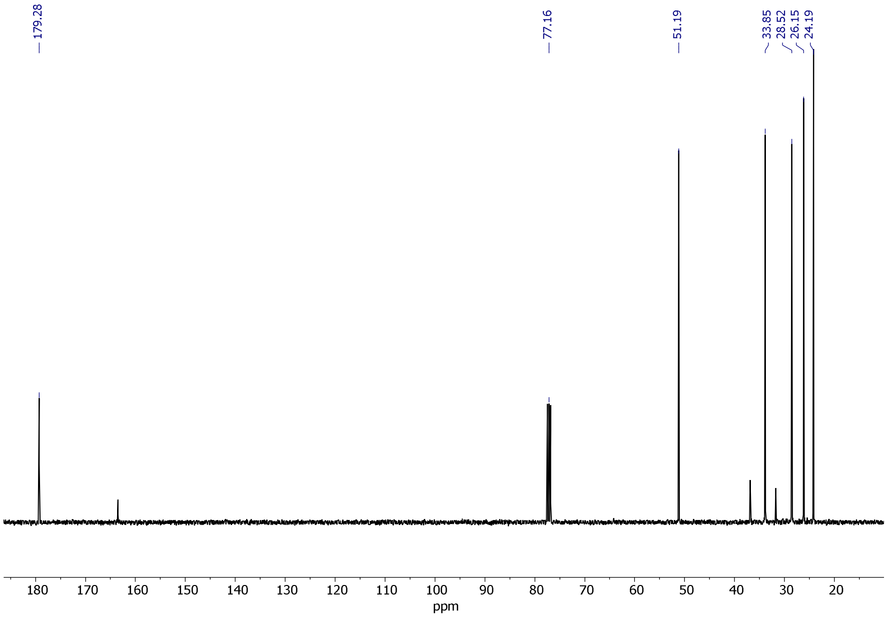

Figure S15: ^{13}C NMR (100 MHz, $CDCl_3$) spectrum of 6-azido-hexanoic acid.

4.1.2 Synthesis of 6-azido-hexanol (OH-N_3) [4]

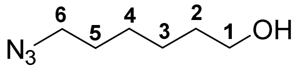

6-Bromohexanol (5.0 g, 27.7 mmol) and sodium azide (3.6 g, 55.3 mmol) were dissolved in 30 mL of dry DMF under nitrogen atmosphere. The reaction was executed at a temperature of 85 °C for 4.5 h. The composite was further stirred at room temperature for 15 h. Furthermore, the reaction mixture was diluted and washed four times (ca. 150 mL) with diethyl ether and dried over sodium sulfate. The solvent was removed *in vacuo* and a colorless viscous liquid was obtained without further purification. (3.47 g, 24.3 mmol, 88%). **^{1}H NMR** (400 MHz, $CDCl_3$): δ (ppm) = 3.64 (t, 2H, H^1), 3.26 (m, 2H, H^6), 1.67-1.57 (m, 4H, H^{2+5}), 1.40 (m, 4H, H^{3+4}). **^{13}C NMR** (100 MHz, $CDCl_3$): δ (ppm) = 62.8 (C^1), 51.5 (C^6), 32.7 (C^2), 28.9 (C^5), 26.6 (C^4), 25.5 (C^3).

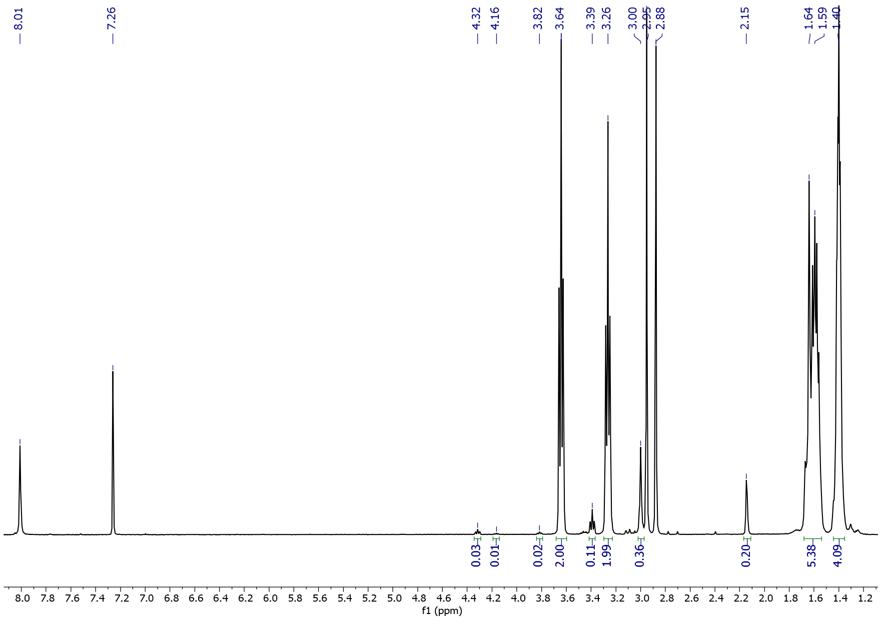

Figure S16: ^{1}H NMR (400 MHz, $CDCl_3$) spectrum of 6-azido-hexanol.

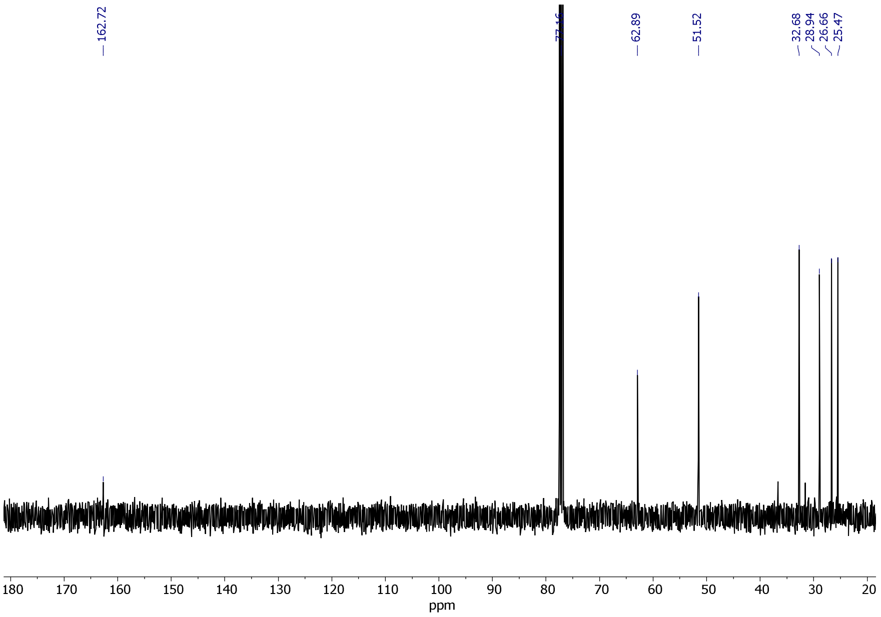

Figure S17: ^{13}C NMR (100 MHz, $CDCl_3$) spectrum of 6-azido-hexanol.

4.1.3 Synthesis of 3-azido-propyl amine (Amine-N_3) [4]

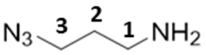

3-Bromopropylamine (2.0 g, 9.14 mmol) was dissolved in 5 mL of deionized water. Sodium azide (1.77 g, 27.22 mmol) was added and the reaction was heated up to 80 °C for 20 h under reflux conditions and vigorous stirring. The reaction mixture was cooled with an ice bath and potassium hydroxide (1.32 g, 23.61 mmol) was slowly added to the mixture. The product was extracted four times with diethylether and the organic phase was dried over sodium sulfate. The organic phase was concentrated using a rotary evaporator and dried under reduced pressure. **^{1}H NMR** (400 MHz, $CDCl_3$): δ (ppm) = 3.37 (t, 2H, H^1), 2.81 (t, 2H, H^3), 1.73 (p, 2H, H^2), 1.40 (s, 2H, $N\underline{H}_2$). **^{13}C NMR** (100 MHz, $CDCl_3$): δ (ppm) = 49.1 (C^3), 39.3 (C^3), 32.3 (C^1).

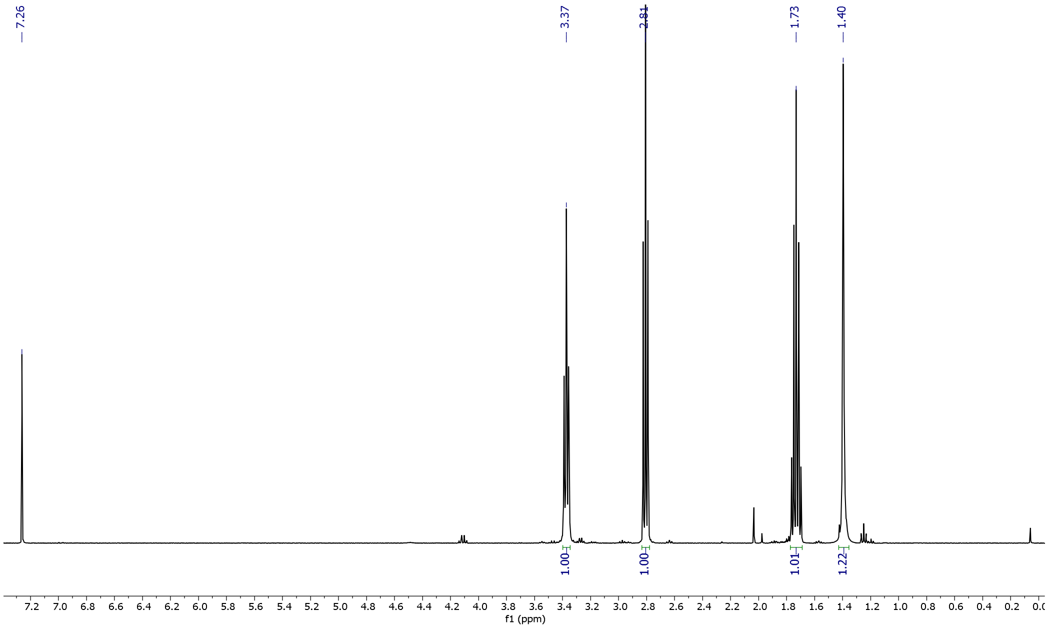

Figure S18. ^{1}H NMR (400 MHz, $CDCl_3$) spectrum of 3-azido-propyl amine.

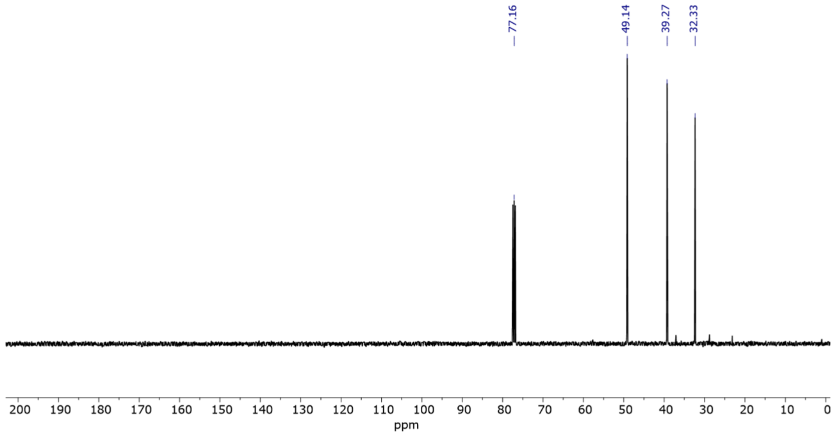

Figure S19. ^{13}C NMR (100 MHz, $CDCl_3$) spectrum of 3-azido-propyl amine.

4.1.4 Synthesis of 1-(3-azidopropyl)-3-RhodamineB thiourea (RhoB-N_3) [5]

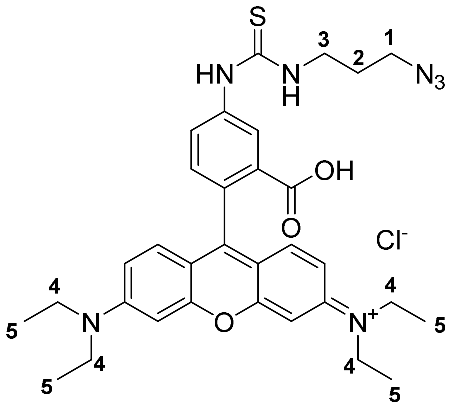

Rhodamine B isothiocyanate (31 mg, 0.06 mmol) and triethyl amine (0.011 mg, 0.11 mmol) were dissolved in DCM (100 mL) under N_2-atmosphere. The solution was cooled down to 0 °C and a solution of 3-azidopropylamine (0.11 mg, 0.11 mmol) in DCM (10 mL) was added under constant stirring. The reaction mixture was stirred at room temperature overnight. A shift in color from pink to a deep red is noticeable. The solvent was removed and the residue was again dissolved in MeOH (50 mL). The MeOH phase was extracted with *n*-hexane (8x20 mL) to remove the amine and the combined hexane phases were extracted with MeOH until colorless (5x10 mL). The evaporation of the solvent yielded a red powder (28 mg, 44 µmol, 73%). **^{1}H NMR** (400 MHz, CD_3OD): δ (ppm) = 8.30-6.25 (m, 9H, $H^{aromatic}$), 3.65 (m, 2H, H^4), 3.50 (t, 2H, H^3), 3.01 (t, 2H, H^1), 1.90 (m, 2H, H^2), 1.30 (s, 12H, H^5).

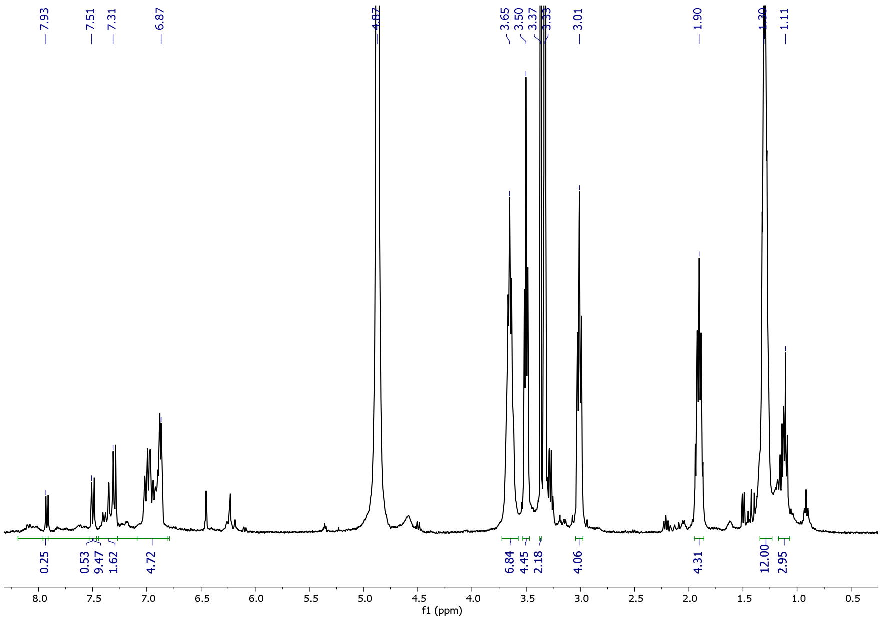

Figure S20. ^{1}H NMR (400 MHz, $CDCl_3$) spectrum of RhoB-N_3.

4.2 Synthesis of thiol molecules

Thiol molecules with incorporated specific functional groups (FG) such as catechols and sulfur have been synthesized as described in the following, whereas thiol compounds of carboxylates (3-mercapto propionic acid) and alkoxysilanes ((3 mercaptopropyl)trimethoxysilane) were commercially available.

4.2.1 Synthesis of (*N*-(3,4-dihydroxyphenethyl)-4-mercaptobutanimidamide) (Cat-SH)

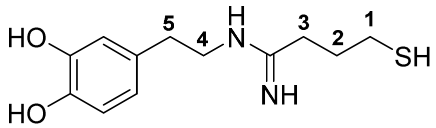

The thiol-catechol molecule was synthesized by converting the amine group of dopamine into a thiol function using 2-iminothiolane (Traut's reagent) at 70°C and pH 9. The mercapto-catechol (*N*-(3,4-dihydroxyphenethyl)-4-mercapto-butanimidamide) was extracted with chloroform and obtained as a yellowish solid. NMR spectral analysis revealed the successful conversion and the presence of thio-functionalized catechol (Figure **S22**), as identified according to the paper of Rohrer *et al.* [6] The additional NMR signals at 1.87, 2.30 and 2.49 ppm point towards the presence of a second species, which might be the di-substituted byproduct (Figure **S22**). This is presumably caused by the reaction of the catechol-OH with the Traut's reagent. The di-substitution is supported by ^{13}C NMR analysis (Figure S23) based on the carbon signals between 175 and 180 ppm suggesting the presence of aromatic amide bonds. Nevertheless, NMR compositional analysis suggests the weight content of the targeted mercapto-catechol of 66%. ESI-MS analysis confirms the co-presence of the desired synthesis product N-(3,4-dihydroxyphenethyl)-4-mercaptobutanimidamide (m/z = 254.3 + Na^+) and the di-substituted product (m/z = 355.5 + Na^+) (Figure S24).

<u>Experimental procedure:</u>

Dopamine hydrochloride (0.15 g, 0.79 mmol) was dissolved in deionized water and degassed with nitrogen. The pH-value was set to 8-9 by addition of sodium carbonate. 2-Iminothiolane hydrochloride (Traut's reagent, 0.033 mg, 0.24 mmol) was added and the reaction mixture was heated to 70 °C for 15 h under constant stirring. The water phase was extracted with $CHCl_3$ at pH 8-9 and the organic phase was dried in vacuum. A yellowish solid was obtained (34 mg, 134 mmol, 56%). **^{1}H NMR** (400 MHz, CD_3OD): δ (ppm) = 6.71-6.55 (m, 3H, $H^{aromatic}$), 3.42 (m, 2H, H^3), 3.29 (t, 2H, H^1), 2.81 (t, 2H, H^5), 2.69 (m, 2H, H^4), 2.15 (s, 12H, H^2).

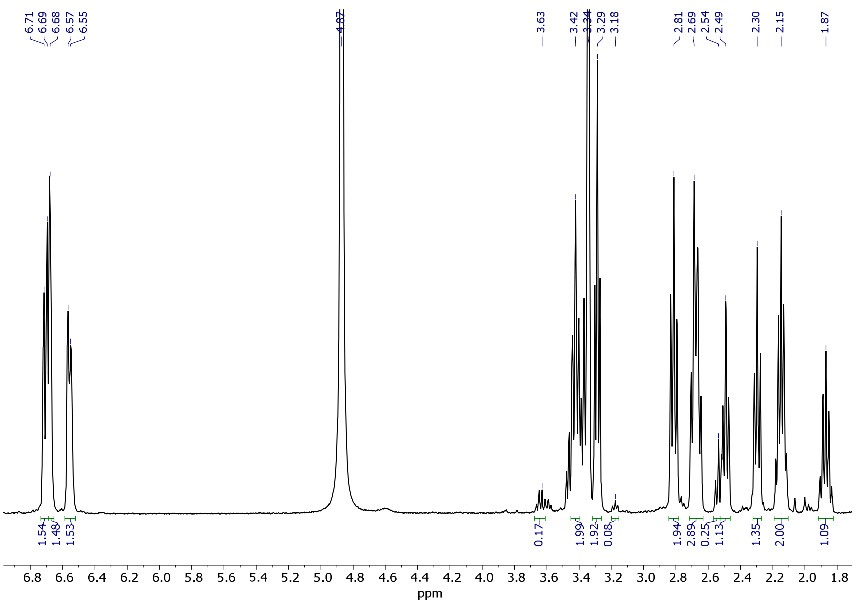

Figure S21. ^{1}H NMR (400 MHz, CD_3OD) spectrum of *N*-(3,4-dihydroxyphenethyl)-4-mercaptobutanimidamide.

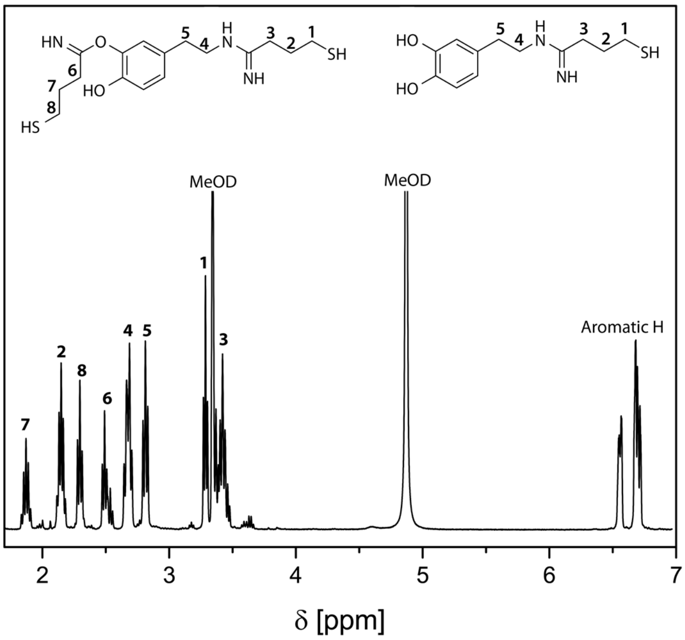

Figure S22. ^{1}H NMR (400 MHz, CD_3OD) spectrum of N-(3,4-dihydroxyphenethyl)-4-mercaptobutanimidamide, as identified according to the publication of Rohrer et al.[6] The additional NMR signals at 1.87, 2.30 and 2.49 ppm point towards the presence of a second species, which might be the disubstituted byproduct (Figure 6.8). NMR compositional analysis suggests the weight content of the targeted mercapto-catechol of 66%.

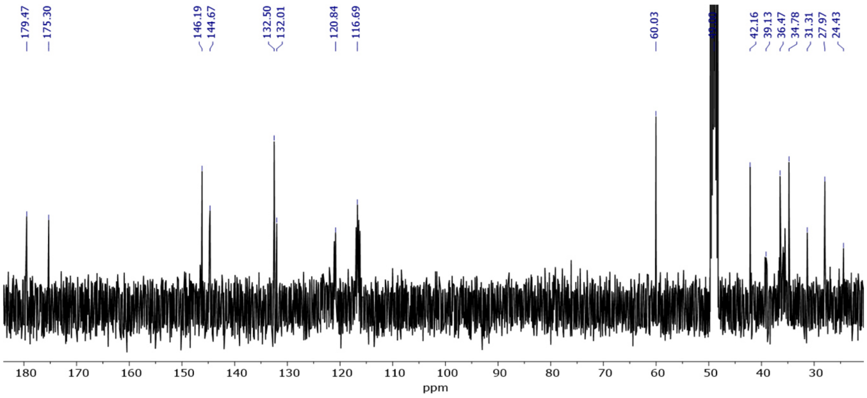

Figure S23. ^{13}C NMR (100 MHz, CD_3OD) spectrum of N-(3,4-dihydroxyphenethyl)-4-mercaptobutanimidamide.

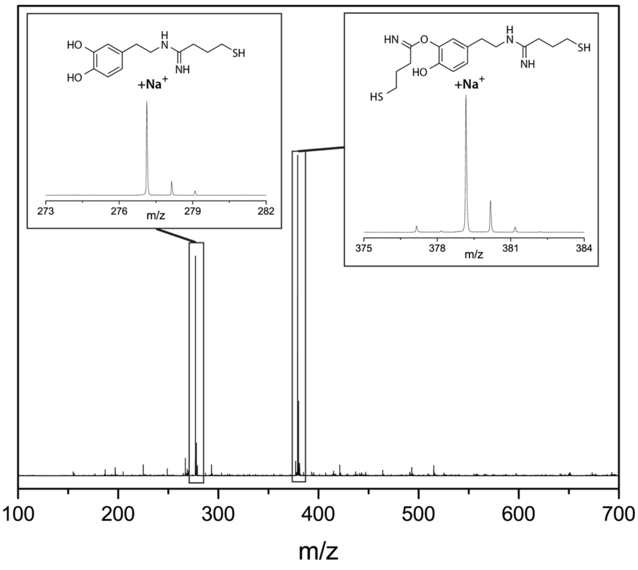

Figure S24: ESI-MS spectrum of N-(3,4-dihydroxyphenethyl)-4-mercaptobutanimidamide.

4.2.2 Synthesis of *S*-(8-mercaptooctyl)ethanethioate (SAc-SH) [7]

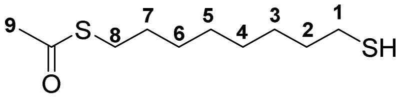

The thioester containing molecule was synthesized by unilateral acetylation of 1,8-dimercaptooctane by microwave-assisted synthesis at 160 °C in the presence of acetic acid anhydride coated silica gel to obtain *S*-(8-mercaptooctyl)ethanethioate. [7] Hereby, increased microwave reaction time was found to increase the degree of acetylation (5 min: 2%; 20 min: 14% and 60 min: 63%) according to NMR analysis. The final liquid product was synthesized under microwave irradiation for 60 min and analyzed by ^{1}H NMR spectroscopy (Figure S25 - Figure S27) suggesting the mixture of 73% monoacetylated and 27% diacetylated thioesters. A similar ratio of two species was determined by GC (Figure S27). The liquid product mixture was not further purified, since the diacetylated thioester does not affect the 2.PPM and dialysis purification removes the side product.

<u>Experimental procedure:</u>

1,8-Octanedithiol (0.4 g, 2.24 mmol) and silica gel (630 mg) were suspended in acetic acid anhydride (630 mg, 6.38 mmol) in a microwave reactor (10 mL) and shaken. The reaction was performed with microwave irradiation for 60 min at 160 °C without stirring. After cooling down to room temperature, the yellowish dispersion was diluted with diethyl ether, filtrated and the solvents were removed under reduced pressure. A yellowish liquid was obtained. **^{1}H NMR** (400 MHz, $CDCl_3$): δ (ppm) = 2.85 (t, 2H, H^{8}), 2.51 (t, 2H, H^{1}), 2.32 (t, 3H, H^{9}), 1.70-1.50 (m, 4H, H^{2+7}), 1.45-1.25 (m, 8H, H^{3-6}). 1,8-octanediethanethioate: δ (ppm) = 2.85 (t, 1.44H, H^{8}), 2.32 (t, 2.13H, H^{9}), 1.70-1.50 (m, 1.5H, H^{2+7}), 1.45-1.25 (m, 4H, H^{3-6}). **^{13}C NMR** (100MHz, $CDCl_3$): δ (ppm) = 196.2 (C^{SAc}), 34.1 (C^{2}), 30.8 (C^{9}), 29.3-28.4 (C^{3-8}), 24.8 (C^{1}). **GC:** retention time: 4.62 min (60.4%), 6.96 min (26.6%).

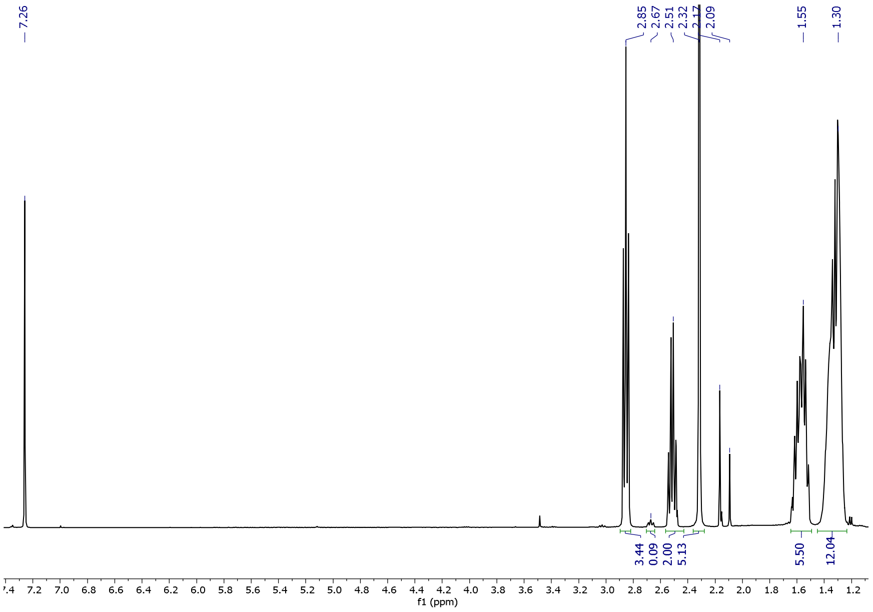

Figure S25. Proton NMR spectrum (400 MHz, $CDCl_3$) of *S*-(8-mercaptooctyl)ethanethioate.

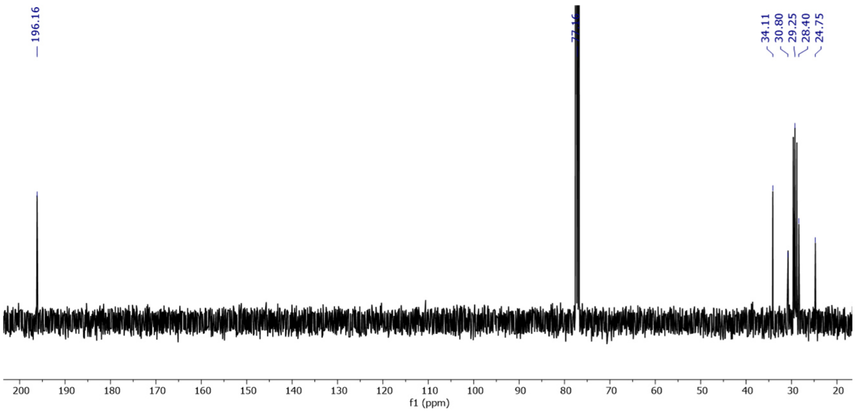

Figure S26. Carbon NMR spectrum (400 MHz, $CDCl_3$) of S-(8-mercaptooctyl) ethanethioate.

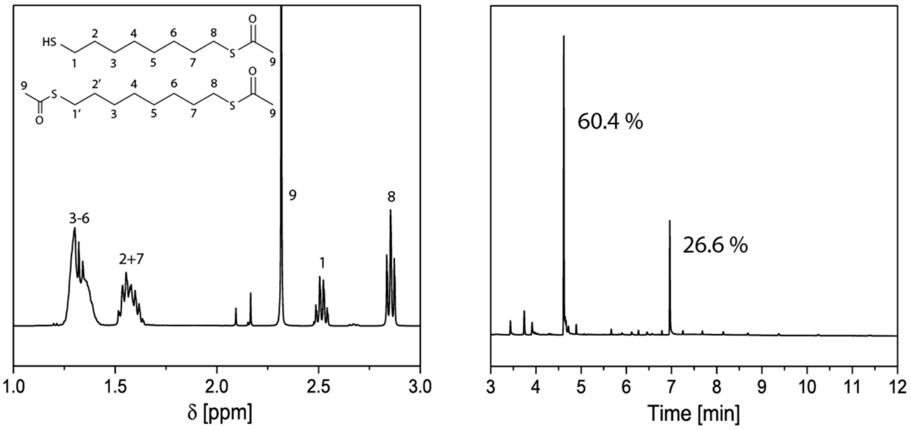

Figure S27. ^{1}H NMR (400 MHz, $CDCl_3$) spectrum (left) and gas chromatogram (right) of crude acetylated 1,8-dimercaptooctane.

5 Post-polymerization modification (PPM)

The modular toolkit comprises multifunctional block copolymers based on poly(2-oxazoline) with separated segments of numerous pendent chemical functionalities. The functional groups were attached to the PMeOx-PPentynOx-PButenOx block copolymers via orthogonal post-polymerization modification (Table S1). The experimental details and analytical data are provided below (section 5.1 and 5.2).

Table S1. Overview of multifunctional block copolymers PMeOx-PPentynOx(FG)-PButenOx$_{11}$(BG) with various functional groups (FG) and binding groups (BG).

Functionalized polymers [a]	Backbone [b]	PPM[c]	Component[d]	Page
**watersoluble – negative charges – binding groups**				
PMeOx$_{43}$ - PPentynOx$_{11}$(COOH)$_{11}$ - PButenOx$_{11}$	**P1**	1	N_3-$(CH_2)_5$COOH	*24*
PMeOx$_{43}$ - PPentynOx$_{11}$(COOH)$_{11}$ - PButenOx$_{11}$(SiOMe)$_6$	**P1**	2	thiol-siloxane	*35*
PMeOx$_{52}$ - PPentynOx$_{32}$(COOH)$_{26}$ - PButenOx$_{13}$	**P3**	1	N_3-$(CH_2)_5$COOH	*25*
PMeOx$_{52}$ - PPentynOx$_{32}$(COOH)$_{26}$ - PButenOx$_{13}$(SAc)$_{13}$	**P3**	2	thiol-thioester	*33*
**watersoluble – hydroxyl groups – binding groups**				
PMeOx$_{52}$ - PPentynOx$_{32}$(OH)$_{32}$ - PButenOx$_{13}$	**P3**	1	N_3-$(CH_2)_6$OH	*26*
PMeOx$_{52}$ - PPentynOx$_{32}$(OH)$_{32}$ - PButenOx$_{13}$(COOH)$_{13}$	**P3**	2	COOH-SH	*30*
PMeOx$_{52}$ - PPentynOx$_{32}$(OH)$_{32}$ - PButenOx$_{13}$(Cat)$_2$	**P3**	2	Cat-SH	*31*
PMeOx$_{52}$ - PPentynOx$_{32}$(OH)$_{32}$ - PButenOx$_{13}$(SAc)$_{12}$	**P3**	2	SAc-SH	*32*
PMeOx$_{52}$ - PPentynOx$_{32}$(OH)$_{32}$ - PButenOx$_{13}$(SiOMe)$_x$	**P3**	2	thiol-siloxane	*34*
**watersoluble – positive charges – binding groups**				
PMeOx$_{50}$ - PPentynOx$_9$(NH$_2$)$_7$ - PButenOx$_9$	**P2**	1	N_3-$(CH_2)_3$-NH_2	*28*
**1.PPM polymers**				
PMeOx$_{52}$ - PPentynOx$_{32}$(RhoB)$_{1.5}$ - PButenOx$_{13}$	**P3**	1	N_3-RhoB	*29*
PMeOx$_{52}$ - PPentynOx$_{32}$(NH$_2$)$_x$ - PButenOx$_{13}$	**P3**	1	N_3-$(CH_2)_3NH_2$	*27*
PMeOx$_{50}$ - PPentynOx$_9$(Aryl) - PButenOx$_9$	**P2**	1	4-iodobenzotrifluoride	*36*
PMeOx$_{57}$ - PButenOx$_8$(Benzoate)	**P4**	1	methyl-4-iodobenzoate	*37*

[a] ^{1}H-NMR compositional analysis. [b] The synthesis and the exact composition is provided in chapter 3 [c] Number of orthogonal post-polymerization modification (PPM). [d] The azide and thiol molecules were synthesized as described in chapter 4, such as N_3-$(CH_2)_5$OH (6-azido-hexanol), N_3-$(CH_2)_5$COOH (6-azido-hexanoic acid), N_3-$(CH_2)_3$-NH_2 (3-azido-propyl amine), N_3-RhoB (1-(3-azidopropyl)-3-Rhodamine B thiourea), SAc-SH (*S*-(8-mercaptooctyl)ethanethioate), Cat-SH ((*N*-(3,4-dihydroxyphenethyl)-4-mercaptobutanimid-amide)), or purchased from common suppliers such as thiol-acid (3-mercaptopropionic acid), thiol-silane ((3-mercaptopropyl)trimethoxysilane), 4-iodobenzotrifluoride and methyl-4-iodobenzoate.

5.1 1. PPM: Copper-catalyzed alkyne-azide cycloaddition

General procedure. The alkyne bearing poly(2-oxazoline) and the azide molecule (2 equiv. per alkyne group) were dissolved 20 mL of a water/*tert*-butanol mixture (1:1 v/v). After adding copper(II)sulfate (0.02 eq. per alkyne group) and sodium ascorbate (0.2 eq. per alkyne group), the solution was stirred for 2 d at room temperature. The reaction mixture was dialyzed stepwise against methanol, aqueous EDTA solution and water (MWCO = 1000 g/mol) for 2 d each. Freeze-drying yielded the polymer powders.

5.1.1 COOH-modification

The carboxylate-modification was performed according to the general procedure of alkyne-azide click reaction as described above (Section 5.1, page 24).

P1-COOH = $PMeOx_{43}$-$PPentynOx_{11}(COOH)_{11}$-$PButenOx_{11}$

$PMeOx_{43}$-$PPentynOx_{11}$-$PButenOx_{11}$ (0.147 mmol, 1.0 g), 6-azido-hexanoic acid (3.23 mmol, 508 mg). Yield 1.43 g. **^{1}H NMR** (400 MHz, CD_3OD): δ (ppm) = 7.79 (m, 11H, H^{11}), 5.86 (m, 12H, H^6), 5.05 (m, 26H, H^7), 4.35 (m, 23H, H^{12}), 3.53 (m, 274H, H^{2-3}), 2.72 (m, 26H, H^8), 2.54-2.24 (m, 84H, $H^{4,5,10,16}$), 2.13 (m, 138H, H^1), 1.90 (m, 48H, $H^{9,13}$), 1.61 (m, 23H, H^{15}), 1.33 (m, 23H, H^{14}). **ATR-IR**: 1722 cm^{-1} (COOH), 2880-3000 cm^{-1} (1,2,3-triazole and methylene vibrations).

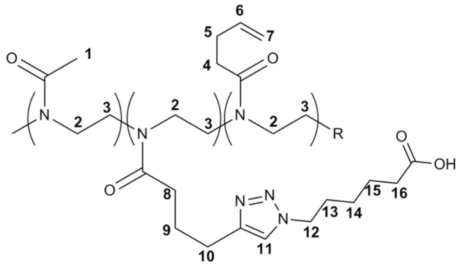

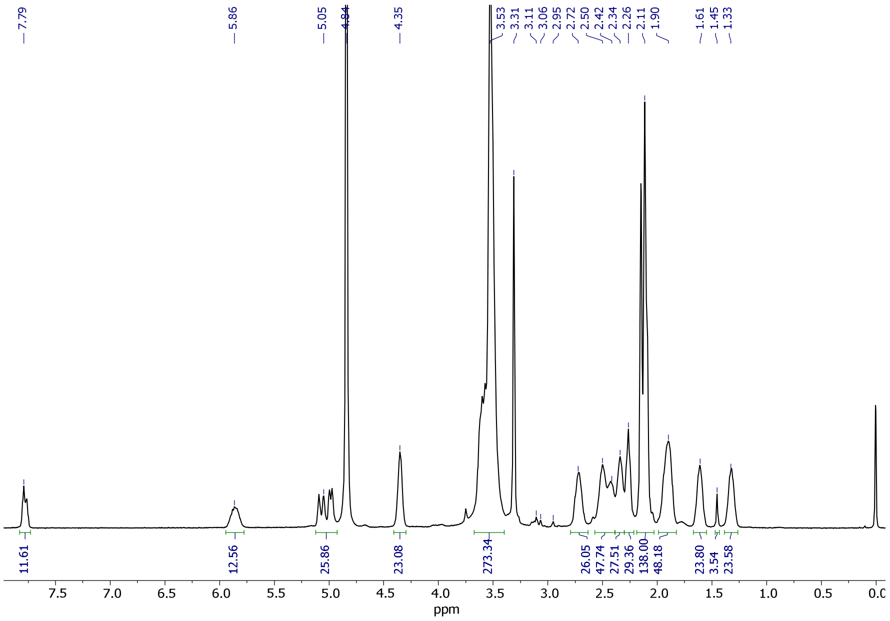

Figure S28. ^{1}H NMR (400 MHz, CD_3OD) spectrum of $PMeOx_{43}$-$PPentynOx_{11}(COOH)_{11}$-$PButenOx_{11}$.

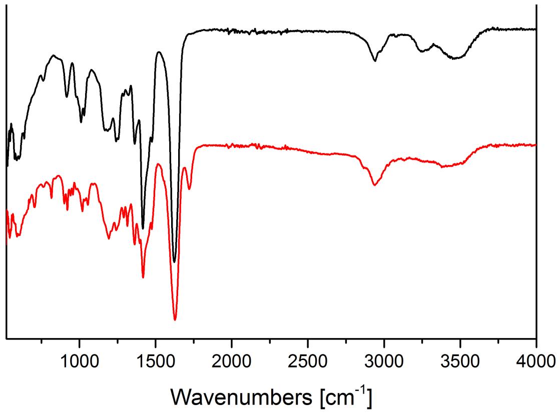

Figure S29. ATR-IR spectra of $PMeOx_{43}$-$PPentynOx_{11}$-$PButenOx_{11}$ (black line) and the corresponding carboxylate-functionalized $PMeOx_{43}$-$PPentynOx_{11}(COOH)_{11}$-$PButenOx_{11}$ (red line).

P3-COOH = $PMeOx_{52}$-$PPentynOx_{32}(COOH)_{26}$-$PButenOx_{13}$

$PMeOx_{52}$-$PPentynOx_{32}$-$PButenOx_{13}$ (57 µmol, 0.593 g), 6-azido-hexanoic acid (3.88 mmol, 609 mg). Yield 1.18 g. **^{1}H NMR** (400 MHz, CD_3OD): δ (ppm) = 7.82 (m, 25H, H^{11}), 5.88 (m, 12H, H^6), 5.09 (m, 26H, H^7), 4.37 (m, 50H, H^{12}), 3.55 (m, 344H, $H^{2\text{-}3}$), 2.74 (m, 56.2H, H^8), 2.54-2.24 (m, 150H, $H^{4,5,10,16}$), 2.15 (m, 154H, H^1), 1.91 (m, 108H, $H^{9,13}$), 1.63 (m, 66H, H^{15}), 1.34 (m, 62H, H^{14}). **ATR-IR**: 1722 cm^{-1} (COOH), 2880-3000 cm^{-1} (1,2,3-triazole and methylene vibrations).

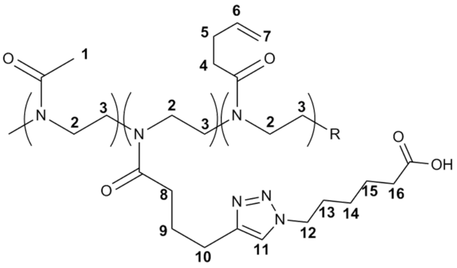

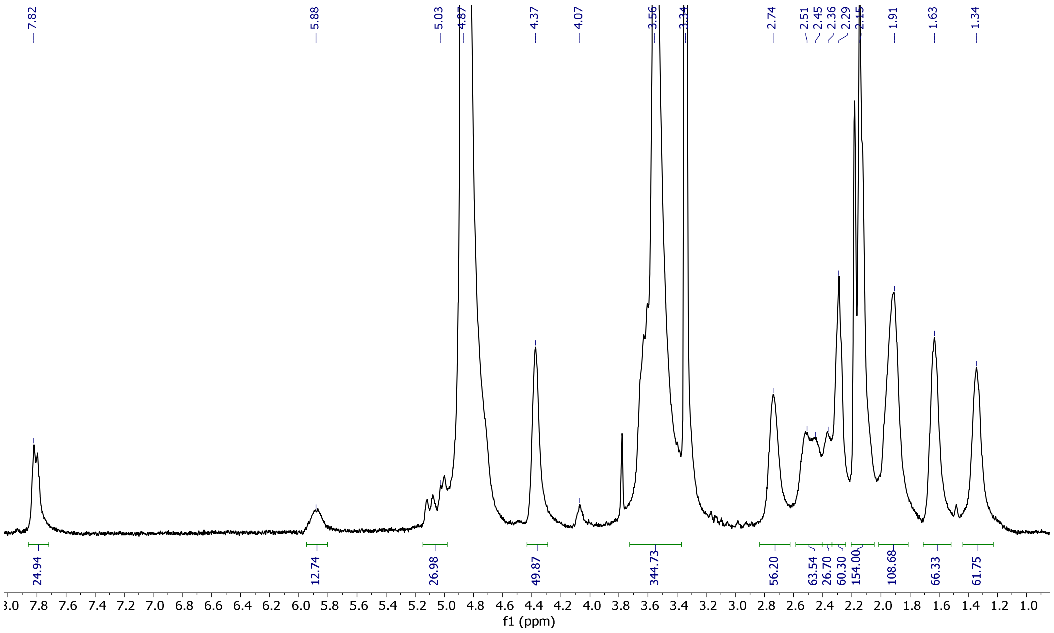

Figure S30. ^{1}H NMR (400 MHz, CD_3OD) spectrum of $PMeOx_{52}$-$PPentynOx_{32}(COOH)_{26}$-$PButenOx_{13}$.

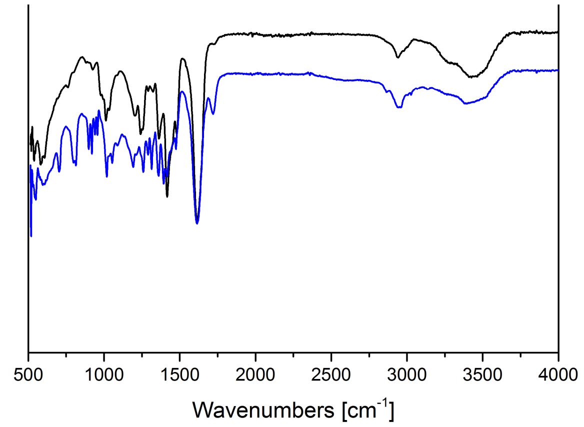

Figure S31. ATR-IR spectra of $PMeOx_{52}$-$PPentynOx_{32}$-$PButenOx_{13}$ (black line) and the corresponding carboxylate-functionalized $PMeOx_{52}$-$PPentynOx_{32}(COOH)_{26}$-$PButenOx_{13}$ (blue line).

5.1.2 OH-modification

The hydroxyl-modification was performed according to the general procedure of alkyne-azide click reaction as described above (Section 5.1, page 24).

P3-OH = $PMeOx_{52}$-$PPentynOx_{32}(OH)_{32}$-$PButenOx_{13}$

$PMeOx_{51}$-$PPentynOx_{34}$-$PButenOx_{12}$ (47.8 µmol, 0.5 g), 6-azido-hexanol (3.25 mmol, 466 mg). Yield 0.56 g. **^{1}H NMR** (400 MHz, CD_3OD): δ (ppm) = 7.83 (m, 31H, H^{11}), 5.90 (m, 13H, H^{6}), 5.09 (m, 27H, H^{7}), 4.38 (m, 62.8H, H^{12}), 3.55 (m, 440H, H^{2-3}), 2.75 (m, 62.5H, H^{8}), 2.54-2.32 (m, 118H, $H^{4,5,10}$), 2.14 (m, 154H, H^{1}), 1.91 (m, 128H, $H^{9,13}$), 1.54 (m, 69H, H^{16}), 1.48-1.28 (m, 134H, $H^{14,15}$). **ATR-IR**: 2880-3000 cm^{-1} (1,2,3-triazole and methylene vibrations).

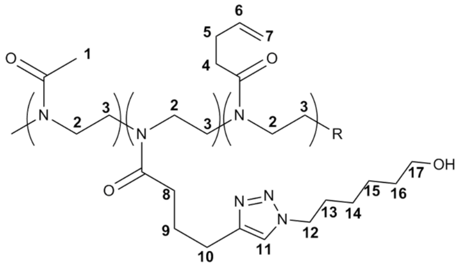

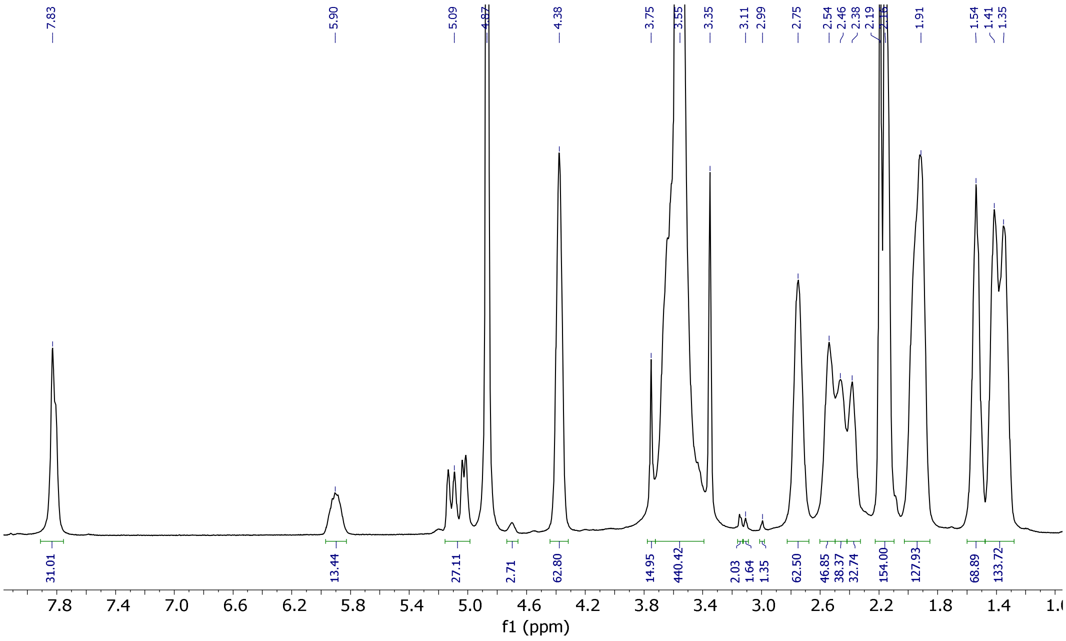

Figure S32. ^{1}H NMR (400 MHz, CD_3OD) spectrum of $PMeOx_{52}$-$PPentynOx_{32}(OH)_{32}$-$PButenOx_{13}$.

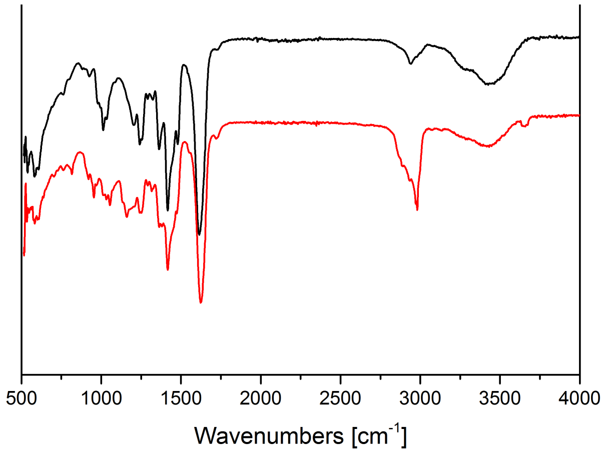

Figure S33. ATR-IR spectra of $PMeOx_{52}$-$PPentynOx_{32}$-$PButenOx_{13}$ (black line) and the corresponding hydroxyl-functionalized $PMeOx_{52}$-$PPentynOx_{32}(OH)_{32}$-$PButenOx_{13}$ (red line).

5.1.3 NH_2-modification

The amine-modification was performed according to the general procedure of alkyne-azide click reaction as described above (Section 5.1, page 24). In the case of the modification of $PMeOx_{52}$-$PPentynOx_{32}$-$PButenOx_{13}$ (**P3**) with azido propyl amine, the incorporation of the FG was qualitatively confirmed by IR analysis (Figure S35). However, as the amine-modified product was hardly soluble in common solvents, supposedly due to the polycationic block impeding the stability in solution, thus making the quantification of the implemented amine functionality impossible (Figure S34). The modification with azido propyl amine was therefore repeated with $PMeOx_{50}$-$PPentynOx_{9}$-$PButenOx_{9}$ (**P2**) having a shorter alkyne block. In this case, 78% of the alkyne groups were converted to yield the polymer structure $PMeOx_{50}$-$PPentynOx_{9}(NH_2)_7$-$PButenOx_{9}$.

P3-NH_2= $PMeOx_{52}$-$PPentynOx_{32}(NH_2)_x$-$PButenOx_{13}$

$PMeOx_{52}$-$PPentynOx_{32}$-$PButenOx_{13}$ (25 µmol, 0.266 g), 6-azido-propyl amine (1.7 mmol, 170 mg). Yield 0.28 g. **^{1}H NMR**: insoluble in common deuterated solvents. **ATR-IR**: 800 cm^{-1} (ν(N-H) out-of-plane), 2880-3000 cm^{-1} (1,2,3-triazole vibrational bands and methylene vibration), 3240 and 3440 cm^{-1} ($ν_{N-H}$ stretch vibration).

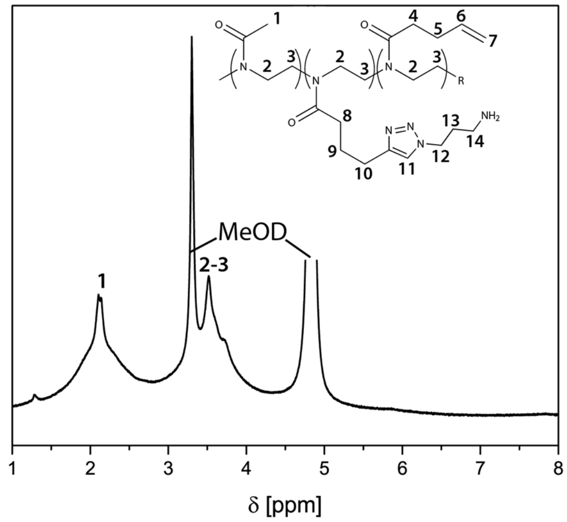

Figure S34. ^{1}H NMR (400 MHz, CD_3OD) spectrum of $PMeOx_{52}$-$PPentynOx_{32}(NH_2)_x$-$PButenOx_{13}$.

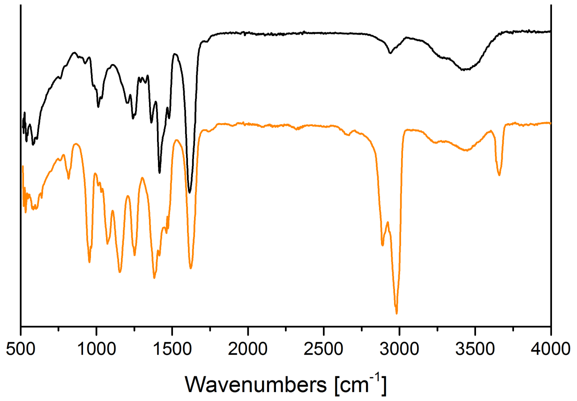

Figure S35. ATR-IR spectra of $PMeOx_{52}$-$PPentynOx_{32}$-$PButenOx_{13}$ (black line) and the corresponding amine-functionalized $PMeOx_{52}$-$PPentynOx_{32}(NH_2)_x$-$PButenOx_{13}$ (orange line).

P2-NH₂ = $PMeOx_{50}$-$PPentynOx_9(NH_2)_7$-$PButenOx_9$

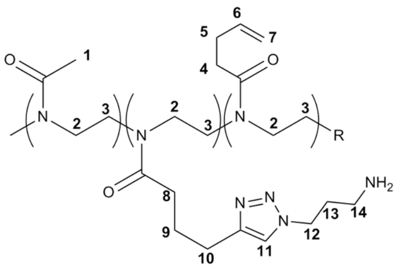

$PMeOx_{50}$-$PPentynOx_9$-$PButenOx_9$ (75 µmol, 0.5 g), 6-azido-propyl amine (1.35 mmol, 135 mg). Yield 0.61 g. **^{1}H NMR** (400 MHz, CD_3OD): δ (ppm) = 7.89 (m, 8H, H^{11}), 5.88 (m, 10H, H^6), 5.07 (m, 21H, H^7), 4.45 (m, 13.8H, H^{12}), 3.56 (m, 242H, H^{2-3}), 3.12-3.00 (m 12.3H, H^{14}), 2.87-2.60 (m, 26H, H^{10}), 2.60-2.49 (m, 19.5H, H^8), 2.49-2.42 (m, 12H, H^{13}), 2.42-2.26 (m, 33H, H^{4-5}), 2.14 (m, 150H, H^1), 1.84 (m, 21H, H^9). **ATR-IR**: 800 cm^{-1} (ν(N-H) out-of-plane), 2880-3000 cm^{-1} (1,2,3-triazole and methylene vibrations), 3240 and 3440 cm^{-1} (ν_{N-H} stretch vibration).

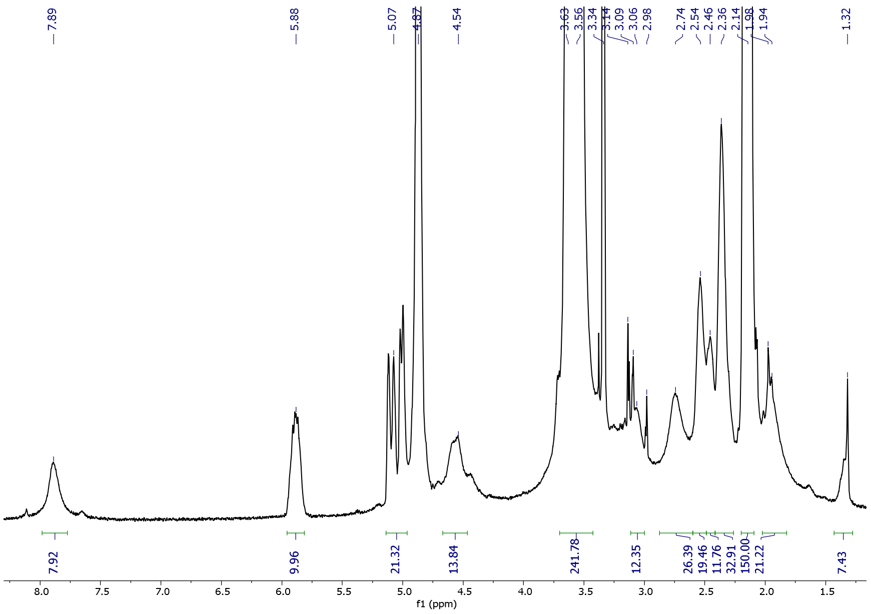

Figure S36. ^{1}H NMR (400 MHz, $CDCl_3$) spectrum of $PMeOx_{50}$-$PPentynOx_9(NH_2)_7$-$PButenOx_9$.

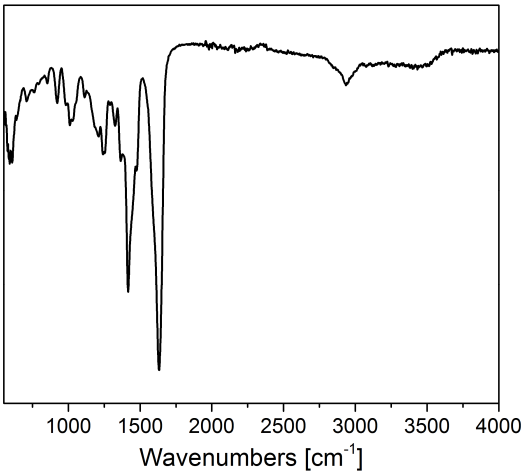

Figure S37. ATR-IR spectrum of the amine-functionalized PMeOx$_{50}$-PPentynOx$_{9}$(NH$_2$)$_7$-PButenOx$_9$.

5.1.4 RhoB-modification

The RhoB-modification was performed according to the general procedure of alkyne-azide click reaction as described above (Section 5.1, page 24). An intense pink powder was obtained with an absorption maximum at 558 nm (UV/Vis spectroscopy in methanol). Just about 4.7% of alkyne groups reacted to give a polymer with the final composition PMeOx$_{52}$-PPentynOx$_{32}$(RhoB)$_{1.5}$-PButenOx$_{13}$. This low conversion might arise from the poor water-solubility of the hydrophobic dye.

P1-RhoB = PMeOx$_{52}$-PPentynOx$_{32}$(RhoB)$_{1.5}$-PButenOx$_{13}$

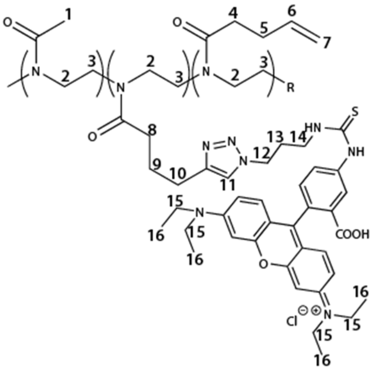

PMeOx$_{52}$-PPentynOx$_{32}$-PButenOx$_{13}$ (5 µmol, 50 mg), 1-(3-azidopropyl)-3-RhodamineB thiourea (0.04 mmol, 25 mg). Yield 0.47 mg. **^{1}H NMR** (400 MHz, CD$_3$OD): δ (ppm) = 8.30-6.25 (m, 12.5H, H^{aromatic}), 5.90 (m, 12.3H, H^6), 5.09 (m, 30H, H^7), 4.02 (m, 6H, H^{14}), 3.56 (m, 299H, H$^{2-3}$), 2.99 (m, 41H, H^{15}), 2.54 (m, 59H, H^8), 2.40-2.30 (m, 68H, H$^{4-5}$), 2.15 (m, 153H, H^1), 1.84 (m, 34H, H^{9,13}), 1.32 (m, 22H, H^{16}).

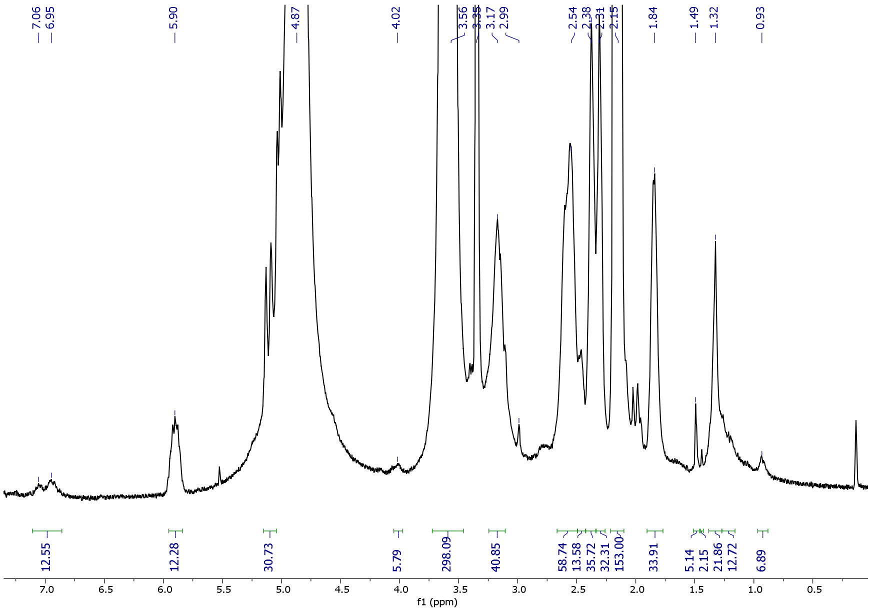

Figure S38. ^{1}H NMR (400 MHz, CDCl$_3$) spectrum of PMeOx$_{52}$-PPentynOx$_{32}$(RhoB)$_{1.5}$-PButenOx$_{13}$.

5.2 2. PPM: Thiol-ene click reaction

General procedure. The olefin bearing polyoxazoline and the thiol (2 equiv. per olefin group) was dissolved in an anhydrous methanol/tetrahydrofuran mixture (10 mL, 1:1 v/v). After the addition of 2,2-dimethoxy-2-phenylacetophenone (DMPA) (1-2 mg), the opaque solution was stirred under N_2-atmosphere for 2 d at room temperature under UV irradiation (λ = 356nm). The reaction mixture was dialyzed stepwise against methanol and water (MWCO = 1000 g/mol) for 2 d each. Freeze-drying yielded the polymer powders.

5.2.1 COOH-modification

The carboxylate-modification was performed according to the general procedure of thiol-ene click reaction as described above (Section 5.2, page 30).

P3-OH-COOH = PMeOx$_{52}$-PPentynOx$_{32}$(OH)$_{32}$-PButenOx$_{13}$(COOH)$_{13}$

PMeOx$_{52}$-PPentynOx$_{32}$(OH)$_{32}$-PButenOx$_{13}$ (4 µmol, 58 mg), 3-mercaptopropionic acid (96 µmol, 10.2 mg), DMPA (2 mg). Yield: 0.62 mg. **^{1}H NMR** (400 MHz, CD_3OD): δ (ppm) = 7.82 (m, 31.3H, H^{11}), 4.37 (m, 62.5H, H^{12}), 3.54 (m, 388H, H^{2-3}), 2.84-2.67 (m, 84H, $H^{8,18}$), 2.67-2.57 (m, 55H, $H^{7'}$), 2.57-2.36 (m, 87H, $H^{4',10,19}$), 2.18 (m, 146H, H^1), 1.90 (m, 142H, $H^{9,13}$), 1.79-1.59 (m, 58H, $H^{5',6'}$), 1.53 (m, 65H, H^{16}), 1.40-1.32 (m, 156H, $H^{14,15}$). **ATR-IR:** 1720 cm^{-1} ν(COOH carbonyl vibration).

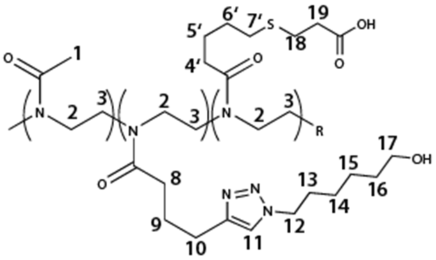

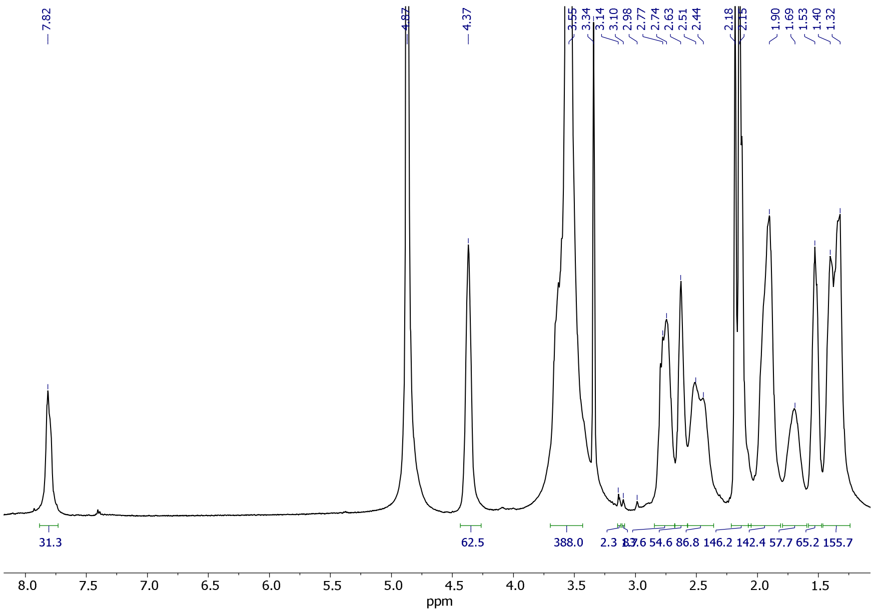

Figure S39. ^{1}H NMR (400 MHz, CD_3OD) spectrum of PMeOx$_{52}$-PPentynOx$_{32}$(OH)$_{32}$-PButenOx$_{13}$(COOH)$_{13}$.

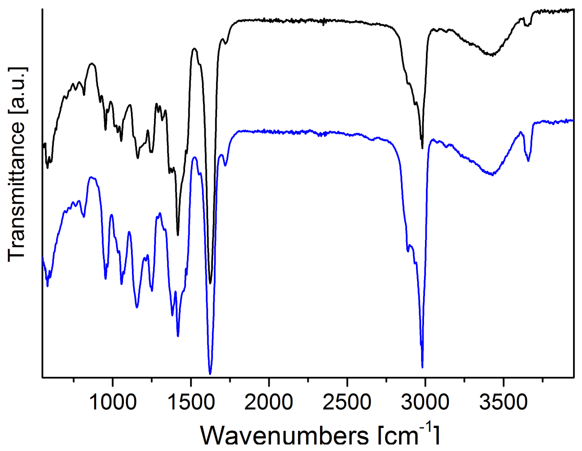

Figure S40. ATR-IR spectra of PMeOx$_{52}$-PPentynOx$_{32}$(OH)$_{32}$-PButenOx$_{13}$ (black line) and the corresponding carboxylate-functionalized PMeOx$_{52}$-PPentynOx$_{32}$(OH)$_{32}$-PButenOx$_{13}$(COOH)$_{13}$ (blue line).

5.2.2 Catechol-modification

The catechol-modification was performed according to the general procedure of thiol-ene click reaction as described above (Section 5.2, page 30).

P3-OH-Ph(OH)$_2$ = PMeOx$_{52}$-PPentynOx$_{32}$(OH)$_{32}$-PButenOx$_{13}$(Cat)$_2$

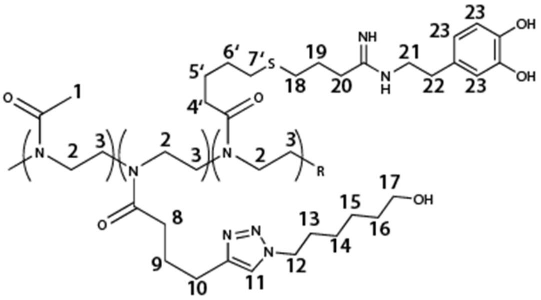

PMeOx$_{52}$-PPentynOx$_{32}$(OH)$_{32}$-PButenOx$_{13}$ (65.7 mg, 4.9 µmol), *N*-(3,4-dihydroxyphenethyl)-4-mercapto-butaneimidamide (12.4 mg, 49 µmol, 10 eq.), DMPA (1 mg). Yield 43.6 mg. **^{1}H NMR** (400 MHz, CD$_3$OD): δ (ppm) = 7.81 (m, 31H, H^{11}), 6.67-6.52 (m, 4.5H, H^{23}), 5.89 (m, 10.3H, H^{6}), 5.07 (m, 23.4H, H^{7}), 4.36 (m, 62H, H^{12}), 3.54 (m, 383H, $H^{2\text{-}3}$), 2.73 (m, 66H, $H^{8,22}$), 2.54-2.31 (m, 102H, $H^{4',4,5,7',10,18,20}$), 2.18 (m, 127H, H^{1}), 1.90 (m, 130H, $H^{9,13,19}$), 1.53 (m, 68H, $H^{5',6',16}$), 1.40-1.32 (m, 133H, $H^{14,15}$). **ATR-IR:** 1550 and 1620 cm^{-1} ν (vibration of tri-substituted benzene).

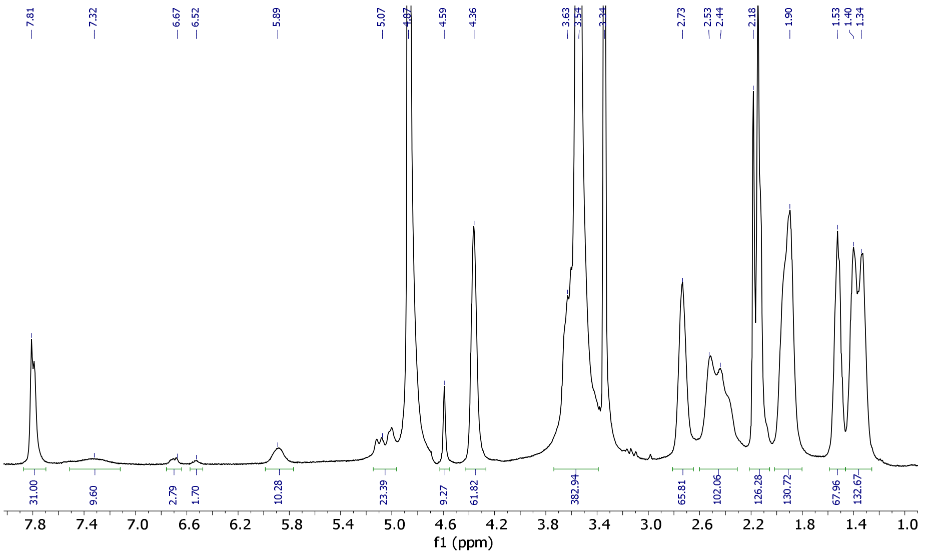

Figure S41. ^{1}H NMR (400 MHz, CD$_3$OD) spectrum of PMeOx$_{52}$-PPentynOx$_{32}$(OH)$_{32}$-PButenOx$_{13}$(Cat)$_2$.

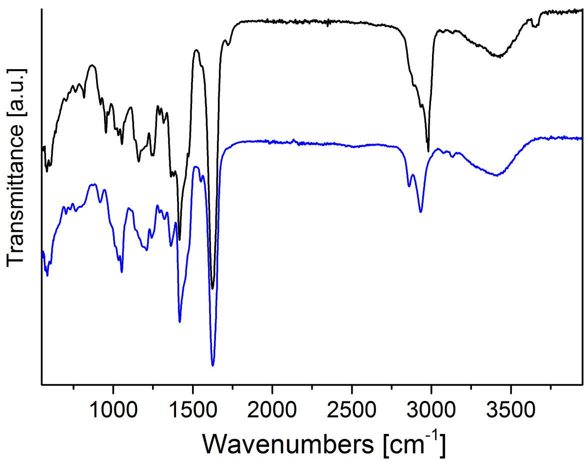

Figure S42. ATR-IR spectra of $PMeOx_{52}$-$PPentynOx_{32}(OH)_{32}$-$PButenOx_{13}$ (black line) and the corresponding catechol-functionalized $PMeOx_{52}$-$PPentynOx_{32}(OH)_{32}$-$PButenOx_{13}(Cat)_2$ (blue line).

5.2.3 Thioester-modification

The thioester-modification was performed according to the general procedure of thiol-ene click reaction as described above (Section 5.2, page 30).

P3-OH-SAc = $PMeOx_{52}$-$PPentynOx_{32}(OH)_{32}$-$PButenOx_{13}(SAc)_{13}$

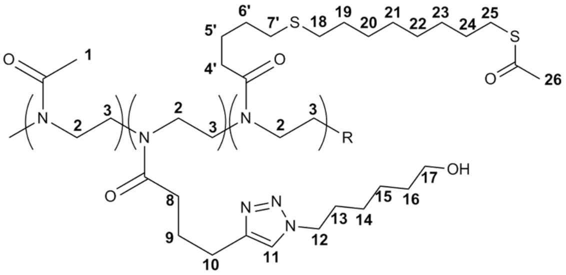

$PMeOx_{52}$-$PPentynOx_{32}(OH)_{32}$-$PButenOx_{13}$ (4 µmol, 56 mg), *S*-(8-mercaptooctyl)ethanethioate (96 µmol, 21.1 mg,), DMPA (2 mg). Yield 69 mg. **^{1}H NMR** (400 MHz, $CDCl_3$): δ (ppm) = 7.82 (m, 31.4H, H^{11}), 4.29 (m, 65.3H, H^{12}), 3.45 (m, 456H, $H^{2\text{-}3,17}$), 2.94-2.77 (m, 122H, $H^{8,25}$), 2.77-2.60 (m, 93H, $H^{7',18}$), 2.57-2.42 (m, 86H, $H^{4',10}$), 2.31 (m, 43H, H^{26}), 2.10 (m, 161H, H^{1}), 1.99-1.77 (m, 144H, $H^{9,13}$), 1.86-1.42 (m, 199H, $H^{5',6',16,19}$), 1.41-1.20 (m, 308H, $H^{14,15,20\text{-}23}$). **ATR-IR:** 1690 cm^{-1} ν(SAc - carbonyl vibration).

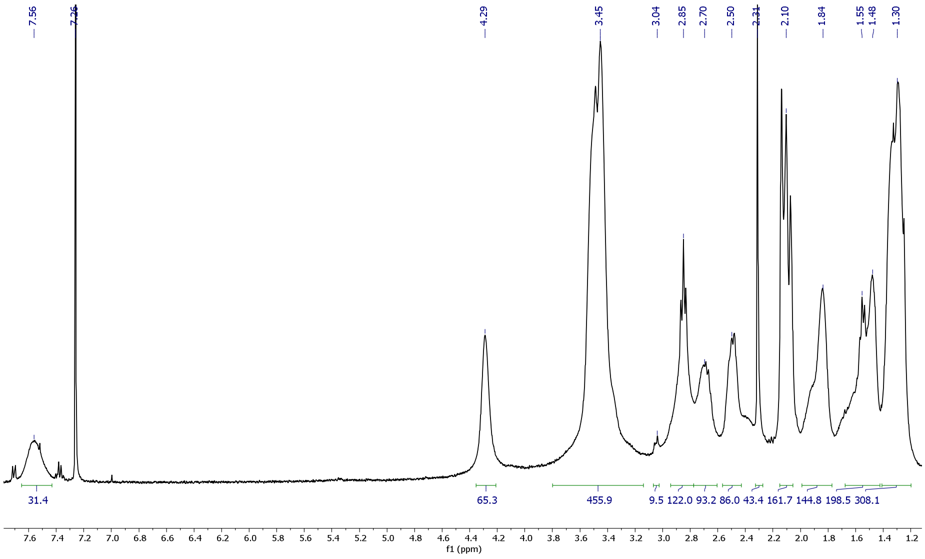

Figure S43. ^{1}H NMR (400 MHz, CD_3OD) spectrum of $PMeOx_{52}$-$PPentynOx_{32}(OH)_{32}$-$PButenOx_{13}(SAc)_{13}$.

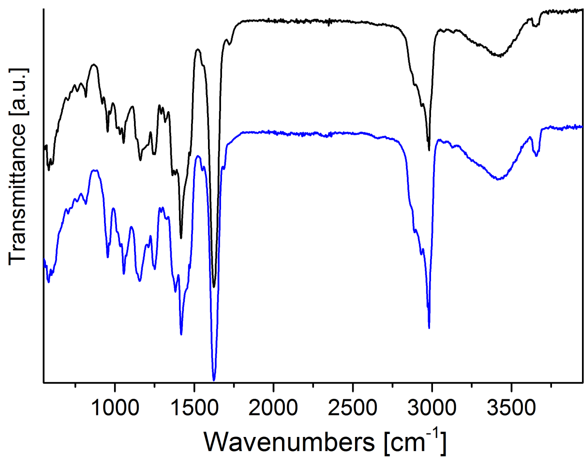

Figure S44. ATR-IR spectra of $PMeOx_{52}$-$PPentynOx_{32}(OH)_{32}$-$PButenOx_{13}$ (black line) and the corresponding thioester-functionalized $PMeOx_{52}$-$PPentynOx_{32}(OH)_{32}$-$PButenOx_{13}(SAc)_{13}$ (blue line).

P3-COOH-SAc = $PMeOx_{52}$-$PPentynOx_{32}(COOH)_{26}$-$PButenOx_{13}(SAc)_{13}$

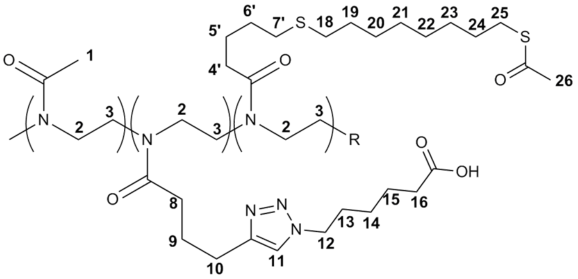

$PMeOx_{52}$-$PPentynOx_{32}(COOH)_{26}$-$PButenOx_{13}$ (8 µmol, 102 mg), *S*-(8-mercaptooctyl)ethanethioate (192 µmol, 42.2 mg). DMPA (2 mg). Yield 126 mg. **^{1}H NMR** (400 MHz, CD_3OD): δ (ppm) = 7.82 (m, 25H, H^{11}), 4.37 (m, 54H, H^{12}), 3.72 (s, 74H), 3.54 (m, 289H, H^{2-3}), 2.96-2.83 (m, 27H, H^{25}), 2.82-2.66 (m 67H, $H^{8,18}$), 2.65-2.23 (m, 201H, $H^{4',7',10,16,26}$), 2.22-2.09 (m, 156H, H^1), 2.04-1.83 (m, 116H, $H^{9,13}$), 1.81-1.52 (m, 146H, $H^{5',6',14,15,19}$), 1.52-1.24 (m, 152H, H^{20-24}). **ATR-IR:** 638 cm^{-1} ν(C_{sp}-H wagging mode), 1690 cm^{-1} ν(SAc - carbonyl vibration), 3261 cm^{-1} ν(C_{sp}-H mode)

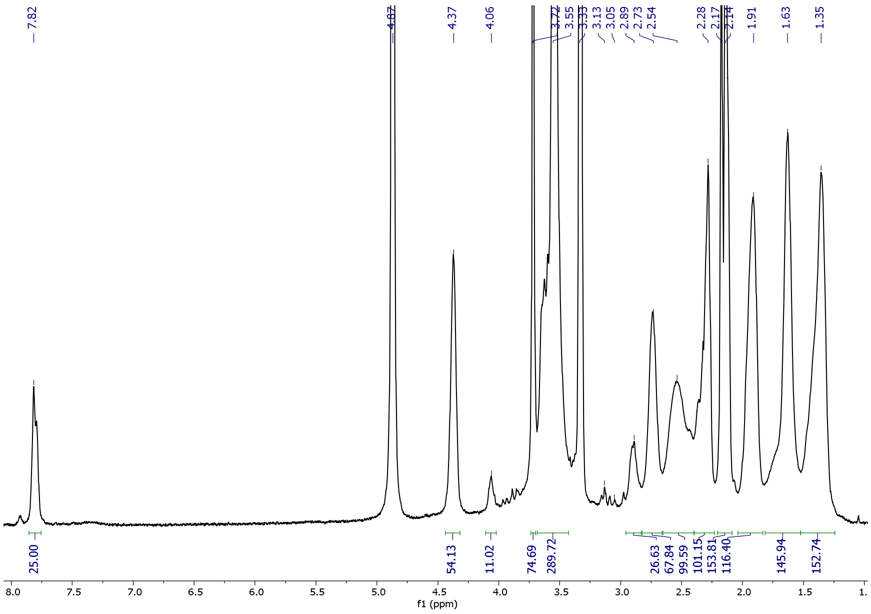

Figure S45. ^{1}H NMR (400 MHz, CD_3OD) spectrum of $PMeOx_{52}$-$PPentynOx_{32}(COOH)_{26}$-$PButenOx_{13}(SAc)_{13}$.

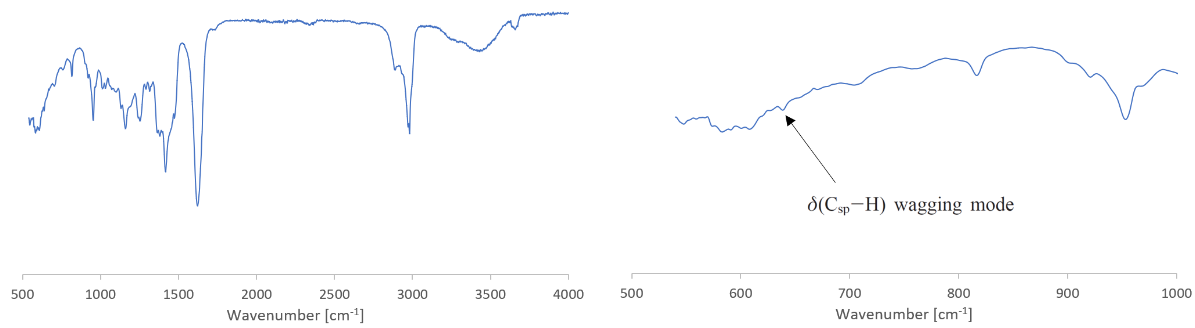

Figure S46. ATR-IR spectra of the thioester-functionalized PMeOx$_{52}$-PPentynOx$_{32}$(COOH)$_{26}$-PButenOx$_{13}$(SAc)$_{13}$. The zoom section exhibits a small peak at 638 cm^{-1} which might arise from C≡H modes. Combined with the shoulder around 3260 cm^{-1}, this gives rise to the conclusion that unmodified pentynyl functions at least partially survive the TE-reaction conditions in the 2.PPM.

5.2.4 Siloxane-modification

The siloxane-modification was performed according to the general procedure of thiol-ene click reaction as described above (Section 5.2, page 30). The crude reaction mixture was purified by stepwise dialysis against methanol and water. A colorless solid was isolated after freeze-drying. NMR analysis of this substance failed since the solid was hardly soluble in common deuterated solvents.

P3-OH-SiOMe₃ = PMeOx$_{52}$-PPentynOx$_{32}$(OH)$_{32}$-PButenOx$_{13}$(SiOMe$_3$)$_x$

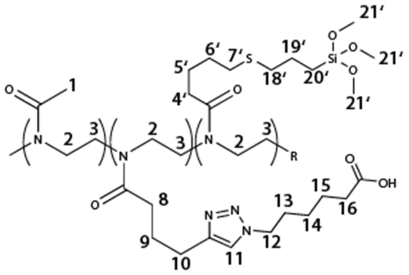

PMeOx$_{52}$-PPentynOx$_{32}$(OH)$_{32}$-PButenOx$_{13}$ (5 µmol, 68 mg), (3-mercaptopropyl)trimethoxysilane (117 µmol, 23 mg), DMPA (2 mg). Yield 80 mg. **^{1}H NMR** (400 MHz, CDCl$_3$): δ (ppm) = 3.45 (m, 421H, H$^{2-3}$), 2.10 (m, 153H, H^1).

These signals might originate from the hydrophilic polymethyl-2-oxazoline block, but no further signals could be unambiguously assigned to the polymer. The poor solubility and the absence of most of the polymer NMR signals gives rise to the conclusion that the reactive methoxysilane groups were hydrolyzed and crosslinked under the formation of silica materials.

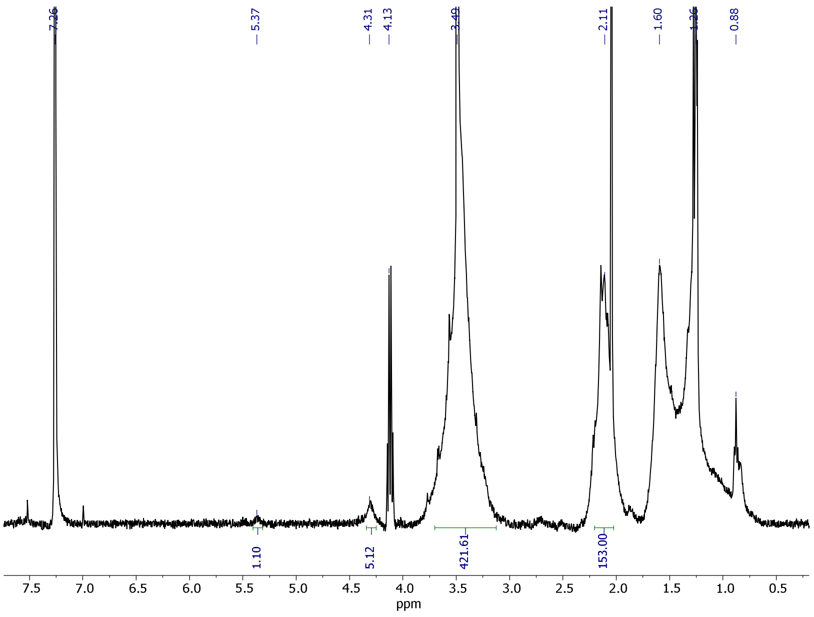

Figure S47. ^{1}H NMR (400 MHz, CDCl$_3$) spectrum of PMeOx$_{52}$-PPentynOx$_{32}$(OH)$_{32}$-PButenOx$_{13}$(SiOMe$_3$)$_x$.

P1-COOH-SiOMe$_3$ = PMeOx$_{43}$-PPentynOx$_{11}$(COOH)$_{11}$-PButenOx$_{11}$(SiOMe$_3$)$_6$

The siloxane-modification was repeated according to the general procedure of thiol-ene click reaction as described above (Section 5.2, page 30) without dialysis against water in order to avoid the hydrolysis of the methoxysilane groups. After the exposure to UV irradiation, the solvent was removed from the reaction mixture. The colorless sediment was re-dispersed in anhydrous acetonitrile to dissolve the excess 3 mercaptopropyl trimethoxysilane while the polymer is only poorly soluble in acetonitrile. The dried colorless solid was analyzed by NMR (Figure S48) suggesting the attachment of 6 methoxysilane molecules and a final polymer structure of PMeOx$_{43}$-PPentynOx$_{11}$(COOH)$_{11}$-PButenOx$_{11}$(SiOMe$_3$)$_6$. Moreover, traces of the 3-(mercaptopropyl)trimethoxysilane starting material were identified as well. IR analysis was not performed as the trimethoxysilane group is sensitive towards moisture.

PMeOx$_{43}$-PPentynOx$_{11}$(COOH)$_{11}$-PButenOx$_{11}$ (17 µmol, 150 mg), (3-mercaptopropyl)trimethoxysilane (156 µmol, 31 mg), DMPA (2 mg). Yield 80 mg. **^{1}H NMR** (400 MHz, CD$_3$OD): δ (ppm) = 7.90 (m, 11.6H, H^{11}), 5.87 (m, 4H, H^{6}), 5.02 (m, 8H, H^{7}), 4.40 (m, 29H, H^{12}), 3.65 (s, 53H, H$^{21/21'}$), 3.54 (m, 232H, H$^{2-3}$), 2.95-2.83 (m, 16H, H$^{18'}$), 2.83-2.69 (m 33H, H^{8,18}), 2.62-2.26 (m, 84H, H$^{4',4,5,7',10,16}$), 2.14 (m, 148H, H^{1}), 2.03-1.86 (m, 73H, H$^{9,13,19'}$), 1.72-1.35 (m, 58H, H$^{5',6',14,15,19}$, 0.97-0.63 (m, 22H, H$^{20/20'}$).

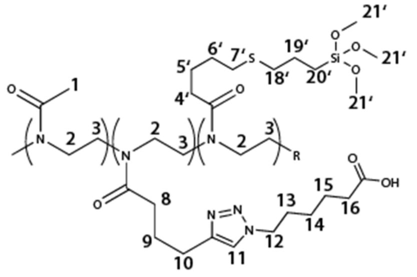

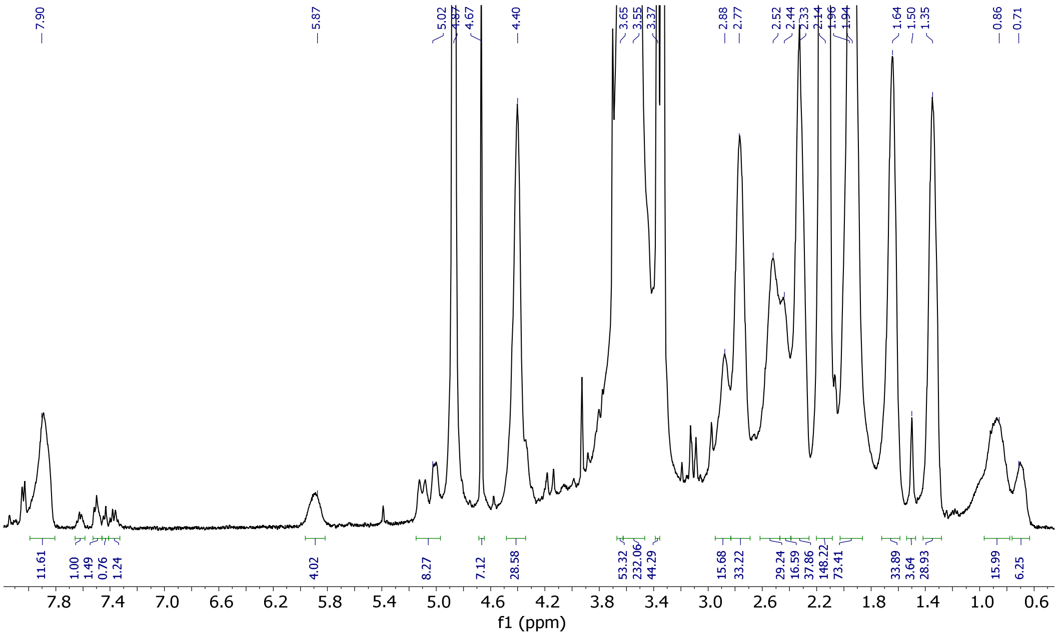

Figure S48. ^{1}H NMR (400 MHz, CD$_3$OD) spectrum of PMeOx$_{43}$-PPentynOx$_{11}$(COOH)$_{11}$-PButenOx$_{11}$(SiOMe$_3$)$_6$.

5.3 3. PPM: Palladium(0) catalyzed C-C cross coupling

Palladium-catalyzed coupling reactions offer great potential to orthogonally post-modify triple or double bonds with halides, depending on the reaction conditions. Sonogashira and Heck coupling reactions were performed on poly(2-oxazoline) triblock copolymers with alkyne and olefin segments in initial experiments.

5.3.1 Sonogashira coupling reaction

The reaction of aromatic halides and $PMeOx_{50}$-$PPentynOx_{9}$-$PButenOx_{9}$ was proceeded (Scheme S2), as described by the group of Onimura (see experimental procedure below). [8] The model typ *para*-iodo trifluorotoluene was applied based on its free and unique NMR proton and fluor signals.

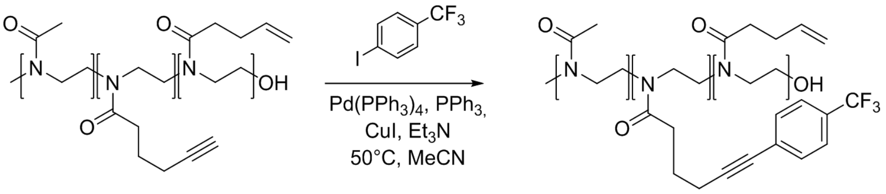

Scheme S2. Palladium(0) catalyzed C-C cross-coupling reaction (Sonogashira) between alkynes and iodides.

<u>Experimental Procedure:</u>

The following reaction was executed under inert conditions. $PMeOx_{50}$-$PPentynOx_{9}$-$PButenOx_{9}$ (**P3**) (0.5 g, 0.075 mmol), *p*-iodo trifluorotoluene (1.85 mL, 0.697 mmol,) and triethylamine (0.726 mL, 19.41 mmol) were dissolved in anhydrous acetonitrile (15 mL) and degassed. CuI (3 mg, 0.017 mmol), $Pd(PPh_3)_4$ (20mg, 0.017 mmol) and PPh_3 (9 mg, 0.034 mmol) were added and the reaction mixture was stirred for 21 days at 50 °C. The final product was obtained via stepwise dialysis against acetonitrile and water.

The conversion was monitored using NMR analysis. Thus, aliquots after 8, 11 and 21 days and of the final isolated powder after dialysis were analyzed (Figure S49). The NMR signal of *para*-iodo trifluorotoluene (two doublets at 7.4 and 7.7 ppm) decrease with increasing reaction time and fully vanish after dialysis. Simultaneously, new signals appear in the range between 7.44 and 7.78 ppm, which are still present after dialysis. This suggests the development of a new aromatic species during the concentration of the iodo-derivate decreases, which might be assigned to the targeted phenylacetylene functionality (marked in green). Moreover, the corresponding signals of triphenylphosphine (marked in pink) disappear as well after dialysis.

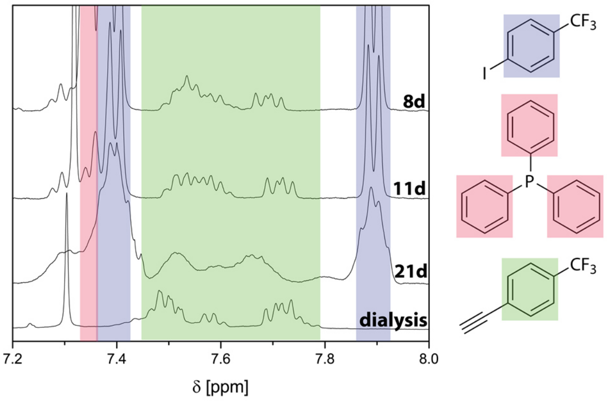

Figure S49. ^1H NMR (400 MHz, $CDCl_3$) of the isolated Sonogashira reaction product.

However, reliable values were not obtain by NMR integral analysis and thus the conversion of the Sonogashira coupling reaction and furthermore the degree of functionalization could not be quantified.

5.3.2 Heck coupling reaction

The palladium(0)-catalyzed Heck coupling reaction of PMeOx$_{57}$-PButenOx$_8$ with methyl-4-iodobenzoate (Scheme S3) was performed in a microwave according to the protocol of Ogino *et al.* (see experimental procedure below).[9]

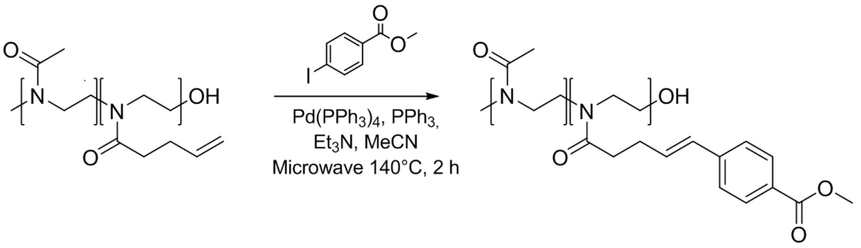

Scheme S3. Palladium(0) catalyzed C-C cross-coupling reaction (Heck) between olefins and iodides.

<u>Experimental procedure:</u>

The following reaction was executed under inert conditions. In a microwave reaction vessel, PMeOx$_{57}$-PButenOx$_8$ (0.130 g, 0.022 mmol) and methyl 4-iodobenzoate (0.093 g, 0.353 mmol) were dissolved in anhydrous acetonitrile (10 mL). After the addition of triethylamine (0.049 mL, 0.353 mmol) and Pd(PPh$_3$)$_4$ (catalytic amounts), the reaction mixture was degassed and then heated to 140 °C overnight. Purification via dialysis in acetonitrile and water yielded the final product as a brown powder.

NMR spectroscopy was used to analyze the product revealing a broad peak at 6.44 ppm (integral 3), which potentially arises from vinyl proton H7' (Figure S50). Furthermore, a bunch of new signals appeared at 3.93 (integral 9), 7.44 (integral 15) and 7.92-8.11 (integral 11.5), which might be assigned to the methyl group (3.93 ppm, *9'*) and the aromatic ring protons (7.44 ppm, *8'*) of the benzoate-functionalized polymer. Moreover, halide starting material (signals at 7.70 and 3.89 ppm) as well as small amounts of non-removed triphenylphosphine (7.38 ppm) are still present.

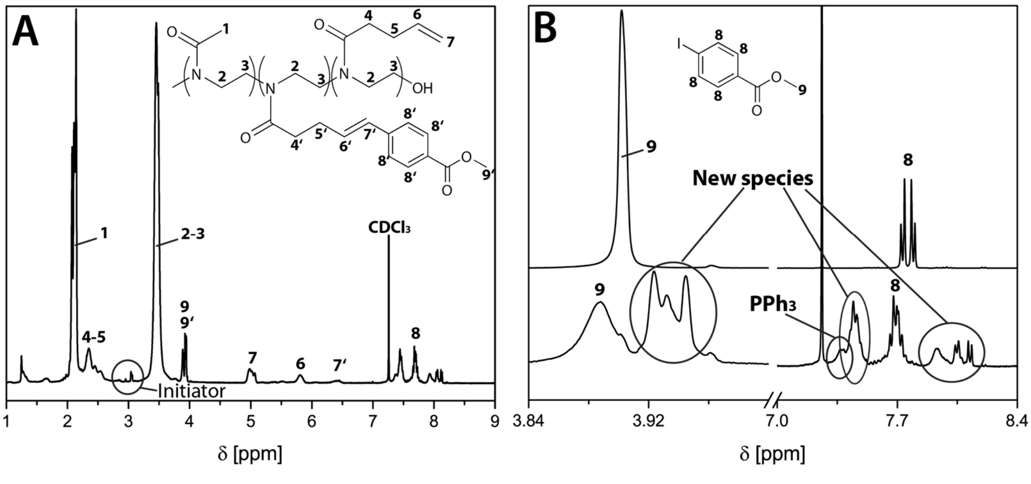

Figure S50. ^{1}H NMR (400 MHz, $CDCl_3$) spectrum of the Heck coupling product of PMeOx$_{57}$-PButenOx$_8$ with methyl-4-iodobenzoate (**A**) and in comparison with the methyl 4-iodobenzoate (**B**).

NMR indicated the linkage of three benzoate molecules onto the macromolecular backbone with the composition PMeOx$_{57}$-PButenOx$_8$(benzoate)$_3$. Nevertheless, detailed analysis is required to fully characterize and evidence the Heck coupling although the first results are promising.

6 Synthesis of polymer-coated nanoparticles

6.1 Gold

6.1.1 Synthesis of gold nanocubes

The gold nanocubes (AuNCs) were synthesized via a seed-mediated pathway based on the separation of crystal nucleation and particle growth for the precise morphological control of the obtained nanoparticles. Cetylpyridinium chloride (CPC) was applied as surfactant according to a procedure as described by Kirner *et al.*[10] TEM analysis shows the presence of single crystalline, cubic gold particles with high monodispersity and average distribution of edge lengths of 34.2 nm. The formation of self-organized gold layers is irrelevant for this topic.

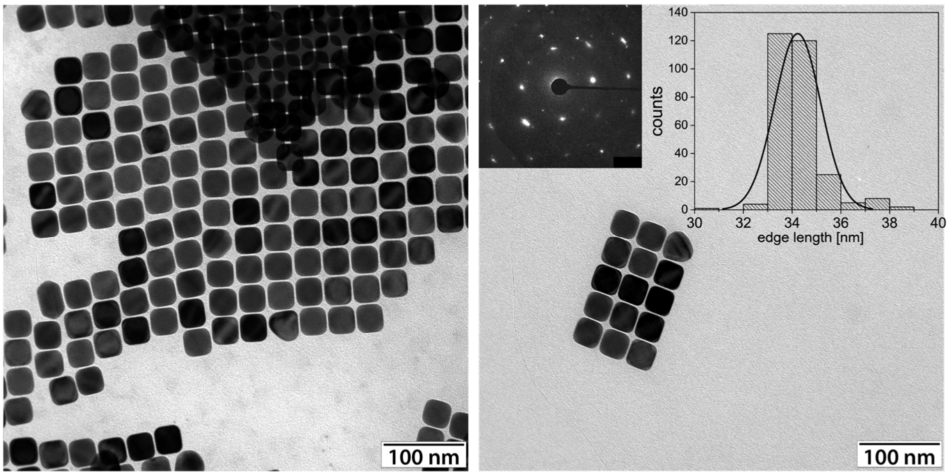

Figure S51. TEM images of synthesized gold nanocubes via seed-mediated procedure.

6.1.2 Surface-modification of gold nanocubes

The polymer $PMeOx_{52}$-$PPentynOx_{32}(COOH)_{26}$-$PButenOx_{13}(SAc)_{13}$ was dissolved in MilliQ water (resistivity < 18.2 µS/cm) with polymer concentrations of 0.1, 0.5 and 1.0 mg/mL. To 500 µL of a freshly prepared AuNC dispersion 500 µL of the polymer dispersions was added and stirred overnight at RT. Here it is worth to mention, that the polymer concentration is divided in half. After adding 12 mL MilliQ water, the dispersions were centrifuged (13 mL H_2O, 9000 rpm, 10 min each) and the sediment was re-dispersed in 2 mL MilliQ water and examined via complementary analytical techniques (see main text Figure 3).

TEM analysis (main text, Figure 3A and B) illustrates organic coatings around the nanoparticles over the whole sample. Some nanoparticles self-assemble into smaller clusters, mainly dimers, but also higher oligomer formations. As UV/Vis-spectroscopy (Figure S52) and AUC analysis (main text, Figure 3C) suggest the colloidal stability of these organic-inorganic composites in water, these cluster formations are most likely triggered by the preparation of the sample for ex-situ TEM analysis.

Moreover, AUC sedimentation velocity experiments (main text, Figure 3C) show little shifts of the hydrodynamic diameters of the polymer gold conjugates (main diameter of 31.5 nm) compared to the initial CPC-functionalized gold nanocubes (33 nm). Based on the density and friction dependence of the sedimentation behavior, modified particle surface might affect a different sedimentation behavior due to the coronal environment. This suggests the polymer adsorption via surfactant exchange or co-adsorption. It is noteworthy, that the sedimentation coefficient distribution was transformed into the D_H-distribution by assuming the gold density of 19.32 g/mL (also for the less dense polymer corona) and

the density of the polymer dispersion was assumed as water density. This explains the negligible deviation of the AuNC size (33 nm) from TEM counts (34.2 nm).

In addition, zeta potential measurements (main text, Figure 3D) point towards the polymer adsorption of both macromolecules since the surface charges of AuNC (42 mV) have been reduced in the presence of the different polymers. The effect of carboxylate segment impacts the charge reduction to more extent, due to the lower potential of negatively charged carboxylates (- 6 mV, pure polymer).

Finally, it remains unclear whether the CPC stabilization layer have been substituted or supplemented by the new macromolecules. In fact, combined analytical results give rise to the conclusion that the SAc containing polymer is present on the surface.

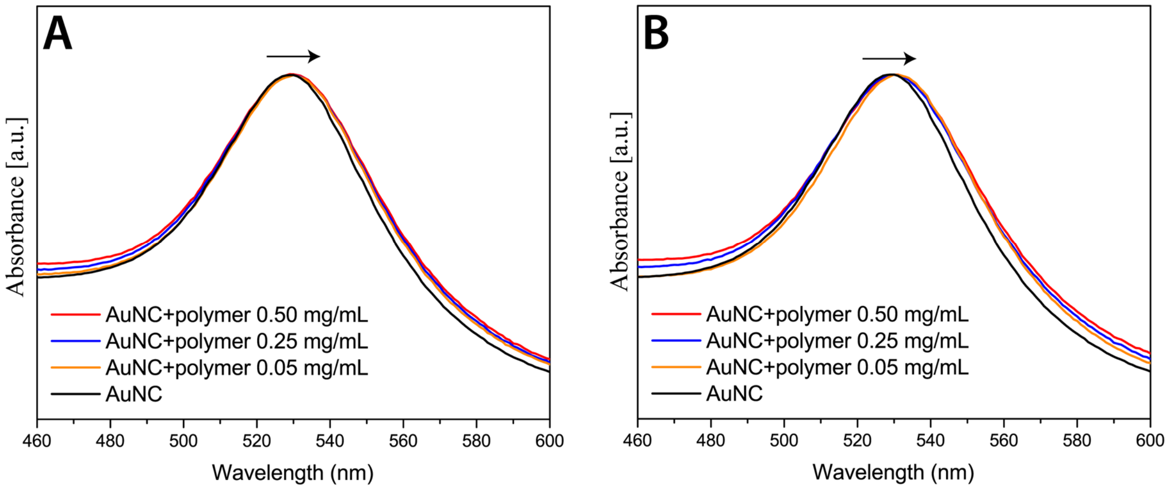

Figure S52. Absorbance-normalized UV/Vis spectra of AuNC polymer mixtures of **P3-OH-SAc** (A) and **P3-COOH-SAc** (B).

6.2 Iron oxide

6.2.1 Synthesis of iron oxide nanoparticles

The iron oxide nanoparticles (IONPs) have been synthesized, according to the synthesis protocol of Kang *et al.* [11] Thus, 9 nm sized Fe_3O_4 particles were obtained in water by co-precipitation of Fe^{2+} and Fe^{3+} species at high pH values using sodium hydroxide solution (Figure S53). It is worth to mention that those nanoobjects are stabilized in water by the negatively charged oxygen atoms at the surface of the IONPs and not by surfactant. However, the colloidal IONP tends to cluster formation, as discussed in the AUC part (Figure 54).

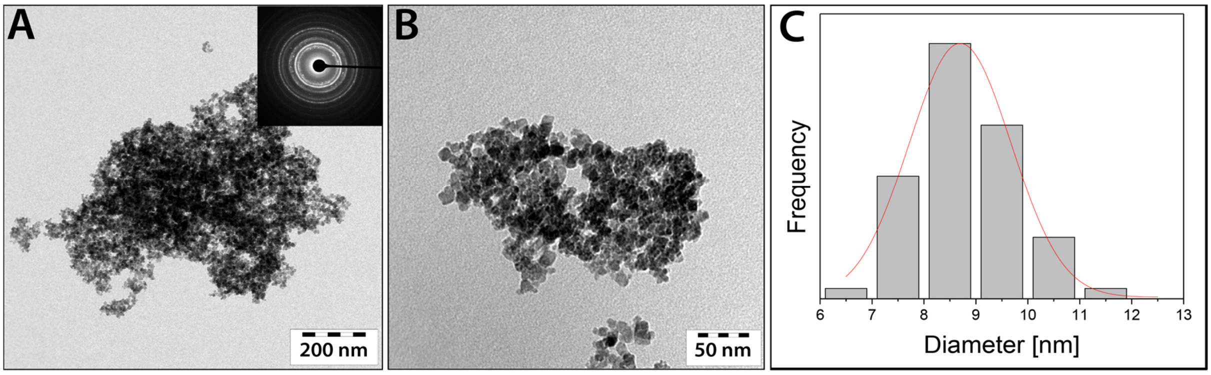

Figure S53. (A) and (B) depict TEM analysis of an aqueous dispersion of iron oxide nanoparticles (IONPs) and the size distribution (C), as synthesized according to a protocol of Kang *et al.*[11]

6.2.2 Surface-modification of iron oxide nanoparticles

The polyoxazoline copolymers (1 mg) $PMeOx_{52}$-$PPentynOx_{32}(OH)_{32}$-$PButenOx_{13}(COOH)_{13}$ (**P3-OH-COOH**) and $PMeOx_{52}$-$PPentynOx_{32}(OH)_{32}$-$PButenOx_{13}(Cat)_2$ (**P3-OH-Ph(OH)$_2$**) were dissolved in degassed water (5 mL) and the solution was further degassed overnight using a N_2-streamed syringe needle. After adding 10 mg of the freeze-dried IONPs and degassed NaOH solution to adjust the pH to 9, the solution was stirred under N_2-atmosphere at room temperature overnight. The black reaction mixture was precipitated by adding a magnet at the side of the reaction vessel and the dispersion turned translucent. The supernatant was completely separated in order to remove the non-bound polymer. 2 mL of degassed water were added to yield the polymer-coated iron oxide particles. It is worth noting, that the particle dispersion did apparently not suffer from major oxidation to maghemite Fe_2O_3, since the dispersion was still black instead of brown. The re-dispersed mixture was used to study the resultant IONPs-polymer composites (Figure S54).

TEM analysis (Figure S54, A and B) exhibits the nanoparticle subunits that are similarly shaped like the native IONPs (Figure S53) in case for both polymers. Nevertheless, the formation of aggregates possibly during the drying process can be observed. The existence of a lower contrast shell around the IONPs indicates the presence of non-removed organic material and thus suggests the interfacial activity of both polymers with the IONPs.
AUC sedimentation velocity experiments (Figure S54) reveal distinct species that sedimented already in lower centrifugal fields (2000 rpm). No additional matter could be found with higher centrifugal fields up to 60000 rpm by either detecting absorption nor refractive index. It is worth to mention that the native polymers usually would need 60000 rpm for the sedimentation process giving rise to the assumption that non-bound polymer was completely removed in the purification step. The analysis of the raw data suggests the presence of small IONP clusters with diameters between about 48-62 nm (black curve), which are significantly larger than the freshly synthesized 9 nm IONPs. Obviously, the magnetic attraction is much stronger than the electrostatic stabilization of the particles.

After adding **P3-OH-COOH** and **P3-OH-Ph(OH)**$_2$, respectively, the defined IONP cluster formation was retained, although the major diameters are increased to 66 and 78 nm. To calculate the diameter from the obtained distribution of the sedimentation coefficient s, the density of iron oxide of 5.17 g/cm^3 was used, neglecting that the density of the polymeric corona is different. For the solvent density 0.998 g/mL was assumed. Finally, AUC analysis suggested the presence of the polymer-IONP cluster that is stabilized in a short timescale in aqueous solution.

The amount of organic material that is attached on the IONPs was quantified by thermogravimetric analysis (TGA) revealing 4.5% for **P3-OH-COOH** and 15.5% for **P3-OH-Ph(OH)**$_2$, respectively. The polymeric quantity on the IONP is significantly higher in case of the catechol-polymer, although only two catechol functional groups are present on the polymer in average compared to the 13 carboxyl groups. This is in good agreement with literature describing the higher adsorption affinity of catechol groups to IONPs surfaces as compared to carboxylates.[12]

These combined results demonstrate the capabilities of using tailor-made macromolecules for surface modification of iron oxide nanoparticles ensuring their morphological stability.

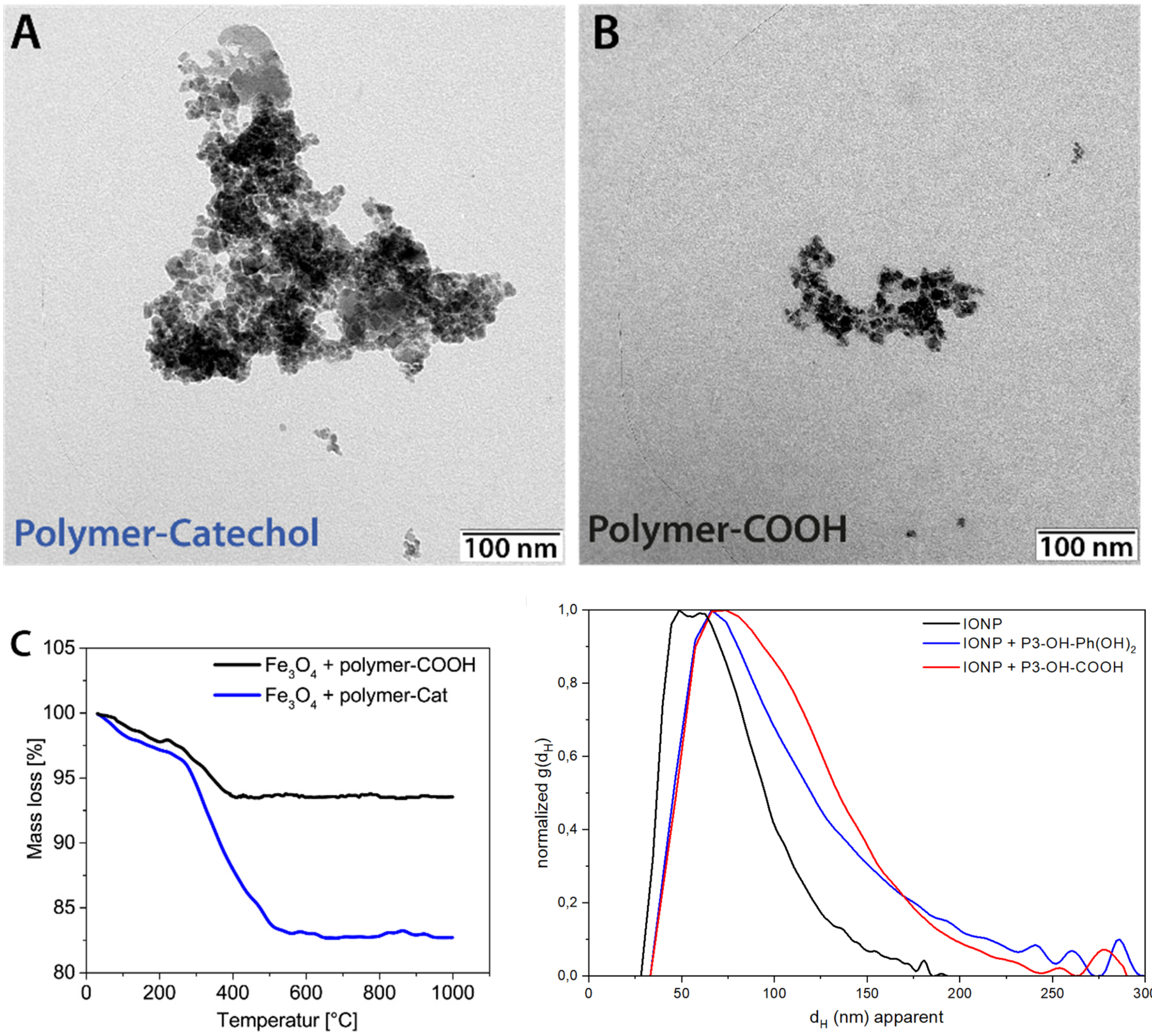

Figure S54. Analysis of purified polymer-IONP mixtures. TEM analysis of (A) IONP + **P3-OH-Ph(OH)**$_2$ and (B) IONP + **P3-OH-COOH**. Thermo-gravimetric analysis of IONP + **P3-OH-COOH** (blue curve) and **P3-OH-Ph(OH)**$_2$ (black curve) (C) and (bottom left) AUC sedimentation experiments as-synthesized IONP (black curve) IONP + **P3-OH-Ph(OH)**$_2$ (blue curve) and (B) IONP + **P3-OH-COOH** (red curve).

6.3 Silica Nanoparticles

The last exemplary application of the poly(2-oxazoline) toolkit aimed at polymer-coated silicate nanoparticles. In this case, a different approach is pursued since the functionalization of the silica nanoparticles is achieved by using the selected polymer PMeOx$_{43}$-PPentynOx$_{11}$(COOH)$_{11}$-PButenOx$_{11}$(SiOMe$_3$)$_6$ as macromolecular precursor for the in-situ synthesis of polymer-coated SiO_2-nanoparticles. Two different synthesis procedures were employed to trigger hydrolysis and condensation of the pendent trimethoxysilyl moieties as requisite reaction for silicate formation.[13] On the one hand, a solvent exchange method using a dialysis membrane and on the other hand the Stöber synthesis were applied. In this manner, 30 and 15 nanometer-sized objects were synthesized, respectively, according to DLS analysis.

6.3.1 Dialysis-triggered silica formation

A methanol solution of the trimethoxysilane-modified polymer PMeOx$_{43}$-PPentynOx$_{11}$(COOH)$_{11}$-PButenOx$_{11}$(SiOMe$_3$)$_6$ was dialyzed against water using a gradual and slow solvent exchange. The gradual dilution with water destabilizes the alkoxysilane precursors triggering hydrolysis and condensation reactions of alkoxysilane precursors, and thus, leading to the formation of silica particles (Scheme S4). The dialyzed opaque solution was freeze-dried and a colorless powder was obtained.

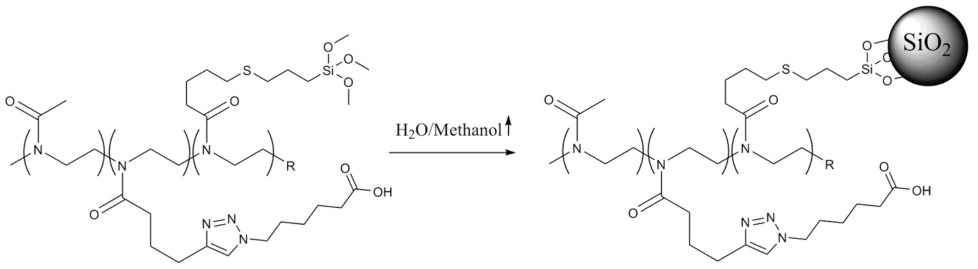

Scheme S4. Possible formation of polymer-coated silica particles from trimethoxysilane-modified poly(2-oxazoline)s.

The NMR analysis of the isolated and poorly soluble powder (Figure S55, left, in methanol-*d4*) shows that all corresponding signals of the inner PButenOx segment have disappeared. This includes most signals of the non-reacted free olefins as well after thiol addition. This absence points towards the insolubility of the PButenOx(SiOMe$_3$) segment in common NMR solvents and further suggest the presence of insoluble silica materials with a methanol-*d4* soluble polymer corona. The presence of the prominent signal (H21' at 3.73 ppm) can be referred to the methoxy-silane function. This suggests that non-reacted, alkoxysilane functionalities are still present on the polymer.

ATR-IR spectroscopic analysis of the freeze-dried powder (Figure S55, right) illustrates Si-O-Si bending and stretching vibrations at 816 and 953 cm^{-1}, respectively.[14] Group vibrations at 919 and 1010 cm^{-1} can be referred to alkoxysilane vibrations. This indicates the coexistence of silica material and non-reacted alkoxysilane-precursors on the polymers. It is worth mentioning that the absence of S-H vibrations at 2670 cm^{-1} is a strong hint that indicates the absence of thiol-functionalized silica particles (Silica-SH). This suggests the complete removal of the thiol-silane starting material.

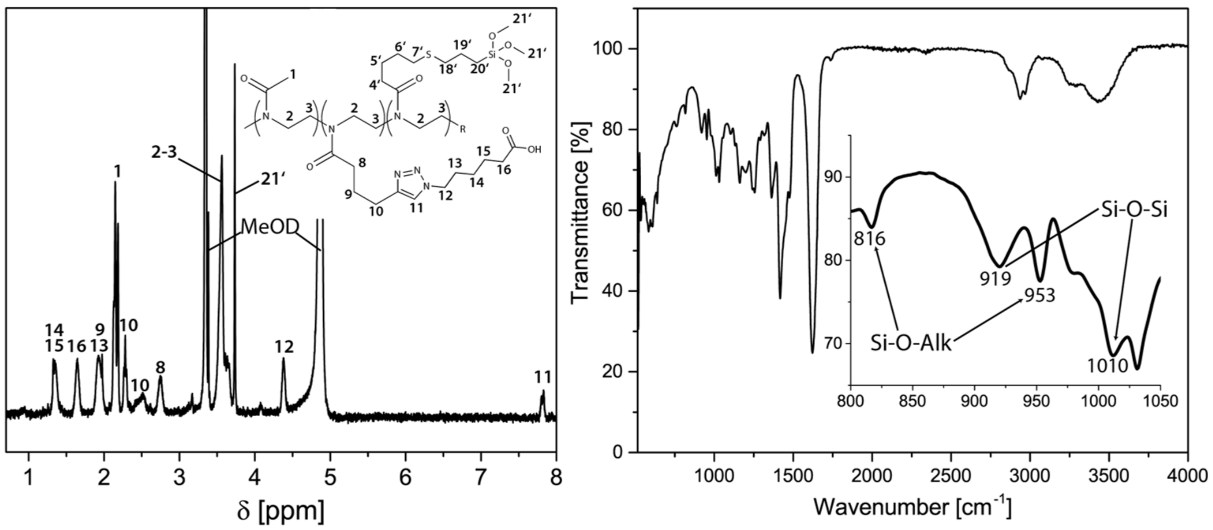

Figure S55. ^{1}H NMR (400 MHz, CD_3OD) spectrum (left) and ATR-IR spectrum (right) of $PMeOx_{43}$-$PPentynOx_{11}(COOH)_{11}$-$PButenOx_{11}(SiOMe_3)_6$ after dialysis against water.

The freeze-dried powder after dialysis against water was re-dispersed in water (0.5 mg/mL) and the aqueous dispersion was analyzed by DLS showing the presence of 30 nm nanoparticles (Figure S56). The DLS analysis indicates the presence of polymer coated silica nanoparticles with defined shapes instead of aggregated silica bulk material.

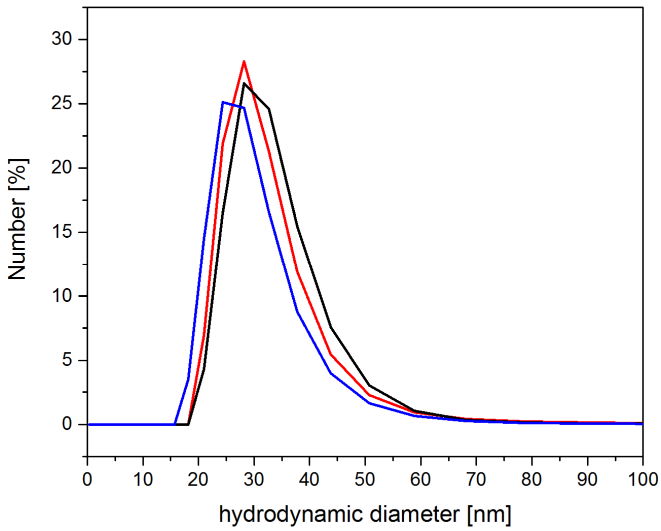

Figure S56. DLS measurement (triplicate) of aqueous solution of $PMeOx_{43}$-$PPentynOx_{11}(COOH)_{11}$-$PButenOx_{11}(SiOMe_3)_6$ prepared by gradual dialysis against water.

6.3.2 Silica formation by a Stöber process

The dialysis-triggered formation of silica nanoparticles revealed the high reactivity of the methoxysilane groups attached to the polymer. Therefore, the formation of silica particles is supposed to proceed by using the Stöber process as well, however, with better control.[15–17] Tetramethoxysilane (TMOS) was used in the first attempts as silica source in order to synthesize silica nanoparticles, as described by Takeda *et al*.[13] Different solvent ratios were tested (Table S2) in order to control the particle formation, since the proportion of water and methanol as well as the ammonia concentration represent key parameters for morphological and size control.

Table S2. Stöber synthesis of silica particles by varying the composition of the solvent.

Entry	V_{TMOS} [mL]	V_{water} [mL]	$V_{methanol}$ [mL]	Molar ratio H_2O/MeOH	$V_{ammonia}$ [mL]
**1**	0.015	2.34	0.53	10/1	0.05
**2**	0.015	2.25	0.73	7/1	0.05
**3**	0.015	2.15	0.97	5/1	0.05
**4**	0.015	1.93	1.45	3/1	0.05
**5**	0.015	1.29	2.90	1/1	0.05
**6**	0.015	0.86	3.87	1/2	0.05

DLS analysis of batch number 4 (Table S2) revealed intensity-weighted size of 3.7 nm (12%) and 157 nm (88%), a polydispersity of 0.2 and a number weighted size distribution of 3 nm (Figure S57, black line). Instead, DLS analysis of the dispersions using different conditions (entries 1-3 and 5-6) suggest nano-objects with inappropriate deviations and therefore, they have been discarded.

For the Stöber synthesis of polymer-coated silica nanoparticles using $PMeOx_{43}$-$PPentynOx_{11}(COOH)_{11}$-$PButenOx_{11}(SiOMe_3)_6$ as macromolecular precursor, the conditions of entry 4 (Table S2) have been adapted. For comparable experimental conditions, the stoichiometry of alkoxysilanes on the polymer was equal with the molar quantity of single tetramethoxysilane. Therefore, 20 mg of $PMeOx_{43}$-$PPentynOx_{11}(COOH)_{11}$-$PButenOx_{11}(SiOMe_3)_6$ was added to a solution of MilliQ (2.146 mL) and methanol (0.906 mL). The turbid dispersion was stirred for 1 h prior to the addition of ammonia solution (1 µL, 25%). After vigorous stirring for 1 h, the opaque dispersion was stirred overnight with opened cap for evaporation of methanol and ammonia. DLS analysis of the Stöber-mediated dispersion revealed number-weighted particle sizes around 14 nm (Figure S57, blue curves). Instead, Stöber-synthesis of silica particles using TMOS as silica source results in a dispersion with 3 nm-sized objects (Figure S57, black curves). Since the amphiphilic block copolymer itself tends to self-organize into core shell nanoparticles, the non-silane modified polymer dispersion of $PMeOx_{43}$-$PPentynOx_{11}(COOH)_{11}$-$PButenOx_{11}$ was analyzed by DLS elucidating clearly larger particle sizes of 20 nm (Figure S57, red curves). The combined data point to the formation of polymer-coated silica nanoparticles with a diameter of 15 nm.

In conclusion, this approach could be an alternative option to prepare silica nanoparticles with any desired modification, depending on the toolkit polymer, in this case COOH groups.

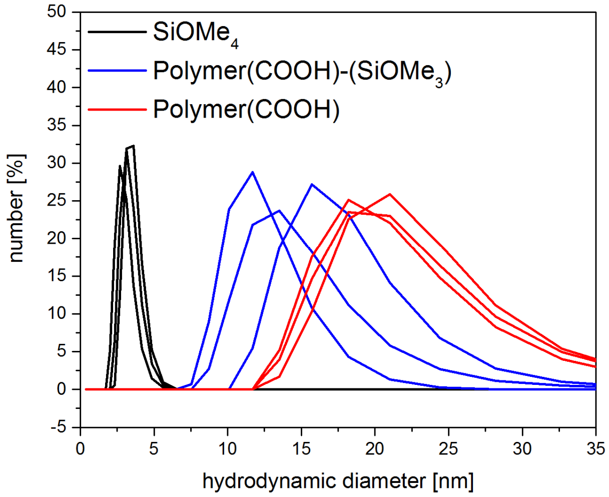

Figure S57. DLS analysis (triplicate) of silica particles using the Stöber synthesis of tetramethoxysilane (black curve) and trialkoxysilane-functionalized polymer $PMeOx_{43}$-$PPentynOx_{11}(COOH)_{11}$-$PButenOx_{11}(SiOMe_3)_6$ (blue curve) and $PMeOx_{43}$-$PPentynOx_{11}(COOH)_{11}$-$PButenOx_{11}$ (red curve).

7 References

[1] A. Gress, A. Völkel, H. Schlaad, *Macromolecules* **2007**, *40*, 7928–7933.

[2] R. Luxenhofer, R. Jordan, *Macromolecules* **2006**, *39*, 3509–3516.

[3] T. R. Dargaville, K. Lava, B. Verbraeken, R. Hoogenboom, *Macromolecules* **2016**, *49*, 4774–4783.

[4] C. Grandjean, A. Boutonnier, C. Guerreiro, J.-M. Fournier, L. A. Mulard, *J. Org. Chem.* **2005**, *70*, 7123–7132.

[5] R. Pingaew, V. Prachayasittikul, N. Anuwongcharoen, S. Prachayasittikul, S. Ruchirawat, V. Prachayasittikul, *Bioorg. Chem.* **2018**, *79*, 171–178.

[6] J. Rohrer, A. Partenhauser, S. Hauptstein, C. M. Gallati, B. Matuszczak, M. Abdulkarim, M. Gumbleton, A. Bernkop-Schnürch, *Eur. J. Pharm. Biopharm.* **2016**, *98*, 90–97.

[7] H. Veisi, R. Ghorbani-Vaghei, H. Eskandari, S. Hemmati, A. Rezaei, S. Hajinazari, M. R. H. Far, A. Entezari, *Phosphorus Sulfur Silicon Relat. Elem.* **2011**, *186*, 213–219.

[8] P. Rattanatraicharoen, K. Yamabuki, T. Oishi, K. Onimura, *Polym. J.* **2012**, *44*, 224–231.

[9] M. Ogino, Y. Taya, K. Fujimoto, *Org. Biomol. Chem.* **2009**, *7*, 3163.

[10] F. Kirner, P. Potapov, J. Schultz, J. Geppert, M. Müller, G. González-Rubio, S. Sturm, A. Lubk, E. Sturm, *J. Mater. Chem. C*, **2020**, *Advance Article.*

[11] Y. S. Kang, S. Risbud, J. F. Rabolt, P. Stroeve, *Chem. Mater.* **1996**, *8*, 2209–2211.

[12] E. Amstad, T. Gillich, I. Bilecka, M. Textor, E. Reimhult, *Nano Lett.* **2009**, *9*, 4042–4048.

[13] Y. Takeda, Y. Komori, H. Yoshitake, *Colloids Surf. A* **2013**, *422*, 68–74.

[14] G. Engelhardt, H. Kriegsmann, *Z. Anorg. Allg. Chem.* **1964**, *330*, 155–163.

[15] K. Nozawa, H. Gailhanou, L. Raison, P. Panizza, H. Ushiki, E. Sellier, J. P. Delville, M. H. Delville, *Langmuir* **2005**, *21*, 1516–1523.

[16] X.-D. Wang, Z.-X. Shen, T. Sang, X.-B. Cheng, M.-F. Li, L.-Y. Chen, Z.-S. Wang, *J. Colloid Interface Sci.* **2010**, *341*, 23–29.

[17] H. Okudera, A. Hozumi, *Thin Solid Films* **2003**, *434*, 62–68.
